# Covalent Chemical Tagging of Transmembrane Transport Proteins Illuminates the Internalization Pathways of Xenosiderophores

**DOI:** 10.1021/jacs.6c00632

**Published:** 2026-05-13

**Authors:** Minhua Cao, Marie Huynh, Inokentijs Josts, Yichong Lao, Hung H. Dang, Axia Marlin, Xuhui Huang, Henning Tidow, Eszter Boros

**Affiliations:** † Department of Chemistry, 5228 University of Wisconsin-Madison, 1101 University Avenue, Madison, Wisconsin 53705, United States; ‡ The Hamburg Advanced Research Center for Bioorganic Chemistry, Hamburg 22761, Germany; § Department of Chemistry, Institute for Biochemistry and Molecular Biology, University of Hamburg, Hamburg 22761, Germany

## Abstract

Siderophore-mediated, transmembrane uptake of iron in bacteria is a complex mechanism of essential nutrient acquisition. In addition to transporters that specifically recognize and shuttle endogenous siderophores, bacteria possess transmembrane transporters that efficiently internalize siderophore-metal complexes produced by other organisms. The discovery of transmembrane transporters using indirect methods has enabled the identification of high-affinity transporters but limits access to lower-affinity, promiscuous transport systems. Therefore, covalent tagging strategies that enable the direct identification of target proteins are desirable. To this end, we examined the co-crystal structure of **Fe-D1**, a ciprofloxacin-linked ferrioxamine complex bound to the*Pseudomonas aeruginosa* outer-membrane transporter FoxA with affinity for ferrioxamine B and E. Using a rational design approach, we identified ideal structural characteristics for ferrioxamine-based, covalent photo-cross-linker probes with reactivity toward tyrosine- and aspartate-rich binding sites within transporter domains. Fe and Ga complexes of **DFO-azir-05** and **DFO-azir-06** efficiently tag FoxA in*Escherichia coli* Lemo21 (DE3) mutants overexpressing FoxA. Subsequently, **DFO-azir-06** was successfully used to directly tag and identify the ferrioxamine-binding proteins FoxA and FpvB in*P. aeruginosa* PAO1, as well as the two main transporters of ferrioxamine B in*E. coli* K-12, FhuA and FhuE. Moreover, **DFO-azir-06** revealed a putative new role of cobalamine transporter BtuB in the transport of DFO derivatives. In conclusion, we demonstrate that careful structural design of covalent, photo-cross-linking siderophore conjugates can provide unprecedented access to the elucidation of siderophore-mediated metal ion uptake in bacteria.

## Graphical abstract

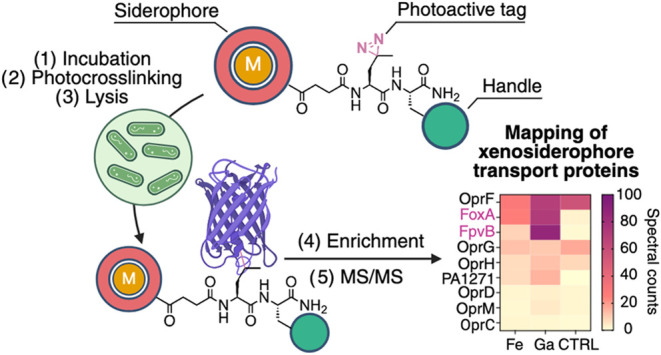

## Introduction

Metal ions constitute essential nutrients, and the regulation of bacterial metal ion homeostasis plays a pivotal role in survival within the host environment.^1,2^ However, the unbound aqua ions of many essential metals exhibit properties that result in low bioavailability.^3^ Specifically, the low solubility of Fe­(OH)_3_ (*K*_sp_ = 6.3 × 10^–38^) at pH 7.4 would result in an insufficient quantity of iron for bacteria to grow, thus bacteria rely on targeting labile iron reserves in the host environment through synthesis of metal-binding, secondary metabolites.^4,5^ Following a metal ion binding event in the extracellular host environment, ATP-dependent bacterial membrane transporters shuttle the corresponding metal complexes inside the bacterial cytoplasm, where metal ions are released by electrochemical reduction of the metal ion and/or concomitant enzymatic degradation of the siderophore.^4,6^ Siderophores can also retain affinity for nonessential xenometal ions with identical charge, comparable ionic radius, and chemical hardness to the essential metal ion. For instance, trivalent metal ions with a similar ionic radius to high-spin Fe^3+^ (0.78 Å), such as Ga^3+^ (0.76 Å), are readily transported to the bacterial peri- and cytoplasm when coordinated by endogenous, bacterial siderophores such as enterobactin or desferrioxamine.^6^ Moreover, Ga^3+^ and In^3+^-mediated growth inhibition has been observed *in vitro* and *in vivo*.^7,8^

The lack of accessible electrochemistry renders these xenometals useless for desired biological functions, which is hypothesized to contribute to their growth-inhibitory activity.^9^ Thus, xenometals are of emergent interest for the development of antibiotics that may circumvent resistance pathways using alternative mechanisms of action. However, the design of targeted compounds is hindered by a gap in knowledge of transmembrane transport proteins responsible for the cytoplasmic delivery of siderophore-metal complexes. Identification via loss of function, knockout mutants, remains the primary path to protein identification.^10^ Alternative strategies that employ affinity-based pulldown approaches have shown limited success due to the comparatively weak, noncovalent, and transient nature of coordinative and substrate binding interactions. As a result, such probes reveal high-affinity, binding proteins for endogenously produced siderophores, but lack the ability to capture others.^11,12^ As a result, the identification of the internalization machinery of synthetic siderophore-antibiotic constructs and xenosiderophores, which may utilize lower-affinity transporters, has remained elusive.^13–16^

**Figure 1:**
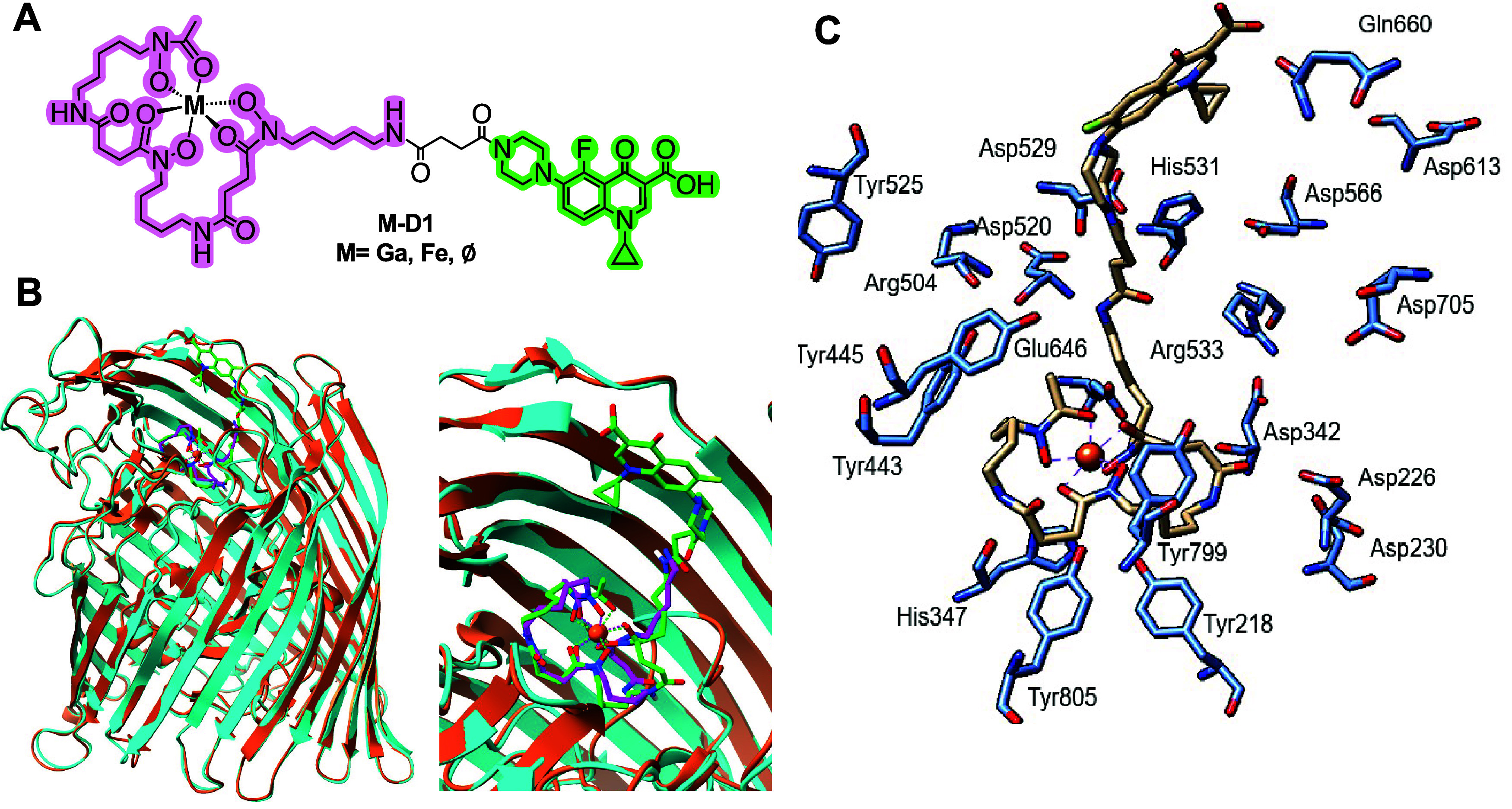
(A) Chemical structure of conjugate **M-D1**, with the desferrioxamine moiety highlighted in magenta and the ciprofloxacine moiety highlighted in green. (B) Cocrystal structure overlays of Fe-DFO (magenta) bound to FoxA (copper) and **Fe-D1** (green) bound to FoxA (turquoise) of the holo protein and substrate binding region (right) demonstrate that Fe-DFO conjugates replicate the binding mode of the corresponding Fe-bound siderophore with high accuracy. (C) Analysis of polar side chain amino acids involved in binding and interaction with Fe-D1 (tan) indicates a prevalence of tyrosine and aspartic acid within the binding pocket, in addition to other polar amino acids (shown in blue).

**Figure 2:**
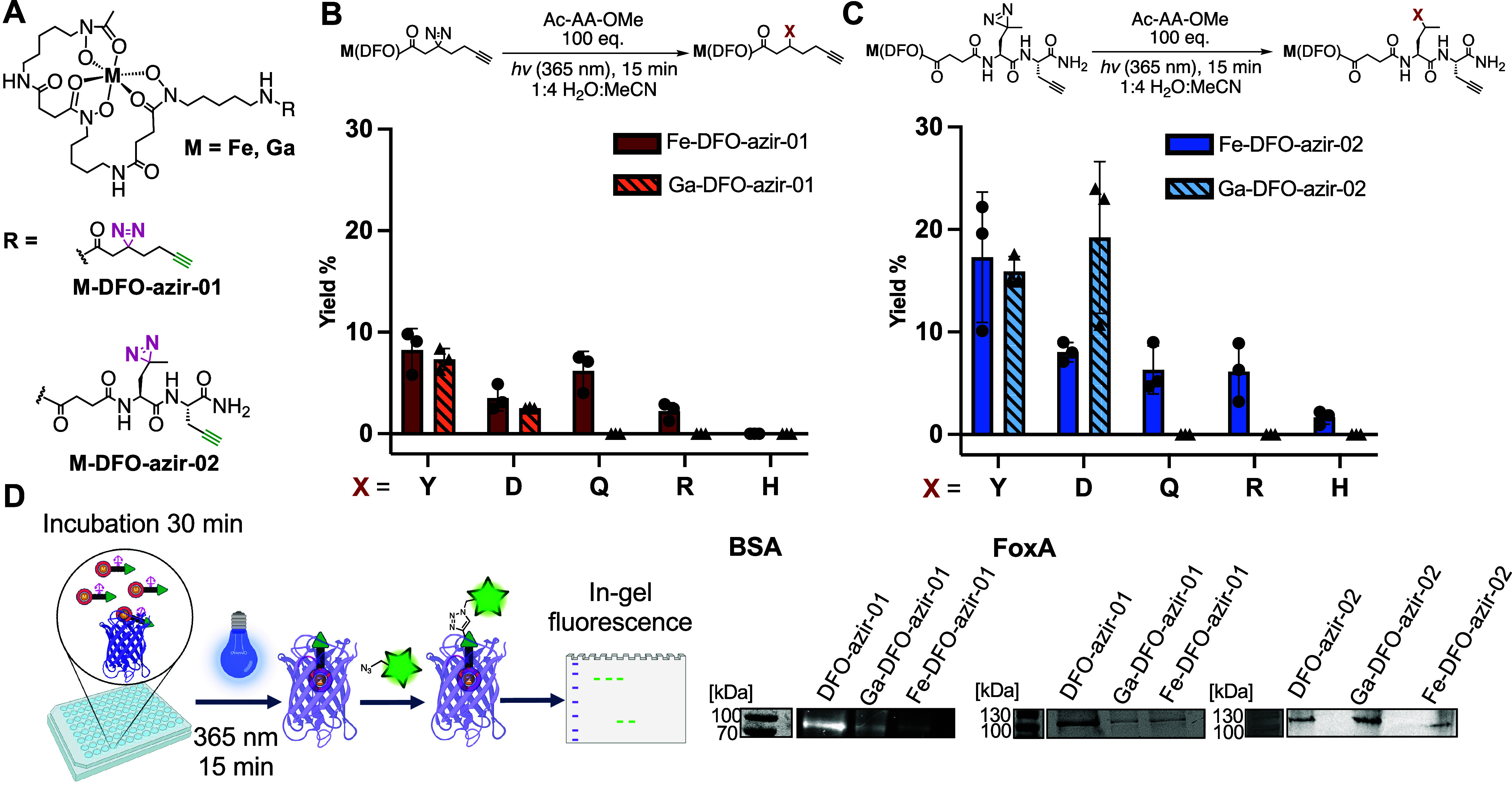
M-DFO-azir-01 and 02 reactivity with 5 natural amino acids. (A) Structure of **M-DFO-azir-01** and **02**. (B) Diazirine reactivity of **M-DFO-azir-01** with N- acetyl, O-Me protected amino acids (100 equiv) in 1:4 water acetonitrile. Yield was calculated by LC-MS following UV-absorbance at 425 nm for the Fe complex and 280 nm for the Ga complexes. (C) Diazirine reactivity of **M-DFO-azir-02** with N- acetyl, O-Me protected amino acids (100 equiv) in 1:4 water acetonitrile. Yield was calculated by LC-MS following UV-absorbance at 425 nm for Fe complex and 280 nm for the Ga complexes. (D) Evaluation of the ability of **M-DFO-azir-01** and **02** to label BSA and FoxA *in vitro*. Probes were incubated with purified protein 30 min followed by a 15 min irradiation under UV light (365 nm). Analysis was conducted by visualizing in-gel fluorescence on SDS-PAGE. Relative fluorescence intensity was observed in comparison to total protein staining. The full gel and corresponding total protein stain are provided in Figure S56.

Recent work by Woo^17–19^ and others^20,21^ has demonstrated how photochemically activatable, covalent cross-linking strategies of pharmacophores can identify unknown biological targets. While this strategy works especially well for shallow binding sites on cytoplasmic protein targets, the implementation of this approach to metal-bound siderophores could provide unprecedented access to direct identification of thus far unknown siderophore transport mechanisms.

An impediment to implementation is presented by the characteristics of siderophore transporters: membrane-associated barrel proteins with deep-seated binding sites are challenging to tag.^22–24^ As a possible consequence, covalent siderophore pulldown probes have remained absent from the literature to date. Recently, the first covalent metal complex tags, clickable heme-diazirines, have provided insight into bacterial and mammalian transport and metabolism of this essential metal-containing cofactor.^25,26^ This provides motivation to pursue an analogous approach for the identification of siderophore-binding proteins.

Herein, we synthesized and evaluated the reactivity profile of diazirine-linked desferrioxamine (DFO) in complex with Fe and Ga, to select optimally reactive constructs for the selective tagging of target proteins from protein isolates and live-cell culture. Validation was conducted using computational, *in vitro*, and *in cellulo* approaches with the*Pseudomonas aeruginosa*outer-membrane protein FoxA, a known ferrioxamine E outer-membrane transporter. Lastly, we validate our lead probe by directly tagging and identifying DFO-binding proteins in the wild-type strain of*P. aeruginosa* PAO1 and *Escherichia coli*K-12.

## Results and Discussion

### Rational Design and Reactivity Profiling of DFO-Diazirine Probes

The transmembrane siderophore transport machinery of most bacterial organisms provides entry to a vast range of siderophore-linked molecules. This provides the foundation for the Trojan horse siderophore-antibiotic drug strategy, which has been successfully employed to enhance the growth inhibitory activity of antibiotics.^27–31^ We hypothesized that crystallographic elucidation of the binding mode of a siderophore-antibiotic conjugate could identify suitable loci for the incorporation of a covalent diazirine tag. To this end, we obtained and examined the cocrystal structure of **Fe-D1**,^28^ a Fe-DFO complex linked to ciprofloxacin, with the previously characterized,*P. aeruginosa*outer-membrane siderophore binder FoxA (Figure 1). Comparison with previously obtained structural data with Fe-DFO bound to FoxA^32^ indicates that the functionalized siderophore retains the chelate’s binding mode and site. Although no density could be obtained for the ciprofloxacin moiety, likely due to high flexibility, the position of the linker could be determined, localizing in the channel leading to the siderophore-binding locus inside the barrel and above the plug domain. Its localization provided information about possible covalent tagging sites. The substrate binding site and channel are lined with tyrosine (*e.g* Tyr 218, 443, 445, 525, 799, 805), aspartic acid (*e.g* Asp 220, 226, 342, 520, 529, 566, 613, 705), and glutamine (*e.g* Gln 660) residues as suitable reactive partners to diazirine-mediated photo-cross-linking (Figure 1C).

**Figure 3:**
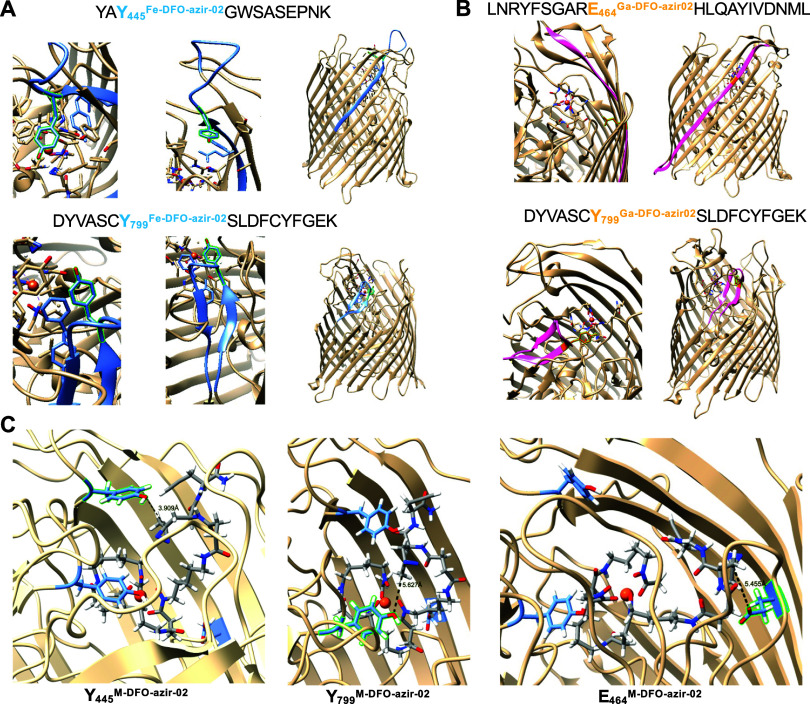
Photo-cross-linking of protein isolates and sites of conjugation. (A) An annotated FoxA protein fragment shows conjugation with **Fe-DFO-azir-02**. In the FoxA cocrystal structure with **Fe-DFO-azir-01**, amino acids labeled by **Fe-DFO-azir-02** are highlighted in green, and the protein fragment is shown in blue. (B) An annotated FoxA protein fragment shows conjugation with **Ga-DFO-azir-02**. In the FoxA cocrystal structure with **Fe-DFO**, amino acids labeled by **Ga-DFO-azir-02** are shown in orange, and the protein fragment is shown in pink. (C) Computational results show that **M-DFO-azir-02** can be recognized by FoxA and cross-link Y445, Y799, and E646 (highlighted in green). The distances (in Ångströms) between the carbon atom linked to the diazirine and the side chains of amino acids labeled by **Fe-DFO-azir-02** and **Ga-DFO-azir-02** are shown in the FoxA structure.

Based on the reactivity profile of diazirines obtained by Woo and co-workers,^17^ we reasoned that the combination of an aliphatic diazirine with longer-wavelength irradiation (365 nm) would be better suited to cross-link to tyrosine and aspartate side chains. While this design was based on the FoxA protein, we note that other, previously crystallographically characterized siderophore-binding proteins, such as FhuA in complex with albomycin,^33^ FhuA in complex with rifamycin^34^ and FhuD in complex with coprogen, demonstrate conservation of a high density of tyrosines and aspartates.^35^

The crystallographic analysis of **Fe-D1** bound to FoxA also provided information on potential modifications to the conjugate structure that a siderophore transmembrane transporter could tolerate. Specifically, we sought to incorporate the diazirine and alkyne into the linker moiety and vary the bond length between the DFO NH_2_ terminus and the diazirine reactive group. The diazirine-alkyne handle was synthesized in one step, followed by conjugation with either *apo*-DFO or DFO complexed with Fe^3+^ or Ga^3+^ to yield the *apo*-conjugate **DFO-azir-01** or metalated **M-DFO-azir-01**, employing a minimal design where the diazirine alkyne is directly coupled to the amine using a solution-phase conjugation strategy (Scheme S1). A second derivative, **DFO-azir-02**, incorporated a succinate linker between the siderophore amine terminus in direct homology with D1: the diazirine and alkyne functionalities were introduced as single amino acids. Accordingly, **DFO-azir-02** was synthesized via solid-phase peptide synthesis (SPS) (Scheme S2). Metal complexation with Fe^3+^ and Ga^3+^ with DFO was carried out prior to its direct incorporation into the peptide during solid-phase synthesis. Both Ga­(III) and Fe­(III) complexes exhibited stability throughout the resin cleavage process to afford the desired metalated probes **Ga-DFO-azir-02** and **Fe-DFO-azir-02**.

To investigate the reactivity of the two alkyne DFO-diazirines with amino acids, we conducted an amino acid side chain reactivity screen using Ac-X-O-Me amino acids (X = Tyr, Asp, Gln, Arg, His) with photoirradiation at 365 nm to favor reaction with tyrosines. The conditions and amino acids were chosen in accordance with accessible side chains within the FoxA binding pocket (Tyr, Asp) and incorporating other polar, diazirine-reactive amino acids as reactivity controls (His, Arg, Gln). **Ga**- and **Fe-DFO-azir-01** were anticipated to recapitulate reactivity trends reported with similar aliphatic alkyl diazirines. While the two complexes exhibited similar reactivity and comparable yields with Tyr and Asp (>5%), we also observed covalent modification of Gln, Arg, and His (2–5%), with the Fe­(III) complex showing greater reactivity with the latter group when compared to the Ga­(III) complex. The linker extended derivatives **Ga-** and **Fe-DFO-azir-02** showed comparable trends, with the Fe complex also retaining greater reactivity toward the second amino acid group (Figure 2B). Overall, our findings affirmed that both designs for our DFO-diazirine probes should retain satisfactory reactivity with Tyr and Asp, residues that are well-represented within the FoxA binding pocket.

**Figure 4:**
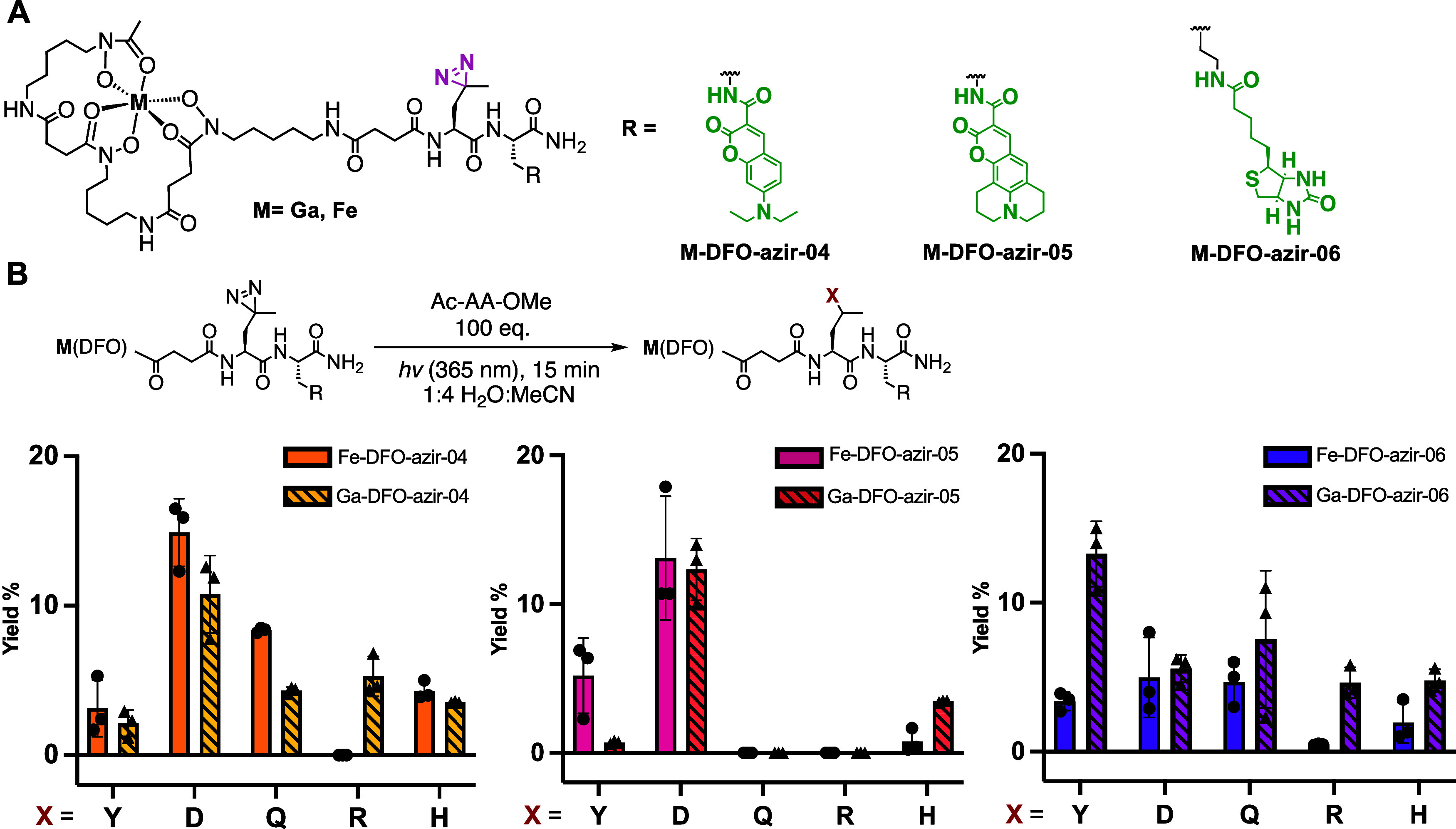
(A) Structure of **M-DFO-azir-04**, **-05**, and **-06**. (B) Diazirine reactivity of **M-DFO-azir-04**, **-05**, and **-06** with N-acetyl, O-Me protected amino acids (100 equiv) in 1:4 water acetonitrile. Yield was calculated by LC-MS following UV-absorbance at 425 nm for Fe complexes and 280 nm for Ga complexes.

### Covalent Tagging of BSA and FoxA

Next, we probed the compatibility of **Ga-** and **Fe-DFO-azir-01/02** with the tagging and subsequent copper-catalyzed conjugation (CuAAC) of a fluorophore with a large model protein. We first targeted nonspecific conjugation to bovine serum albumin (BSA). To this end, samples were incubated with **M-DFO-azir-01** and **02** (5 μM) at 37 °C for 30 min, followed by 15 min of photoirradiation at 365 nm. Subsequently, the labeled products were visualized by conjugation with a fluorophore, azide-fluor 545, by CuAAC and in-gel fluorescence (Figure 2D). Upon successful tagging and visualization of BSA, we then employed our labeling strategy for the tagging of isolated FoxA *(* Figure 2D*)*. For **Fe** and **Ga-DFO-azir-01**, we observed probe-dependent fluorescence, albeit with weak fluorescent bands and limited evidence of significant FoxA protein tagging. Moreover, CuAAC compatibility with DFO-diazirine probes appeared to be suboptimal, as coupling did not occur at room temperature, contrary to the starting material’s reactivity in the absence of proteins. Heating the reaction mixture at 45 °C was necessary to achieve conjugation with azide-fluor 545, possibly due to temperature-induced conformation changes of the β-barrel. Additionally, we were unable to detect **M-DFO-azir-02** probe-dependent fluorescence. We first posited that the presence of the metal ion could have a quenching effect; indeed, Fe^3+^ complexes are well-known to induce nonradiative quenching through photoinduced electron transfer.^36,37^ Nevertheless, this should not be the case with Ga^3+^, yet we observed poor conversion to the desired product with all 4 coordination complexes tested.

**Figure 5:**
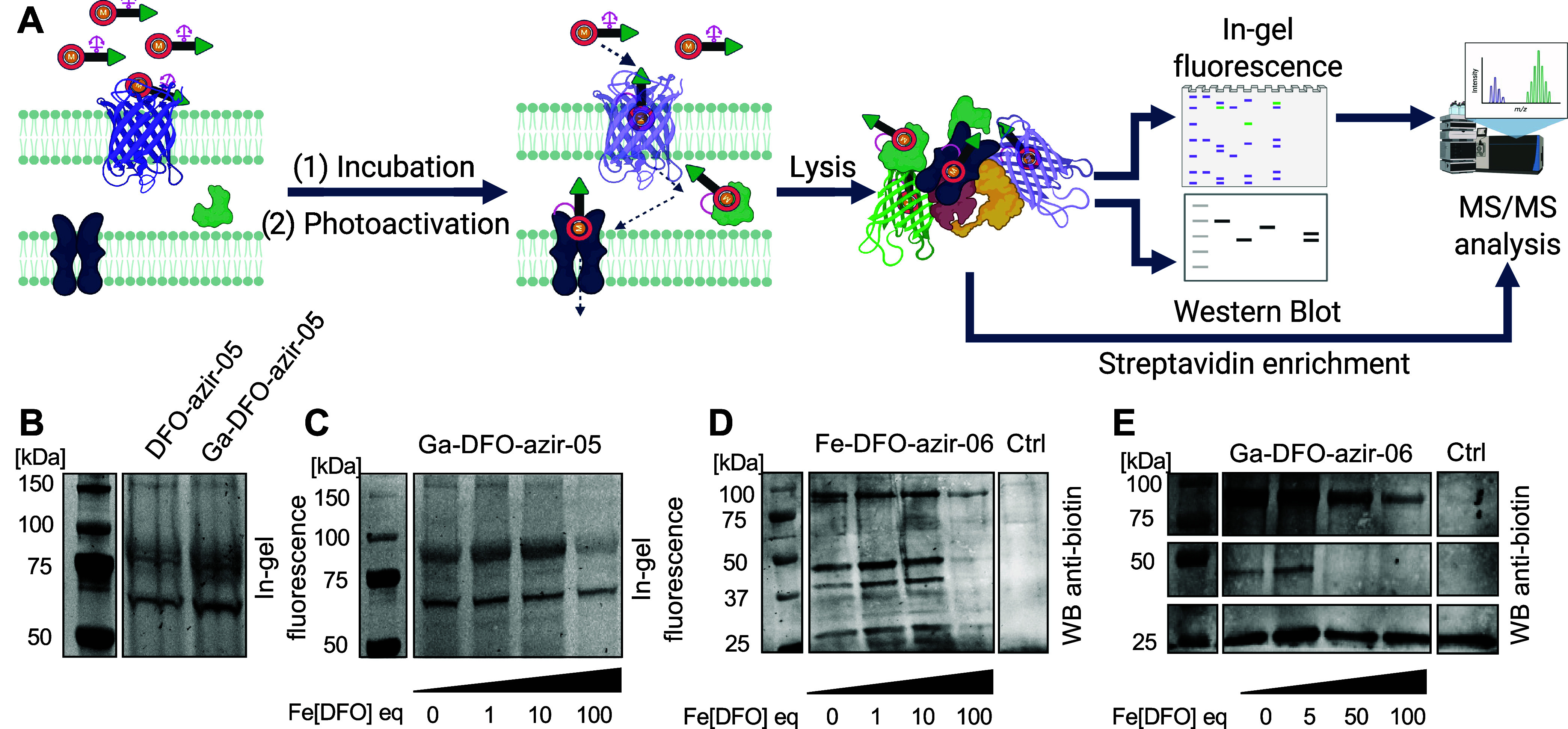
Validation of **M-DFO-azir-05** and **-06** in*E. coli*Lemo21 (DE3) cells overexpressing *FoxA*. (A) Workflow: combination of in-gel fluorescence and antibiotin Western blot to visualize proteins and conduct competition assays. Further analysis of in-gel digestion and streptavidin enrichment confirmed protein identification. (B) In-gel fluorescence of probes **DFO-azir-05** and **Ga-DFO-azir-05**. (C) Competition assay on **Ga-DFO-azir-05** using increasing concentrations of Fe-DFO. The decrease in band intensity of specific Fe-DFO-binding proteins, correlated with the increase of Fe-DFO, is observed by in-gel fluorescence. (D) Competition assay on **Fe-DFO-azir-06** using increasing concentrations of Fe-DFO. The decrease in band intensity of specific Fe-DFO-binding proteins, correlated with the increase of Fe-DFO, is observed by Western blot using antibiotin antibodies. The Ctrl band corresponds to untreated (without probe or Fe-DFO) cells. (E) Competition assay on **Ga-DFO-azir-06** using increasing concentrations of Fe-DFO. The decrease in band intensity of specific Fe-DFO-binding proteins, correlated with the increase of Fe-DFO, is observed by Western blot using antibiotin antibodies. The Ctrl band corresponds to untreated (without probe or Fe-DFO) cells. Complete gel images, along with total protein staining used to determine band intensity, can be found in Figures S58–S59.

We sought to further investigate the specificity of the DFO-diazirine probes. As covalent labeling probes, it is possible to directly identify sites of covalent photo-cross-linking using MS/MS analysis. Thus, we conducted MS/MS analysis of the photo-cross-linking and clicked reaction product with FoxA. We did not detect any peptide fragments that contained a covalently linked DFO with a triazole-linked fluorophore, which was in accordance with our observations that the reaction products exhibited poor fluorescence. However, MS/MS analysis revealed peptide fragments that were covalently modified with the probes in the alkyne form. Peptide fragments identified indicated that **Fe-DFO-azir-01** reacted preferentially with an aspartic acid (D502) located in a β-sheet within the top of the barrel (Figure S63), whereas **Fe-DFO-azir-02** and **Ga-DFO-azir-02** labeled residues situated within the protein binding pocket. Specifically, **Fe-DFO-azir-02** labeled two tyrosine residues (Y445 and Y799) (Figure 3A), and **Ga-DFO-azir-02** labeled a tyrosine residue (Y799) as well as a glutamic acid (E464) (Figure 3B). We note that while **Fe** and **Ga-DFO-azir-01** and **02** appear intact in MS/MS analysis, iron may dissociate during analysis, and the free chelator can subsequently rechelate trace iron present in solvents and instrumentation. The differences in the two probes’ tagging profiles within FoxA highlight the importance of the linker length to selectively tag amino acids in the immediate vicinity of the Fe-DFO-binding site. Indeed, crystallographic characterization of **Fe-DFO-azir-01** following photo-cross-linking attempts shows no indication of covalent tagging within the target binding site (Figure S50), whereas **M-DFO-azir-02** labels tyrosine residues in the immediate vicinity of the substrate binding site (Figure 3A,B). The crucial role of linker length was further affirmed as both constructs exhibited comparable reactivity with carboxylic acid and phenol side chains (Figure 2B) during the reactivity screen.

**Figure 6:**
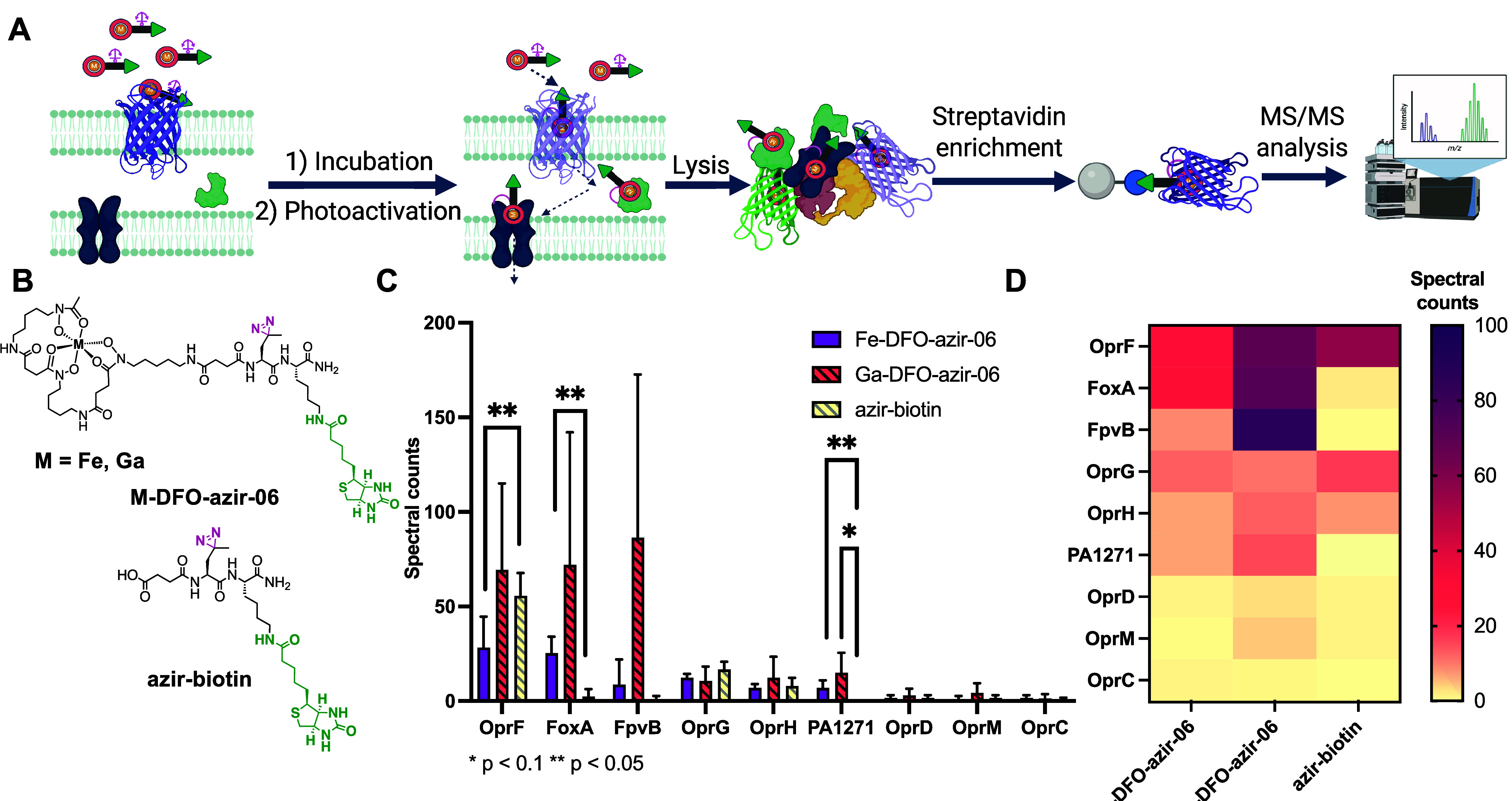
Ga and Fe-DFO-azir-06 enrichment in*P. aeruginosa*PAO1 cells. (A) Enrichment protocol: incubation of **M-DFO-azir-06** probes or control **azir-biotin**, photoconjugation to interacting proteins, followed by biotin–streptavidin enrichment and MS/MS analysis. (B) Structure of **M-DFO-azir-06** probes and control **azir-biotin**. (C) Quantification by spectral counts of transporters identified by MS/MS in enrichment assays using **M-DFO-azir-06** and **azir-biotin**. (D) Heatmap representation of spectral counts of transporters identified by MS/MS in enrichment assays using **M-DFO-azir-06** and **azir-biotin**. The results shown are representative of three independent experiments (Table S2).

### Computational Modeling of Covalently Tagged FoxA

Based on our promising cross-linking results for **M-DFO-azir-02**, we sought to verify that these linkages indicate that cross-linking occurs while the Fe-DFO complex is bound to the target binding site. In the absence of successful crystallographic characterization of the photo-cross-linked product, we conducted computational studies using the crystal structure of FoxA with a model of **M-DFO-azir-02**. The position of the Fe-DFO moiety was fixed within the binding pocket, and simulations were run to observe the flexibility of the diazirine-alkyne tail. Computational results show that when **M-DFO-azir-02** sits within the Fe-DFO-binding pocket, the diazirine is within a < 6 Å proximity of Y445, Y799, as well as E464 **(** Figures 3C and S51). This further supports that **M-DFO-azir-02** is efficiently recognized by FoxA. Based on these computational models, we also hypothesized that the alkyne group may not be sufficiently accessible within the barrel protein, hindering fluorophore conjugation. This is consistent with the low yields observed with the CuAAC of **M-DFO-azir-02** to azide-fluor 545. To address this issue, we synthesized **M-DFO-azir-03** (Scheme S3), incorporating a PEG_2_ linker. However, MS/MS analysis on **M-DFO-azir-03** revealed a loss of selectivity for the target binding site (Figure S66) entirely, eliminating the PEGylation strategy as a viable solution to the cross-linking problem.

### Design and Reactivity Profiling of DFO-Diazirines with Prelinked Optical and Pulldown Tags

To circumvent the need for postphoto-cross-linking conjugation with DFO-diazirine probes, we directly incorporated an optical tag and biotin into the DFO-diazirine scaffold: **M-DFO-azir-04, −05**, and -**06** were synthesized by SPS (Figure 4A, Schemes S4–S6).

These probes retained the same linker length and diazirine position as **M-DFO-azir-02** but incorporated the coumarin fluorophores 7-(diethylamino)­coumarin-3-carboxylic acid (*E*_x_ = 415 nm, *E*_m_ = 465 nm) and coumarin 343 (*E*_x_ = 444 nm, *E*_m_ = 480 nm), respectively, conjugated through a diaminobutanoic acid linker. **M-DFO-azir-06** incorporated an extended lysine linker attached to biotin, enabling the identification of the labeled proteins of interest through biotin–streptavidin enrichment. The relative fluorescent quantum yields of **Ga-DFO-azir-04** and **Ga-DFO-azir-05** were comparable to those of the parent fluorophore. In contrast, both **Fe-DFO-azir-04** and **05** exhibited significant fluorescence quenching induced by the Fe-DFO complex, likely due to photoinduced electron transfer, resulting in relative quantum yields approaching zero (Figures S43–49).

The second-generation probes exhibited comparable reactivity trends to the first-generation conjugates; however, the aromatic, fluorophore-linked probes **M-DFO-azir-04** and **05** exhibited diminished reactivity with tyrosine. **M-DFO-azir-04** and **05** demonstrated a preference for aspartic acid (15–12% and 13–12% yield, respectively) instead. In contrast, **M-DFO-azir-06** exhibited a higher reactivity with tyrosine than with aspartic acid, particularly **Ga-DFO-azir-06** (∼13% yield).

The **Fe-DFO-azir-06 probe** (3% yield) displayed lower reactivity compared to its Ga counterpart (Figure 4). Presumably, metalated compounds exhibit increased reactivity due to the capping of polar hydroxamate functional groups that can interfere with intermolecular diazirine-induced cross-linking. However, this amino acid residue preference appears not unique to the DFO-diazirine probes: the control probe **azir-biotin** (Scheme S7), which lacks the DFO moiety, also exhibited a preference for tyrosine, aspartic acid, and glutamic acid (Figure S67).

**Figure 7:**
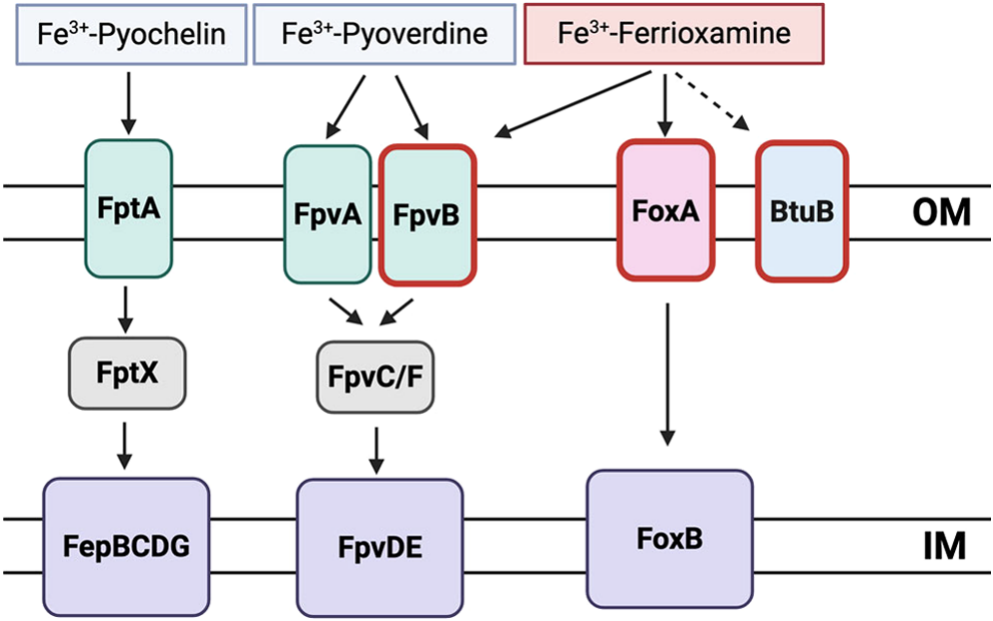
Schematic representation of DFO uptake pathways identified in*P. aeruginosa*using enrichment experiments with **M-DFO-azir-06** (highlighted in red). Probable TonB-dependent receptor PA1271, named BtuB due to its homology with*E. coli*cobalamine transporter BtuB, is identified by our experiments as a putative DFO transporter.

### Validation of M-DFO-Azir-05 and M-DFO-Azir-06 in Live-Cell Experiments

To validate our second-generation probes in live bacteria, we employed a preliminary model using*E. coli*Lemo21 (DE3) mutant strains transformed to overexpress FoxA. Cells were incubated with **M-DFO-azir-05** and **06** (25 μM) at 37 °C for 30 min, followed by photoirradiation. Subsequently, the bacterial cells were lysed, and the extent of labeling was visualized by in-gel fluorescence or antibiotin immunoblotting. To correctly identify proteins selectively tagged in live cells, it is important to determine the extent to which the observed fluorescence bands arise from siderophore-specific binding events. The pretagged optical and pulldown probes used in this study can interact with proteins either via siderophore-binding or through interactions with the fluorophore or biotin tag. To distinguish between siderophore-dependent and independent interactions, **M-DFO-azir-05** and **06** were incubated in the presence of increasing concentrations of the parent siderophore complex Fe-DFO.

Increasing concentrations of Fe-DFO suppressed the tagging of several proteins, which is consistent with the competitive binding of Fe-DFO. Specifically, an Fe-DFO-dependent decrease of intensity was observed for putative FoxA bands around 150 and 100 kDa with **Ga-DFO-azir-05** (Figure 5C) as well as **Fe-DFO-azir-06** and **Ga-DFO-azir-06** (Figure 5D,E). Additionally, the tagging of other unidentified proteins at 50 and 37 kDa was affected by the competitive binding of Fe-DFO (Figure 5D,E). In contrast, several bands showed no change in intensity in the presence of excess Fe-DFO, suggesting that those proteins were covalently tagged by nonspecific or biotin-derived interactions. MS/MS analysis of fluorescence bands obtained by incubation with **DFO-azir-05** indicated that relevant bands at 150 and 100 kDa correlate with FoxA, while bands at 50 and 37 kDa correlate with tryptophanase TnaA and the outer-membrane porin OmpF, respectively. Other protein bands that were not sensitive to excess Fe-DFO correlate with high abundance proteins such as elongation factor Tu-1 (TufA), and chaperone protein DnaK, and thus appear due to their natural high abundance within the bacterial cell (Figures S71–S76).

Next, we sought to determine if **M-DFO-azir-06** was fit to perform pulldown experiments, which involve a scale-up of the photo-cross-linking reaction, enrichment on streptavidin resin, and isolation of specific bands for subsequent MS/MS analysis. Enrichment experiments were conducted by incubating*E. coli*Lemo21 (DE3) mutants, transformed to overexpress FoxA, with **M-DFO-azir-06** (125 μM) at 37 °C for 4 h, followed by 15 min of photoirradiation. Then, cells were lysed, and proteins labeled by **M-DFO-azir-06** were isolated using streptavidin resin, followed by SDS-PAGE separation, excision, and analysis of relevant bands by MS/MS. The results reveal that FoxA and OmpF were tagged successfully (Figure S77), supporting the use of **M-DFO-azir-06** to tag and isolate membrane transporters by streptavidin enrichment.

### Direct Identification of DFO-Conjugate Specific Transporters in*P. aeruginosa*and*E. coli*

Upon validation in our preliminary model, we sought to test if it was possible to identify siderophore transporters that recognize and transport DFO conjugates in the wild-type*P. aeruginosa*strain, PAO1. Previously, our group has demonstrated that Ga-DFO–D1 showed growth-inhibitory activity in PAO1.^28^ While FoxA has been identified as the transporter for ferrioxamine E, other outer-membrane proteins have been implicated in the transport of ferrioxamine B and its derivatives.^38^

**Figure 8:**
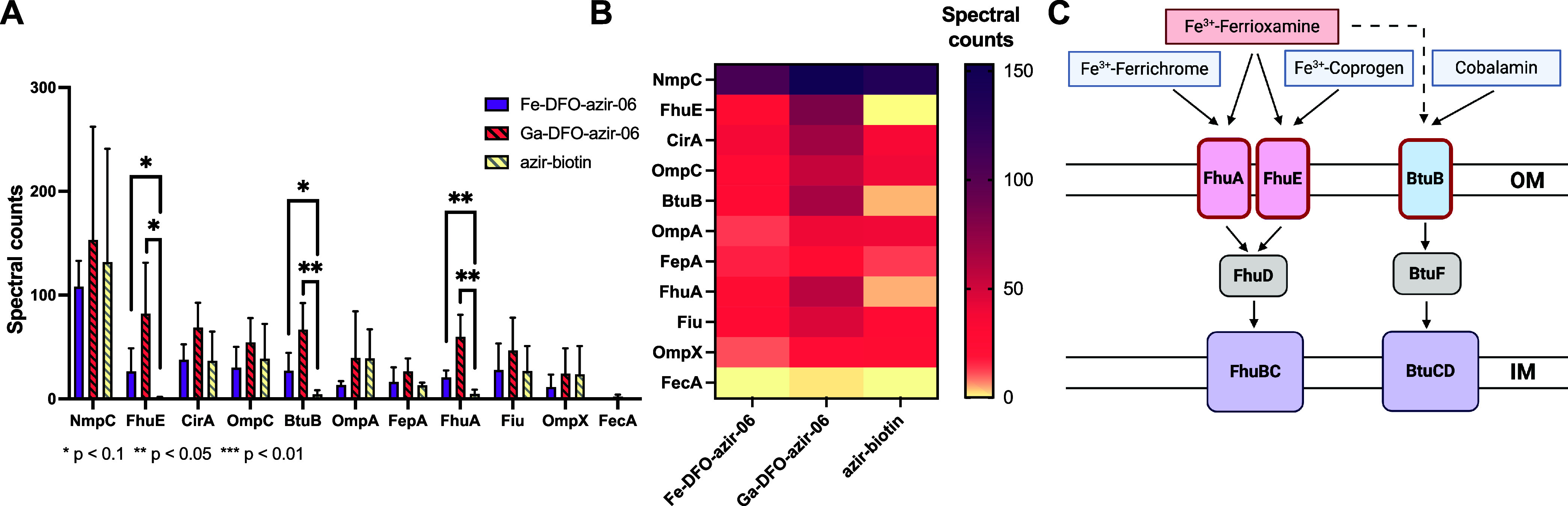
Ga and Fe-DFO-azir-06 enrichment in *E. coli* K-12 cells. (A) Quantification by spectral counts of transporters identified by MS/MS in enrichment assays using **M-DFO-azir-06** and **azir-biotin**. (B) Heatmap representation of spectral counts of transporters identified by MS/MS in enrichment assays using **M-DFO-azir-06** and **azir-biotin**. (C) Schematic representation of DFO uptake pathways identified in*E. coli*using enrichment experiments with **M-DFO-azir-06** (highlighted in red). The results shown are representative of three independent experiments (Table S3).

To stimulate the expression of ferrioxamine transporters, PAO1 cells were grown in Fe-deficient LB broth, supplemented with 10 μM of desferrioxamine. Cells were then incubated with **M-DFO-azir-06** or the control **azir-biotin** probe (125 μM, Figure 6B) to subsequently capture and enrich proteins of interest. All enriched proteins were subsequently analyzed by MS/MS, and a comparison between proteins tagged with **M-DFO-azir-06** and **azir-biotin**, which lacks the DFO moiety, was conducted to differentiate siderophore-specific binding from nontarget-specific interactions via the biotin moiety (Table S2).

Enrichment experiments revealed the identification of numerous outer membranes and cytoplasmic proteins. Enrichment of cytoplasmic proteins supports the internalization of probes across the inner membrane, with abundant cytoplasmic proteins such as GroL and DnaK highly enriched. This correlates with efficient growth inhibition of*P. aeruginosa*by hydroxamate-based conjugates of ciprofloxacin, where the intact complex interacts and inhibits gyrase, a cytoplasmic target.^39^

Numerous transport proteins were also identified. High spectral counts of porins in all samples suggested partial probe internalization through nonspecific, porin-mediated uptake, possibly facilitated via the biotin moiety. In contrast, FoxA was significantly enriched in **Fe-DFO-azir-06** samples compared to **azir-biotin** (Figure 6C). Furthermore, the second ferric pyoverdine receptor FpvB was also preferentially enriched in **M-DFO-azir-06** samples, albeit with less significance (Figure 6C, Table S2). This is in good correlation with recent reports that FpvB can recognize and transport xenosiderophores ferrioxamine and ferrichrome.^40^ In this context, our results confirm that FpvB acts as a secondary transport pathway for DFO. In addition, a probable TonB-dependent transporter on gene PA1271 was found significantly enriched in **M-DFO-azir-06** samples. Although predicted as a putative cobalamin transporter by sequence alignment,^41,42^ our results suggest that PA1271 can recognize and transport DFO, revealing a new putative role of this transporter.

Next, we also sought to apply this approach to identify dominant transport mechanisms for DFO conjugates in*E. coli*. No specific ferrioxamine receptor has been identified yet in*E. coli*, although the ability of*E. coli*to recognize and selectively import DFO-linked antibiotics was demonstrated previously by us and others.^28,43,44^ Specifically, **Ga-D1** showed significant growth inhibitory activity (MIC_98_ = 1.9 μM) in*E. coli* K-12. Prior literature emphasizes the role of ferrichrome receptor FhuA in the internalization of linear hydroxamate-based siderophores such as albomycin. However, Ga-D1 retained its potency in*E. coli* AN193, an incompetent FhuA mutant, which indicated that a second outer-membrane transporter readily recognized DFO-linked metal complexes. Meanwhile, Grinter et al. demonstrated that*E. coli*ΔTBDT mutants solely relying on coprogen transporter FhuE for iron import showed only limited growth in the presence of DFO, also suggesting the involvement of a second outer-membrane transporter.^14^ Indeed, our enrichment experiments identified two dominant transporters. In addition to FhuA, we also show that FhuE is highly selective for DFO (Figure 8). This supports our hypothesis that a two-site-mediated uptake for DFO derivatives is indeed operational in *E. coli*. Furthermore, improved spectral counts and selectivity with the **Ga-DFO-azir-06** probe, compared to **Fe-DFO-azir-06**, also indicate that incubation with the Ga-complex can more efficiently retain Fe-deficient protein expression conditions.

The cobalamine transporter BtuB was also significantly enriched in **M-DFO-azir-06** samples, pointing to a role of this transporter in the internalization of DFO in*E. coli*. The identification of homologous transporters BtuB and PA1271 in two different Gram-negative bacteria strains (*E. coli*K-12 and*P. aeruginosa*PAO1, respectively) suggests that these transporters are more promiscuous in the internalization of metal complexes beyond cobalamin.

Overall, our results confirm that pretagged, photo-cross-linking labeling probes such as **M-DFO-azir-06** serve as efficient, covalent tags to directly isolate and identify siderophore transporters. Use of an adequate control probe lacking the siderophore moiety (**azir-biotin**) identifies off-target hits and enables the unambiguous identification of siderophore-specific proteins in the complex bacterial cell envelope. Our results also indicate that the use of a xenometal (Ga) chelated probe can further improve tagging fidelity, as the absence of Fe retains sustained expression of transport proteins of interest. Taken together, our results successfully recapitulate previously described DFO uptake pathways in *P. aeruginosa*, as well as give new, unprecedented insights into the internalization of DFO derivatives in*E. coli* (Figures 7 and 8).

## Conclusions

This study presents a systematic approach to the rational design, optimization, and validation of a first, functional siderophore-based photoaffinity probe incorporating the hydroxamate-based siderophore desferrioxamine. Leveraging structural insights from the cocrystal structure of the desferrioxamine-ciprofloxacin conjugate **Fe-D1** with the FoxA protein, we identified structural features ideal for the installation of the photoreactive diazirine functional group. Mass spectrometry analysis, supported by computational modeling, determined the critical role of the linker length in achieving effective labeling within the substrate binding pocket. The covalent labeling approach is advantageous for the direct identification of binding sites on isolated proteins compared to indirect approaches that require mutations on various protein components.

Acknowledging the challenges associated with CuAAC chemistry for the alkyne-functionalized probes, we explored a prelinked strategy in our second-generation probe design. This approach, while not as compact as the first-generation probe design incorporating click-reactive alkynes, offers a viable pathway to circumvent limitations posed by post-cross-linking conjugation to install fluorophores or affinity tags.^45,46^ Additionally, these prelinked conjugates efficiently recapitulate the conjugation mode and size of antibiotic-functionalized Trojan horse constructs, providing a more realistic model for conjugate internalization.

As prelinked pulldown probes produce (false positive) hits from off-target interactions through the fluorophore/biotin moiety, substrate challenge assays and quantitative proteomics using an adequate control lacking the siderophore component are required to identify target binding events. As such, we used **M-DFO-azir-06** in concert with control **azir-biotin** to identify siderophore-binding proteins in bacterial live-cell assays. We successfully identify and affirm the role of ferrioxamine receptor FoxA as well as the second ferric pyoverdine receptor FpvB in the recognition and transport of ferrioxamine-conjugates through the outer-membrane envelope of*P. aeruginosa*. Additionally, we successfully identified the two DFO-selective outer-membrane transporters FhuA and FhuE in*E. coli*. Furthermore, our results report an unprecedented putative role of cobalamine transporter BtuB in the uptake of DFO in*P. aeruginosa* and*E. coli*.

In conclusion, this work provides a blueprint for how siderophore photoaffinity probes are optimized, validated, and applied in live-cell experiments to isolate siderophore transporters. Future work will focus on expanding this strategy to other siderophores to enable the direct identification of siderophore-binding transmembrane proteins, providing unprecedented insight into the transport of endogenous and xenosiderophores in bacteria.

## Supplementary Material









## Notes

Syntheses protocols, characterization data for all compounds; additional experimental details, materials, and methods (PDF)
Fe-D1 cocrystal structure with FoxA refine 12-coot-0 (1) (PDB)
Fe-DFO-azir-01 cocrystal structure with FoxA refine 10-coot-3 (PDB)
Spectral counts and statistical analysis of enrichment experiments in PAO1 and ECK-12 (XLSX)

## ⊥

Athinoula A. Martinos Center for Biomedical Imaging, Institute for Innovation in Imaging (i3), Department of Radiology, Massachusetts General Hospital, Harvard Medical School, Boston, Massachusetts 02115, United States

## #

Newcastle University Biosciences Institute (NUBI), Catherine Cookson Building, Newcastle University, Newcastle upon Tyne NE1 7RU, United Kingdom

## Abbreviations

BSA: bovine serum albumin
CuAAC: copper-catalyzed azide–alkyne cyclization
DFO: desferrioxamine
SPS: solid-phase peptide synthesis.
