# Supplementary material for: Covalent Chemical Tagging of Transmembrane Transport Proteins Illuminates the Internalization Pathways of Xenosiderophores
Source: J Am Chem Soc. 2026 May 13;148(20):20509–19. doi: 10.1021/jacs.6c00632 (PMC13220308; doi:10.1021/jacs.6c00632)
Supplement: Supplementary file 1 [file ja6c00632_si_001.pdf]

Supporting information for:

Covalent Chemical Tagging of Transmembrane Transport Proteins Illuminates the Internalization Pathways of Xenosiderophores

Minhua Cao,[a]‡† Marie Huynh,[a]‡ Inokentijs Josts,[b,c]† Yichong Lao,[a] Hung H. Dang,[a] Axia Marlin,[a] Xuhui Huang,[a] Henning Tidow,[b,c] and Eszter Boros*[a]

[a]Department of Chemistry University of Wisconsin-Madison, 1101 University Avenue, Madison, Wisconsin 53705, USA *Email: eboros@wisc.edu

[b]The Hamburg Advanced Research Center for Bioorganic Chemistry, Hamburg 22761, Germany.

[c]Department of Chemistry, Institute for Biochemistry and Molecular Biology, University of Hamburg, Hamburg 22761, Germany

‡These authors contributed equally

Table of Contents

Abbreviations 3
1. Experimental procedures 4
  1.1 General material and methods 4
  1.2 Syntheses 5
  1.3 Characterization data and spectroscopy of ligands 13
    1.3.1 HRMS and HPLC trace 13
    1.3.2 NMR spectra 22
  1.4 Quantum yields 34
2. Co-crystal structures 36
3. Computational experiments 38
4. Amino acids reactivity screening 39
5. Biological assays 41
  5.1 General biological methods 41
  5.2 Protein covalent tagging 42
    5.2.1 FoxA purification 42
    5.2.2 BSA and FoxA covalent tagging 43
  5.3 Bacterial lysate and live cell labeling 44
  5.4 Enrichment experiments 45
    5.4.1 Enrichment in E. coli Lemo21 (DE3) cells 45
    5.4.2 Enrichment in P. aeruginosa PAO1 and E. coli K-12 cells 46
6. Tandem mass spectrometry analysis 47
  6.1 Material and methods 47
  6.2 FoxA labeling sites identification 50
  6.3 Analysis of in-gel fluorescence bands 54
  6.4 Analysis of enrichment experiments 57
References 59

Abbreviations

DFO	Desferrioxamine
MeCN	Acetonitrile
TCEP	Tris (2-carboxyethyl) phosphine
Ci	Curie
TFA	Trifluoroacetic acid
DMF	Dimethylformamide
DPBS	Dulbecco′s Phosphate Buffered Saline
DIPEA	Diisopropylethylamine
RT	Room temperature
O/N	Over night
HPLC	High performance liquid chromatography
LC-MS	Liquid chromatography–mass spectrometry
UV	Ultraviolet
EDTA	Ethylenediaminetetraacetic acid
LB	Luria-Bertani
OD	Optical density
HEPES	2-[4-(2-hydroxyethyl)piperazin-1-yl]ethanesulfonic acid
WT	Wild type
CPM	Counts per minute
BSA	Bovine serum albumin
DMSO	Dimethyl sulfoxide
MIC	Minimum inhibitory concentration
LB	Lysogeny broth/ Luria–Bertani medium
eV	Electronvolt
HRMS	High resolution mass spectrometry
MALDI	Matrix Assisted Laser Desorption/Ionization
SDS	Sodium dodecyl sulfate
SDS-PAGE	sodium dodecyl sulfate–polyacrylamide gel electrophoresis
TRIS	tris(hydroxymethyl)aminomethane

1. Experimental procedures

1.1 General material and methods

All starting materials were purchased from Sigma-Aldrich, Ambeed, Fisher, Iris Biotech GmbH, TCI America, or Thermofisher and used without further purification. The UV-light was obtained from Waveform (realUV™ LED Flood Light, 365nm, 20W).

Nuclear magnetic resonance

^{1}H and ^{13}C NMR spectra were collected on a 700 or 500 MHz AVANCE III Bruker instrument or a Bruker Advance 500 MHz (DCH cryoprobe) or 600 MHz (TCl-F cryoprobe) at 25 °C. Data were acquired under automation with iconNMR and processed with MestRenova. Chemical shifts are reported in parts per million (ppm).

Mass spectrometry

High-resolution mass spectrometry was performed on a Bruker Impact II QTOF or at the UW-Madison Chemistry Paul Bender Chemical Instrumentation Center Facility using a Thermo Q Exactive™ Plus (electrospray ionization-quadrupole-ion trap) mass spectrometer. Low-resolution LC-MS was conducted using an Agilent 1260 Infinity II LC system coupled with an Agilent InfinityLab LC/MSD system, equipped with a single quadrupole and spray ionization source. A Phenomenex Luna C18 column (250 mm × 21.2 mm, 100 Å, AXIA packed) was used. The mobile phase consisted of 0.1% formic acid in water (A) and 0.1% formic acid in acetonitrile (B), with a flow rate of 0.8 mL/min. UV detection was performed at 254 and 220 nm. The gradient method was as follows: 0-3 min, 5% B; 3-10 min, 5-95% B; 10-13 min, 95% B; 13-13.5 min, 95-5% B; 13.5-16 min, 5% B. MALDI measurements were carried out on a Bruker Microflex LRF MALDI TOF. Samples were prepared by concentrating the protein, dissolving it in 50% acetonitrile in water (containing 0.1% formic acid), and mixing with SA (sinapic acid) matrix in a 1:1 and 1:3 ratio for analysis.

Liquid chromatography

Preparative HPLC was performed using either a Shimadzu HPLC-20AR equipped with a binary gradient pump, UV-vis detector, and manual injector, or an Agilent 1260 Infinity II system equipped with a binary gradient pump, UV-vis detector, and manual injector. A Phenomenex Luna C18 column (250 mm × 19 mm, 100 Å, AXIA packed) was used. For preparative purification method B, the mobile phase consisted of 0.1% formic acid in water (A) and 0.1% formic acid in acetonitrile (B), with a flow rate of 15 mL/min. UV detection was performed at 190 and 220 nm for the ligand and Ga complex of **DFO-azir-01**, **-02**, **-03**, and **-06**, and at 280 and 425 nm for the Fe complex and **DFO-azir-04** and **-05** at 280 and 425 nm. The Analytical HPLC was performed using an Agilent 1260 Infinity II system equipped with a binary gradient pump, UV-vis detector, and autoinjector. A Phenomenex Luna C18 column (150 mm × 3 mm, 100 Å, AXIA packed) was used. For gradient method D, the mobile phase consisted of 0.1% formic acid in water (A) and 0.1% formic acid in acetonitrile (B), with a flow rate of 0.8 mL/min. UV detection was performed at 254 and 220 nm. The gradient was as follows: 0-5 min, 95% A; 5-24 min, 5-95% B.

Compound concentration determination

Concentrations of Ga and Fe metal complexes were determined using an Agilent 5110 ICP-OES. A seven-point standard calibration curve was generated for gallium and iron, with R^2 values of 0.999. Sample concentrations were determined based on this calibration curve. Ligand compound concentrations were determined through iron titration monitored by UV-Vis spectroscopy using the Fe[DFO] absorbance band

(ε = 2460 $cm^{-1}M^{-1}$ at 425 nm)[1] or by following the reported absorbance of the fluorophore for **M-DFO-azir-04** (ε = 34000 $cm^{-1}M^{-1}$ at 409 nm)[2], and **M-DFO-azir-05** (ε = 43000 $cm^{-1}M^{-1}$ at 445 nm)[3].

Bacterial culture OD_{600}

Bacterial culture OD600 was determined using a BioTek Epoch 2 microplate reader and Gen5 data analysis software.

Imager

Gel fluorescent imaging was obtained using either an iBright™ FL1500 Imaging System or a Typhoon 9400 Variable Mode Imager.

1.2 Syntheses

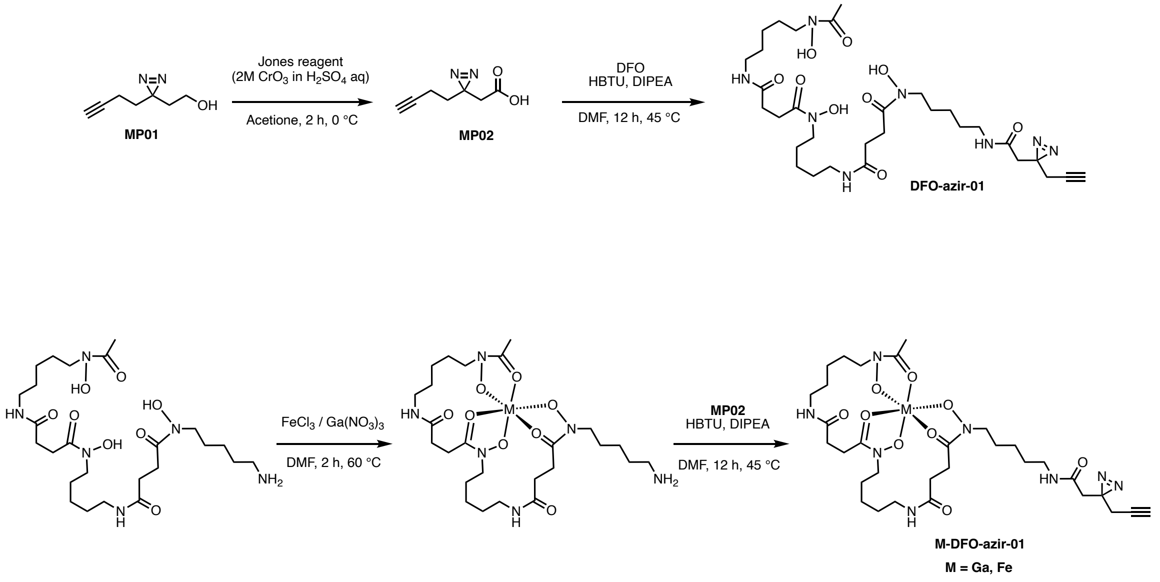

Scheme S1. Synthetic scheme of **M-DFO-azir-01.**

MP02 - *2-(3-(But-3-yn-1-yl)-3H-diazirin-3-yl) acetic acid*: Jones reagent (1 mL, 2 M CrO_3 in aqueous H_2SO_4) was added dropwise to a stirred solution of 2-(3-(but-3-yn-1-yl)-3H-diazirin-3-yl)ethanol (50.0 mg, 0.365 mmol, 1.00 eq) in acetone (5 mL) at 0 °C. The reaction mixture was stirred at room temperature for 2 h and then quenched with isopropanol (3 mL). The mixture was filtered through filter paper,[4] dried with Na_2SO_4, and evaporated to dryness. The crude product was used without further purification. Calculated mass ($C_7H_8N_2O_2$): 152.15; found 151.0 $[M-H]^-$.

DFO-azir-01-N1-(5-(2-(3-(but-3-yn-1-yl)-3H-diazirin-3-yl)acetamido)pentyl)-N1-hydroxy-N4-(5-(N-hydroxy-4-((5-(N-hydroxyacetamido)pentyl)amino)-4-oxobutanamido)pentyl) succinimide: N,N-Diisopropylethylamine (DIPEA, 4 eq, 1.46 mmol, 254 µL) was added to a stirring solution of MP01 in dry dimethylformamide (DMF). The mixture was stirred for 10 min before adding benzotriazol-1-yloxytripyrrolidinophosphonium hexafluorophosphate (PyBOP, 1 eq, 0.365 mmol, 189.9 mg). After an additional 10 min, deferoxamine (DFO, 1 eq, 0.365 mmol, 204 mg) was added, and the reaction was stirred overnight at 45 °C. The reaction mixture was evaporated to dryness and purified by preparative HPLC (method B) to afford DFO-azir-01 in 6% yield. Calculated mass ($C_{32}H_{51}N_8O_9$): 694.40; found 695.41 $[M+H]^+$. Retention time (Method A): 7.23 min.

^{1}H *NMR* (700 MHz, DMSO-d6) δ 9.65-9.61 (H=l, 2H), 7.88 (H=k, 1H), 7.78,-7.77 (H=j, 2H), 3.46-3.44(H=i, 6H), 3.05- 2.97 (H=h, 6H), 2.82-2.81 (H=e, 1H), 2.77-2.54 (H=f, 4H), 2.27-2.21 (H=g, 4H , m), 2.19 (H=d, 2H), 1.97- 1.96 (H=c, 3H, S), 1.64-1.62 (H=m, 2H), 1.51-1.34 (H=b, 14H m), 1.23-1.18 (H=a, 6H m).

^{13}C *NMR* (500 MHz, DMSO-d6) δ 171.94-171.28 (C=j, 2C), 170.11 (C=l, 3C), 166.92, 157.81, 157.64, 82. 75 (C=o, 1C), 71.77 (C=o, 1C), 38.41 (C=h, 3C), 38.38 (C=g, 2C), 31.87 (C=b, 2C), 29.86 (C=f, 3C), 28.81, 28.65 (C=e, 2C), 27.55, 26.69, 26.02 (C= d, 1C), 25.96 (c=a, 1C), 23.40 (c=a, 1C).

M-DFO: DFO (60 mg, 0.108 mmol, 1 eq.) was dissolved in DMF (2 mL). In a separate vial, $Ga(NO_3)_3$ (41.42 mg, 0.162 mmol, 1.5 eq) or $FeCl_3$ (26.1 mg, 0.162 mmol, 1.5 eq) was dissolved in DMF followed by the addition of DIPEA (3 eq). The resulting solution was added to the DFO solution, and the mixture was stirred for 1 h at 60 °C and 1 h at room temperature. The reaction mixture was used without purification in the subsequent reaction.

M- DFO-azir-01: Compound MP02 (15 mg, 0.108 mmol, 1 eq) was dissolved in DMF (3 mL). DIPEA (75.25 µL, 0.432 mmol, 4 eq) was then added, and the mixture was stirred for 10 min. PyBOP (1 eq., 0.365 mmol, 189.9 mg) was subsequently added, followed by another 10 min of stirring. A solution of Fe-DFO or Ga-DFO (66.25 mg, 0.108 mmol, 1 eq) in dry DMF was then introduced, and the reaction mixture was stirred at 45 °C overnight. The solvent was removed under vacuum, and the product was purified by preparative HPLC (Method B) to yield M-DFO-azir-01 as a white solid (Ga complex) or reddish orange solid (Fe complex) in 13% yield.

Calculated mass **Fe-DFO-azir-01** ($C_{32}H_{51}FeN_8O_9$): 747.65; found 748.32 $[M+H]^+$. Retention time (Method A): 6.91 min. Caculated mass **Ga-DFO-azir-01** ($C_{32}H_{51}GaN_8O_9$):761.53; found 761.31 $[M+H]^+$. Retention time (Method A): 6.94 min.

^{1}H *NMR* (700 MHz, DMSO-d6) δ 7.89-7.87 (H=k, 1H m), 7.60-7.57 (H=j, 2H s), 3.88-3.41 (H=i, 6H m), 3.02-2.85 (H=h, 6H), 2.82-2.81 (H=e, 1H m), 2.66-2.34 (H=f, g), 2.20-2.17 (H=d, 2H s), 2.04-2.03 (H=c, 3H s), 1.73-1.60 (H=a, 6H m), 1.41-1.09 (H=b, 14H m).

^{13}C *NMR* (500 MHz, DMSO-d6) δ 174.53 (C=j, 2C), 170.52, 170.28 (C=t, 1C), 166.95, 166.93, 163.05, 162.55, 161.03 (C=l, 3l), 83.24 (C=o, 1C), 71.75 (C=n, 1C), 38.44 (C=h, 4C), 37.52 (C=g, 3C), 31.88 (C=m, 1C), 30.43 (C=b, 2C), 30.36 (C=f, 3C), 26.72 (C=e, 2C), 25.39 (C=c, 5C), 23.26, 21.99 (c=a, 1C).

M-DFO-azir-02,03,04,05, and 06 solid phase peptide synthesis: Probes M-DFO-azir-03, 04, 05, and 06 were synthesized on a 0.093 mmol scale using Rink Amide (RA) resin (100 mg, 0.62 mmol/g, 1 eq.). The RA resin was swollen in DCM (3 mL) and DMF (3 mL) for 1 min, repeated three times for each solvent. The first amino acid (153, 0.124 mmol, 2 eq) was loaded onto the resin using PyBOP (48.4 mg, 0.092 mmol, 1.5 eq) as a coupling reagent in the presence of DIPEA (43.2 µL, 0.248 mmol, 4 eq.). The first coupling reaction proceeded at room temperature for 16 hours. A standard capping step was performed using an acetic anhydride/pyridine mixture (3:2) for 30 min. The Fmoc group was subsequently removed by treating the resin with 20% piperidine in DMF (3 mL) for 20 min. The Mtt group was deprotected by washing with 50 mL of 1% TFA in DCM until the wash solution was colorless. Subsequent amino acids were coupled using the same procedure until the full sequences were assembled. Succinic anhydride was loaded onto the resin in the presence of DIPEA (43.2 µL, 0.248 mmol, 4 eq.) for 1 h at room temperature. The Fmoc group on the N-terminus was then deprotected, and DFO or M-DFO (0.0761 mmol, 2 eq) was coupled using PyBOP (48.4 mg, 0.092 mmol, 1.5 eq) as a coupling reagent in the presence of DIPEA (43.2 µL, 0.248 mmol, 4 eq.) for 16 h. The compound was cleaved from the resin by treating it with a mixture of TFA/TIS/H_2O (95%/2.5%/2.5%) for 1 h at room temperature. The product was precipitated in cold 1:1

hexane/diethyl ether, dissolved in water/acetonitrile, and purified by preparative HPLC (method B) to yield **M-DFO-azir-02** (21% yield), **M-DFO-azir-03** (41% yield), **M-DFO-azir-04** (21% yield), **M-DFO-azir-05** (18% yield), and **M-DFO-azir-06** (19% yield) (**Schemes S2-S6**).

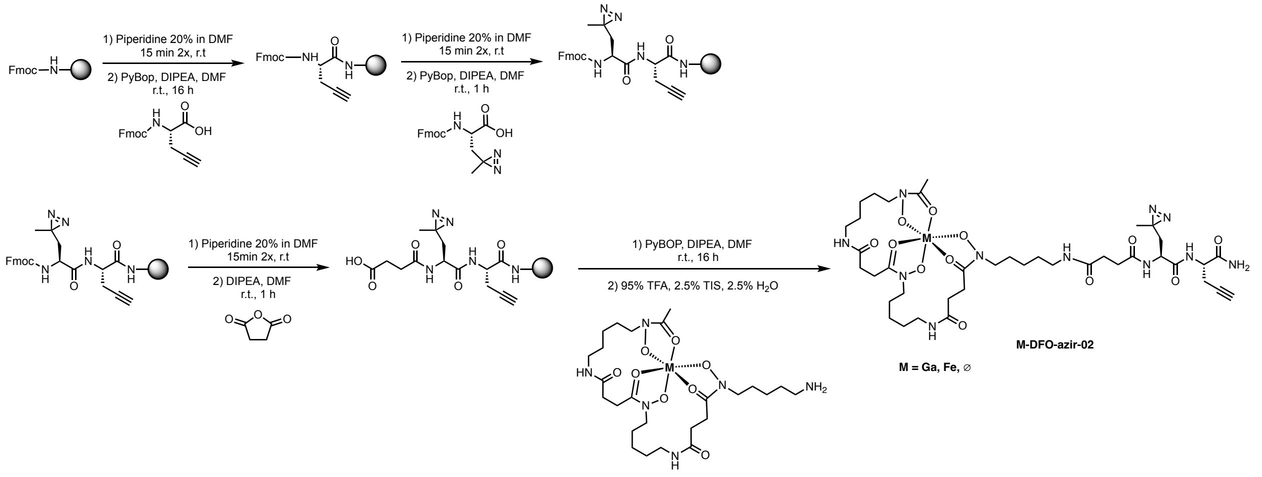

Scheme S2. Solid phase peptide synthesis of **M-DFO-azir-02.**

DFO-azir-02

Calculated mass ($C_{39}H_{65}N_{11}O_{12}$): 879.48; found 902.4706 $[M+Na]^+$, retention time (method A): 8.1min.

^{1}H NMR (500 MHz, DMSO-d6) δ 9.73 (H=l, 3H), 8.36-8.12 (H=k, 2H d), 7.93-7.80 (H= j, 3H m), 7.17-7.13 (H=n, 2H s), 4.24-4.02 (H=q, 2H m), 3.46- 3.44 (H=i, 6H), 3.00-2.86 (H=h,6H m), 2.8 (H=p, 1H),2.59-2.5 (H=f, 8H m), 2.38-2.36 (H= g, 6H m), 1.96 (H=c, 3H S), 1.96-1.88 (H=e, 2H) 1.51-1.36 (H=b, 12H m), 1.25-1.20 (H=a, 6H m), 1.01 (H=m, 3H s).

^{13}C NMR (500 MHz, DMSO-d6) δ 172.51 (C=w, 1C), 172.0 (C=j, 3C)2, 171.65-171.49 (C=r, 2C), 170.95 (C=l, 3C) , 80.90 (C=o, 1C), 72.87(C=p, 1C), 51.87(C=q, 2C), 49.28, 47.24, 46.93, 40.29 , 38.68, 38.56 (C=h, 4C), 36.00 (C=g, 3C), 31.05 (C=e, 4C), 28.95, 28.88 (C=f, 7C) , 27.76, 26.19 (C=d, 1C), 24.65, 23.71, 23.66 (C=a, 1C), 21.37, 20.51(C= K, 1C).

M-DFO-azir-02

Calculated mass **Fe-DFO-azir-02** ($C_{39}H_{62}FeN_{11}O_{12}$: 932.39; found 933.4001 $[M+H]^+$, retention time (method A): 6.78 min. Calculated mass **Ga-DFO-azir-02** ($C_{39}H_{62}GaN_{11}O_{12}$): 945.38; found 946.4001 $[M+H]^+$, retention time (method A): 7.61 min.

^{1}H NMR (500 MHz, DMSO-d6) δ 8.36-8.35 (H=k, 1H), 7.93-7.91 (H=j, 3H), 7.59-7.57 (H= j, 2H),7.18-7.11 (H=n, 2H d), 4.23-4.00 (H=q, 2H m), 3.58-3.41(H=i, 6H m),3.02-2.87(H=h, 6H m), 2.82- 2.81 (H=p, 1H), 2.63-2.52 (H=g, 6H t), 2.42- 2.13 (H=f, 8H m), 2.02 (H=c, 3H S), 1.91-1.88 (H=e, 2H d), 1.73- 1.06 (H=a , b, 20H m) 1.01(H=m, 3H s).

^{13}C NMR (500 MHz, DMSO-d6) δ 172.40 (C=j, 2C), 171.48 (C=w, 1C), 171.39-170.21 (C=r, 2) , 80.76 (C=o, 1C), 72.75 (C=p, 1C) , 51.72 (C=q, 2C), 38.58 (C=h, 6C), 35.80 (C=g, 2C), 30.88 (C=b, 2C), 30.59 (C=f, 3C), 28.78 (C=e, 6C), 26.57 (C= d, 1C), 24.50 (C=c, 7C), 21.20 (c=a, 1C), 19.65 (C=k, 1C).

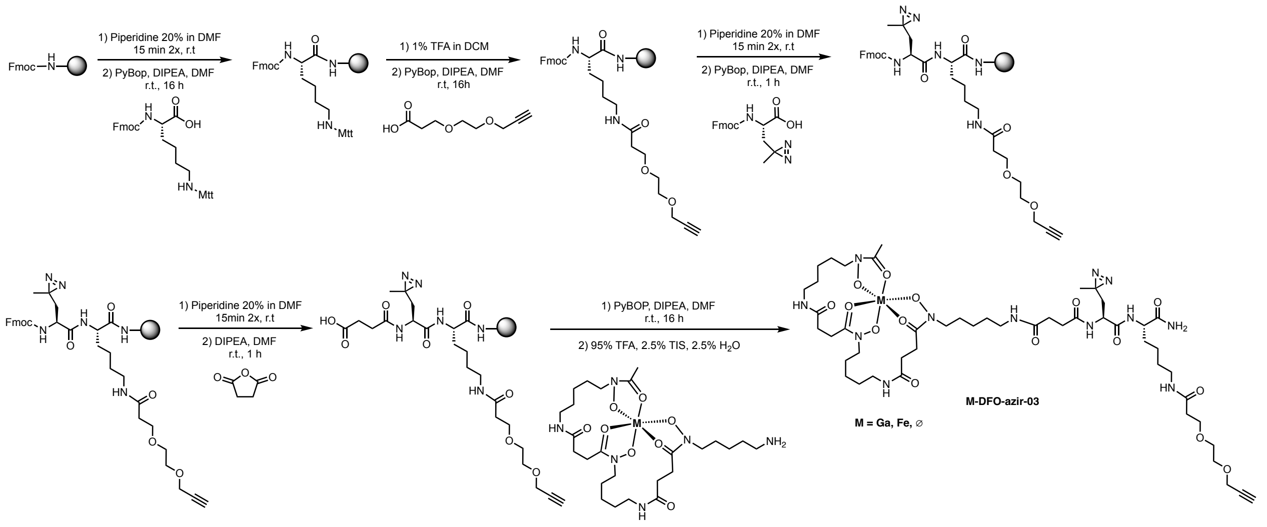

Scheme S3. Solid phase peptide synthesis of **M-DFO-azir-03**

DFO-azir-03

Calculated mass ($C_{48}H_{82}N_{12}O_{15}$): 1067.25; found 1067.6079 $[M+H]^+$, retention time (method A): 8.2 min.

^{1}H NMR (500 MHz, DMSO-d6) δ 9.64 (H=l, 3H), 8.29-8.27 (H=k, 2H m), 7.91-7.79 (H=j, 3H),7.03-6.98 (H=n, 2H m), 4.13-4.12 (H=s, 2H d), 4.07-4.01(H=q, 2H m), 3.59-3.48 (H=d, 6H m), 3.46-3.43 (H=i,6H m), 3.40-3.39 (H=p, 1H t), 3.01-2.97 (H=h, 8H m), 2.59-2.56 (H=g, 6H m), 2.42-2.25 (H=f, 8H m), 1.96 (H=c, 3H s), 1.92-1.89 (H=o, 2H), 1.73-1.64 (H=r, 2H m),1.54-1.31 (H=b, 14 H m, 1.24-1.18 (H=a, 8H m), 1.01 (H= m, 3H s).

^{13}C NMR (500 MHz, DMSO-d6) δ 173.54 (C=w, 1C), 172.24 (C=j, 4C), 171.96-171.39 (C=r, 2C), 170.69 (C=l, 3C) ,80.32 (C=t, 1C), 77.06 (C=s, 1C), 69.22 (C=o, 2C), 66.84 (C=n, 2C), 57.48 (C= p, 1C), 52.68 C=q, 2C), 49.08, 38.52 (C=h, 4C), 35.83 (C=g, 3C), 30.59 (C=e, 4C), 29.91(C=f, 7C) ,28.73 (C=m, 1C), 27.57 (C=c, 3C), 26.03(C=d, 1C), 23.50 (C=a, 1C), 20.34 (C= K, 1C).

M-DFO-azir-03

Calculated mass **Fe-DFO-azir-03** (C48H79FeN12O15: 1120.07; found 1120.5213 [M+H]+, retention time (method A): 8.10 min. Calculated mass **Ga-DFO-azir-03** ($C_{48}H_{79}GaN_{12}O_{15}$: 1133.95; found 1153.4929 $[M+Na]^+$, retention time (method A): 7.93 min.

^{1}H NMR (500 MHz, DMSO-d6) δ 8.50-8.33 (H=k, 2H), 7.97-7.95 (H=j, 3H), 7.79 (H=v, 1H), 7.03-6.98 (H=n, 2H m), 4.13 (H=s, 2H d), 4.05-4.03 (H=q, 2H m), 3.59-3.38 (H= i ,d, 13H m), 3.01-2.97 (H=h, 8H m), 2.74-2.54 (H=g, 6H m), 2.42- 2.27 (H=f, 8H m), 2.03 (H=c, 3H s), 1.92-1.88 (H=e, 2H m), 1.61-1.16 (H= a, b, r, 24H m), 1.01(H= m, 3H s).

^{13}C NMR (500 MHz, DMSO-d6) δ 173.69 (C=j, 3C), 172.41 (C=w, 1C), 171.53-170.36 (C=r, 2C), 165.83 (C=l,3C), 80.48(C=t, 1C), 77.23 (C=s, 1C), 69.37 (C=o, 2C), 67.00 (C=n, 2C), 57.64 (C= p, 1C), 49.26 (C=q, 2C), 38.74 (C=h, 5C), 35.98 (C=g, 3C), 31.28 (C=b, 2C), 31.09 (C=m, 1C), 30.77(C=f, 3C), 28.94 (C=e, 5C), 26.72 (c=d, 2C), 24.67 (C=c, 5C), 23.09 (c=a, 1C), 19.80 (C= K, 1C), 16.97.

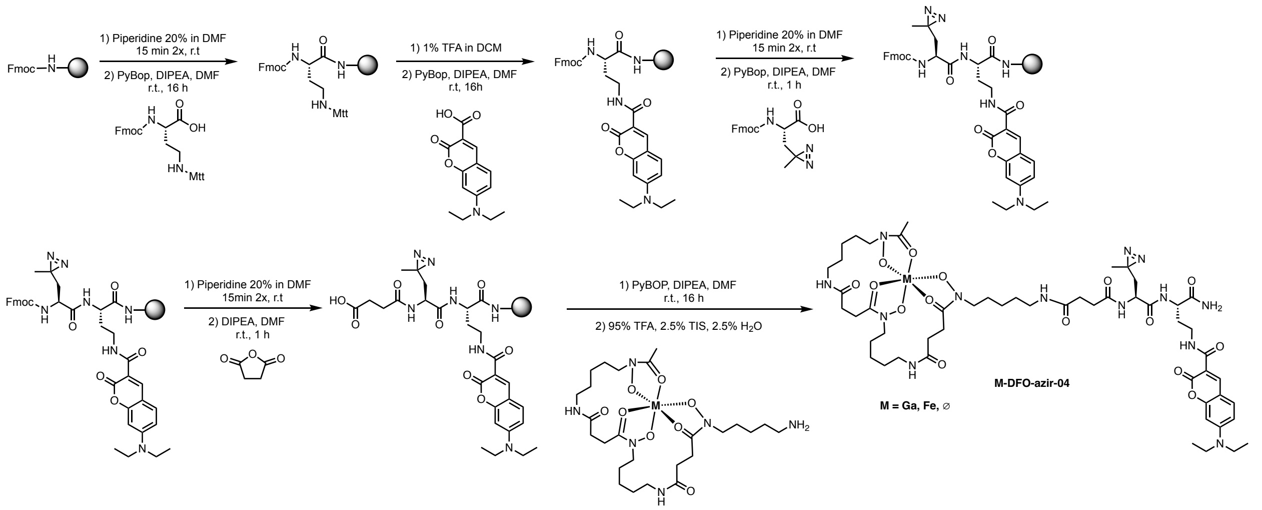

Scheme S4. Solid phase peptide synthesis of **M-DFO-azir-04.**

DFO-azir-04

Calculated mass ($C_{52}H_{81}N_{13}O_{15}$): 1128.30; found 1128.6060 $[M+H]^+$, retention time (method A): 9.31 min.

^{1}H NMR (500 MHz, DMSO-d6) δ 9.69-9.65 (H=l, 3H), 8.66-8.29 (H=k, 3H), 8.08-7.74 (H=j, 3H), 7.69-7.66 (H=u, 1H), 7.12-7.06 (H=n, 2H m), 6.81-6.79 (H=s, 1H d), 6.62- 6.61 (H=t, 1H s), 4.15-4.03 (H=q, 2H), 3.50-3.41 (H= r,i, 12H m) 3.01-2.97 (H=h, 6H m), 2.56-2.53 (H=f, 6H), 2.43-2.24 (H=g, 8H m), 1.94 (H=c, 3H s), 1.92-1.88 (H=e, 2H m),1.58-1.36 (H=b, 12 H m), 1.23-1.19 (H=a, 6H m), 1.15-1.12 (H=o, 6H t), 1.01(H=m, 3H s).

^{13}C NMR (500 MHz, DMSO-d6) δ 173.12 (C=w, 1C), 172.32 (C=j, 3C), 171.42 (C=r,1C), 170.80 (C=l, 3C), 162.27(C=i,C), 157.22 (C=p, 1C), 152.42 (C=v, 1C), 147.62 (C=z, 1C), 131.55 (C=s, 1C), 110.11 (C=o, 2C), 109.43 (C=t, 1C), 107.63, 95.87 (C=y, 1C), 50.80(C=q, 2C), 44.32 (C=x, 2C), 38.51 (C=h, 4C), 31.58(C=m, 1C), 30.97, 30.62(C=e, 4C), 28.80 (C=f, 7C), 28.71, 27.62(C=c, 3C), 26.03(C=d, 1C), 23.50 (C=a, 2C), 20.35(C= K, 1C), 12.3 (C=u, 2C).

Calculated mass **Fe-DFO-azir-04** ($C_{52}H_{78}FeN_{13}O_{15}$: 1181.12; found 1203.4964 $[M+Na]^+$, retention time (method A): 9.13min. Calculated mass **Ga-DFO-azir-04** ($C_{52}H_{78}GaN_{13}O_{15}$: 1195.00; found 1216.4890 $[M+Na]^+$, retention time (method A): 9.20min.

^{1}H NMR (500 MHz, DMSO-d6) δ 8.66-8.24 (H=k, 4H), 8.06-7.67 (H=j,3H), 7.57 (H=u, 1H), 7.11-7.06 (H=n, 2H d), 6.81-6.79 (H=s, 1H d), 6.62-6.61 (h=t, 1H s), 4.16-4.03 (H=q, 2H m), 3.57-3.46 (H=r, 6H m), 3.27-3.02 (H=i, 6H m), 3.00-2.82 (H=h, 6H m), 2.78-2.58 (H=g, 6H m), 2.45-2.13 (H=f, 8H m), 2.02 (H=c , 3H s), 1.95-1.91 (H=e, 2H m), 1.83-1.22 (H= a,b, 18H m), 1.15-1.11 (H=0, 6H t), 1.02 (H=m, 3H s)

^{13}C NMR (500 MHz, DMSO-d6) δ 173.08 (C=j, 2C), 172.34 (C=w, 1C), 171.41(C=v, 1C), 170.18 (C=r, 2C), 162.25 (C=i,C), 159.87 (C=l,3 C), 157.21 (C=v, 1C), 147.62 (C=z, 1C), 131.56 (C=s, 1C), 110.11 (C=o, 2C), 109.42 (C=t, 1C), 95.85 (C=y, 1C), 62.33 (C=n, 1C), 50.78 (C=q, 2C), 38.58 (C=h, 4C), 37.04, 35.74 (C=g, 2C), 31.58 (C=b, 2C), 30.96 (C=m, 1C), 30.61 (C=f, 3C), 28.75 (C=e, 6C), 26.55 (c=d, 2C), 24.52 (C=c, 6C), 21.50 (c=a, 1C), 19.63 (C= k, 1C), 12.30 (C=u, 2C).

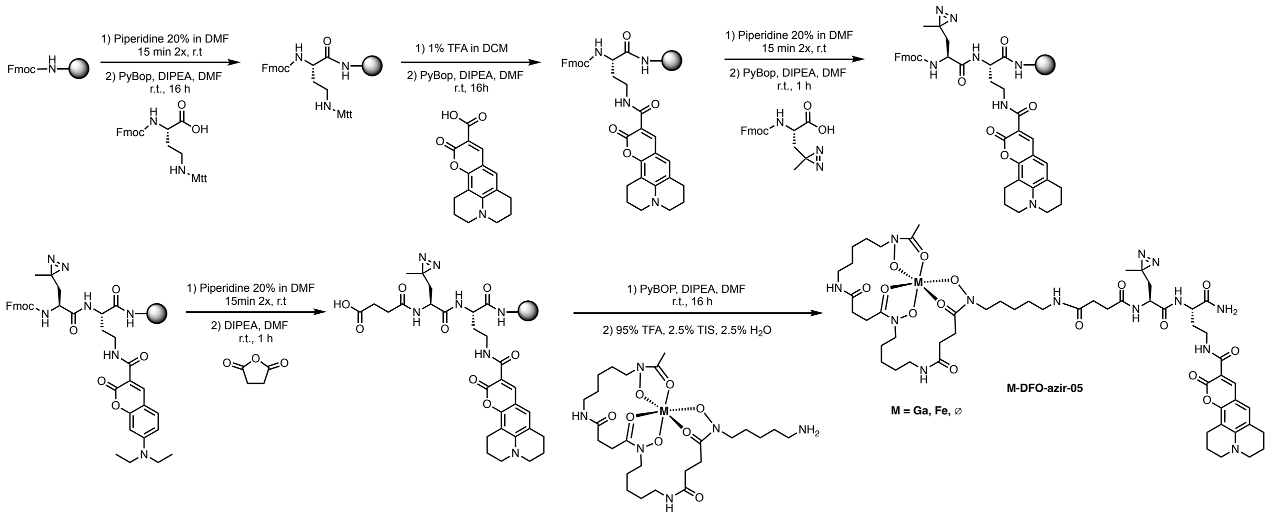

Scheme S5. Solid phase peptide synthesis of **M-DFO-azir-05.**

DFO-azir-05

Calculated mass ($C_{54}H_{81}N_{13}O_{15}$): 1152.32; found 1174.5872 $[M+Na]^+$, retention time (method A): 9.31 min.

^1H NMR (500 MHz, DMSO-d6) δ 9.72 (H=l, 3H), 8.67-8.49 (H=k, 3H m), 8.07-7.75 (H=j, 3H m), 7.26 (H=p, 1H s), 7.20-7.05 (H=n, 2H d), 4.14-4.02 (H=q, 2H m), 3.46-3.41 (H=r, 4H m), 3.01-2.97 (H=i, 6H m), 2.96-2.70 (H=h, 8H m), 2.62-2.55 (H= s, 4H m), 2.43- 2.33 (H=g, 6H m), 2.27-2.07 (H=f, 6H m), 1.96 (H= c, 3H s), 1.91-1.75 (H=e, 8H m),1.53-1.33 (H=b, 12H m), 1.28- 1.17 (H=a, 8H m), 1.01 (H=m, 3H s).

^{13}C NMR (500 MHz, DMSO-d6) δ 173.60 (C=w, 1C), 172.82 (C=j, 3C), 171.90 (C=v, 1C), 171.78-171.24 (C=r, 1C), 170.59 (C=l,3C), 163.02 (C=j,1 C), 162.28 (C=i, 1C), 152.55 (C=u, 1C), 147.92 (C=y, 2C), 127.62(C=z, 2C), 119.90-119.09 (C=x, 1C),107.84 (C=o,1C), 51.30 (C=n,1C), 50.01 (C=q, 2C), 49.62 (C=p, 2C), 39.00 (C=h, 4C), 36.22 (C=g, 4C), 32.08 (C=m, 1C), 29.28 (C=e, 4C), 29.19 (C=f, 7C), 27.29(C=c, 3C), 26.51(C= d, 2C), 22.56 (C=a, 1C), 21.02 (C=t, 2C), 20.08 (C= K, 1C).

M-DFO-azir-05

Calculated mass **Fe-DFO-azir-05** ($C_{54}H_{78}FeN_{13}O_{15}$: 1205.14; found 1205.5167 $[M+H]^+$, retention time (method A): 9.13 min. Calculated mass **Ga-DFO-azir-05** ($C_{54}H_{78}GaN_{13}O_{15}$: 1219.02; found 1240.4892 $[M+Na]^+$, retention time (method A): 9.12min.

^1H NMR (500 MHz, DMSO-d6) δ 8.68- 8.27(H=k, 3H), 8.03-7.66 (H=j, 3H m), 7.58(H=p, 1H s), 7.16-7.06 (H=n, 2H d), 4.40, 4.16-4.02 (H=q, 2H m),3.82-3.52 (H=r, 4H m), 3.51-3.44 (H=i, 6H m), 3.2-3.17 (H= h, 8H), 3.05- 2.98 (H=s, 4H m), 2.76-2.52(H=g, 6H m), 2.44-2.05 (H=f, 6H m), 2.02 ((H= c, 3H s), 1.91-1.76 (H=e, 8H m), 1.54-1.35 (H=b, 12H m), 1.26-1.17 (H=a, 8H m), 1.01 (H=m, 3H s).

^{13}C NMR (500 MHz, DMSO-d6) δ 173.10 (C=j, 2C), 172.35 (C=w, 1C), 171.41(C=r, 2C), 170.18 (C=v, 1C), 162.51 (C=i, 1C), 159.89 (C=l,4 C), 152.02 (C=u, 1C), 147.42 (C=y, 2C), 127.13 (C=z, 2C), 119.41 (C=x, 1C), 107.95 (C=o,1C), 63.06 (C=n,1C), 50.80 (C=q, 2C), 49.51 (C=p, 2C), 38.57 (C=h, 4C), 35.71(C=g, 3C), 31.58 (C=m, 1C), 31.26 (C=b, 2C), 30.60(C=f, 3C), 29.00 (C=s, 1C), 28.74(C=e, 5C), 26.54(C= d, 2C), 24.51(C=c, 6C), 23.20 (c=a, 1C), 20.52 (C=t, 2C),19.58 (C=k, 1C).

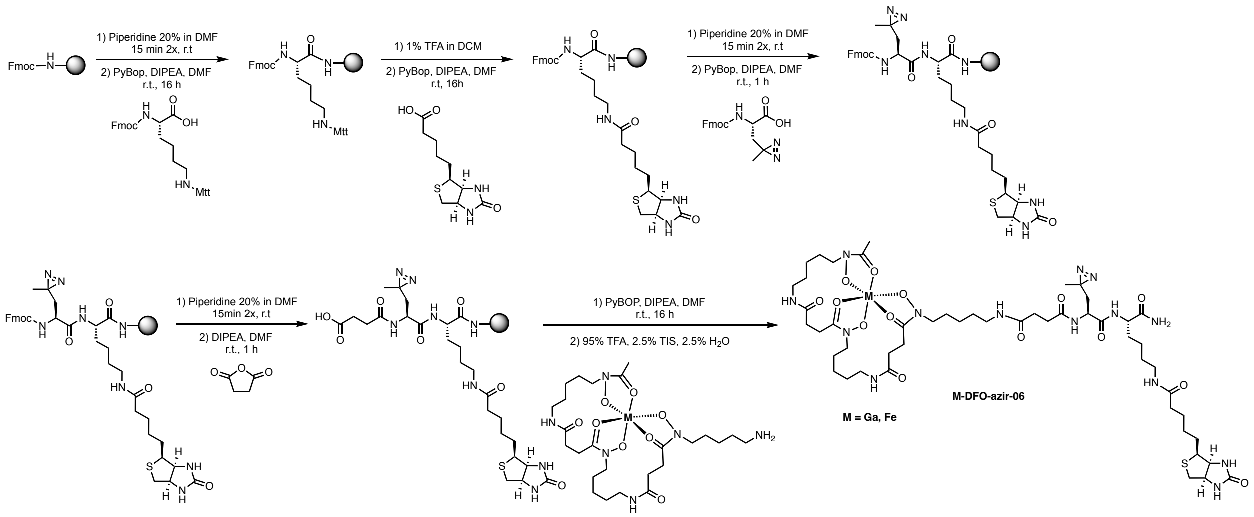

Scheme S6. Solid phase peptide synthesis of **M-DFO-azir-06.**

M-DFO-azir-06

Calculated mass **Fe-DFO-azir-06** ($C_{50}H_{83}FeN_{14}O_{14}S$: 1191.52; found 1214.5176 $[M+Na]^+$, retention time (method A): 7.66 min. **Ga-DFO-azir-06** Calculated mass ($C_{50}H_{83}GaN_{14}O_{14}S$: 1204.51; found 1227.5078 $[M+Na]^+$, retention time (method A): 7.59 min.

^{1}H NMR (500 MHz, DMSO-d6) δ 8.30-8.29 (H=k, 2H), 7.94-7.72(H=j, 3H), 7.02- 6.98 (H=n, 2H d), 6.41-6.35 (H=p, 2H), 4.32-4.29 (H=s, 1H m),4.14-4.11 (H=t, 1H m),4.07-4.01 (H= q, 2H m), 3.59-3.54 (H= o, 3H m), 3.10-2.80 (H= h,i, 14 H), 2.78-2.56 (H=g, 6H m), 2.42-2.33 (H=f, 6H m), 2.07-2.04 (H=d, 2H m) ,2.03 (H=c, 3H s), 1.92-1.88 (H=e, 2H m), 1.60-1.17 (H= a, b, r, 32H) , 1.00 (H= m, 3H s).

^{13}C NMR (500 MHz, DMSO-d6) δ 173.51 (C=j, 2C), 172.24 (C=w, 1C), 171.79(C=v, 2C),171.36-170.63 (C=r, 2C), 162.69 (C=p, 1C), 159.85 (C=l,3 C), 61.02(C=n,2C), 59.18 (C= u, 1C), 55.39 (C=s,1C), 52.70 (C=q, 2C), 38.56 (C=h, 6C), 35.78 (C=g, 2C), 35.17(C=t, 1C), 31.12 (C=b, 2C), 30.34 (C=f, 3C), 28.85 (C=e, 5C), 28.20(C= o, 1C), 28.00 (C=m, 1C), 26.54 (C= d, 3C), 26.29(C=c, 6C), 25.27 (C=x, 1C), 22.95 (c=a, 1C), 19.59 (C=k, 1C).

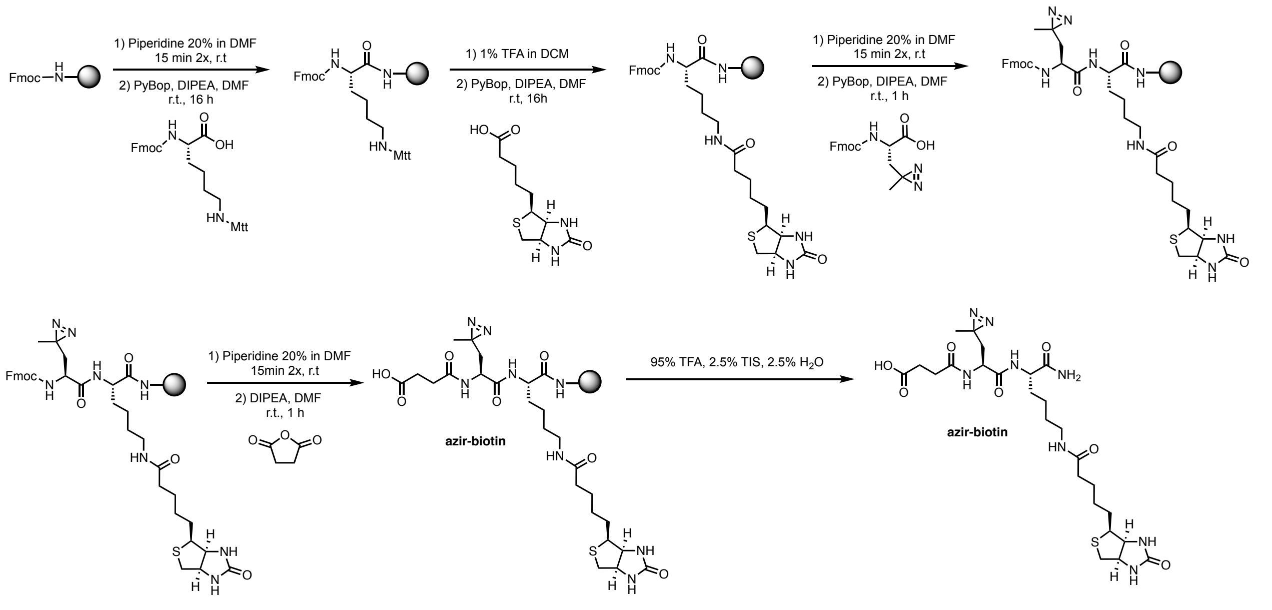

Scheme S7. Solid phase peptide synthesis of **azir-biotin.**

Azir-biotin

Calculated mass ($C_{25}H_{40}N_8O_7S$): 596.70; found 595.2663 $[M-H]^-$, retention time (method A): 6.99 min.

^{1}H NMR (500 MHz, DMSO-d6): δ 8.16-8.14 (H=k, 2H), 7.81-7.79 (H=C, 1H), 7.- 7.18(H=n, 2H),6.37-6.29 (H=p, 2H), 4.28-4.23 (H=s, 1H m), 4.17-4.0 (H= q, 3H m), 3.01-2.75 (H= o, 3H m), 2.43-2.33 (H=f, 4H m), 2.01-1.97 (H=d, 2H m) , 1.84-1.78 (H=e, 2H m), 1.50-1.13 (H= a, b, r, 10 H) , 1.00 (H= m, 3H s).

^{13}C NMR (500 MHz, DMSO-d6) δ 173.93 (C=c, 1C), 173.41(C=w, 1C), 171.83 (C=v, 1C), 171.41-170.53 (C=r, 2C), 162.71 (C=p, 1C), 129.64, 61.03 (C=n,1C), 59.19 (C= u, 1C), 55.39 (C=s,1C), 52.54 (C=q, 2C), 38.27 (C=h, 2C), 36.28, 35.19 (C=t, 1C), 28.86 (C=e, 3C), 28.71, 28.67, 28.54, 28.20 (C= o, 1C), 28.00 (C=m, 1C), 26.59-26.55 (C= d, 2C), 25.28 (C=x, 1C), 19.58 (C=k, 1C).

1.3 Characterization data and spectroscopy of ligands

1.3.1 HRMS and HPLC trace

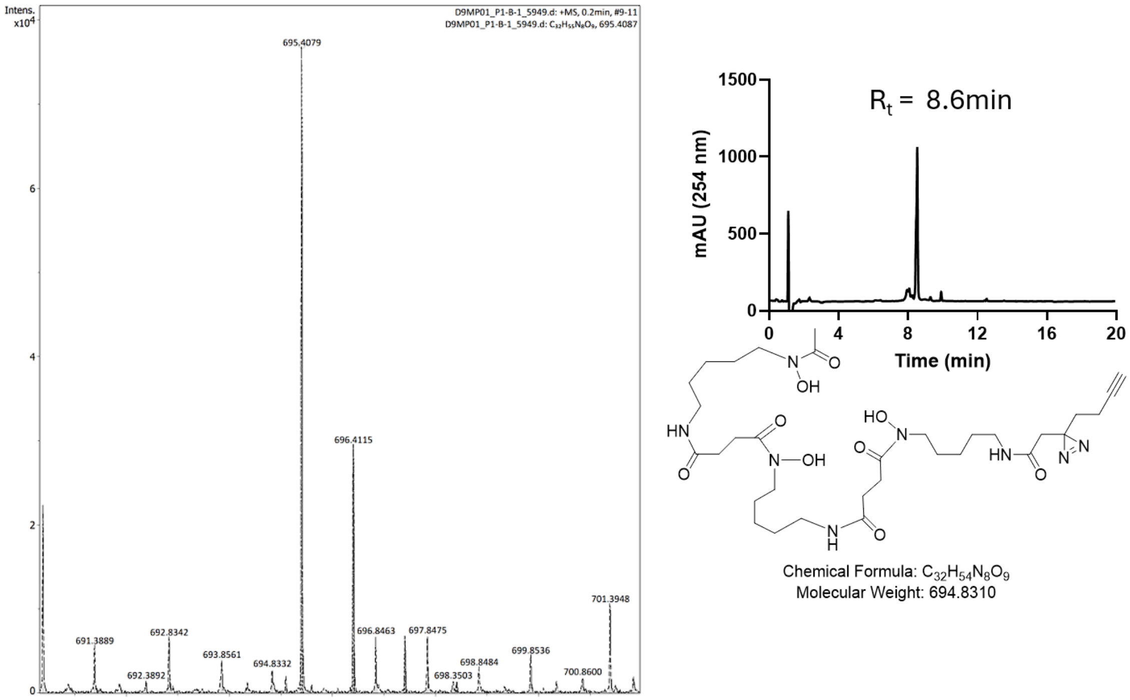

Figure S1. DFO-azir-01 HRMS and HPLC trace (Method D).

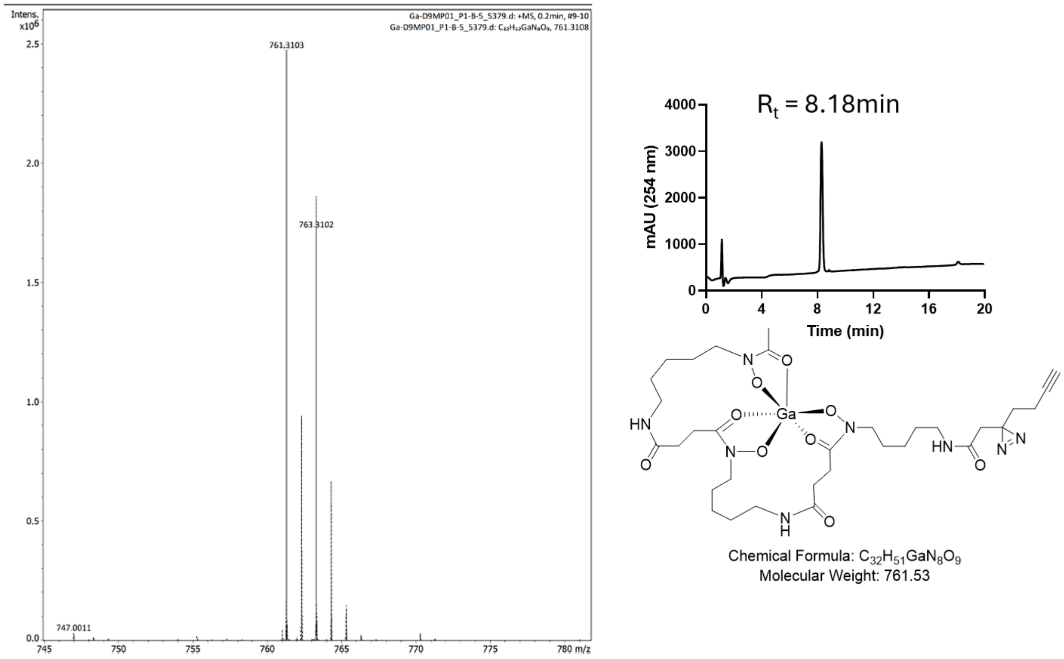

Figure S2. Ga-DFO-azir-01 HRMS and HPLC trace (Method D).

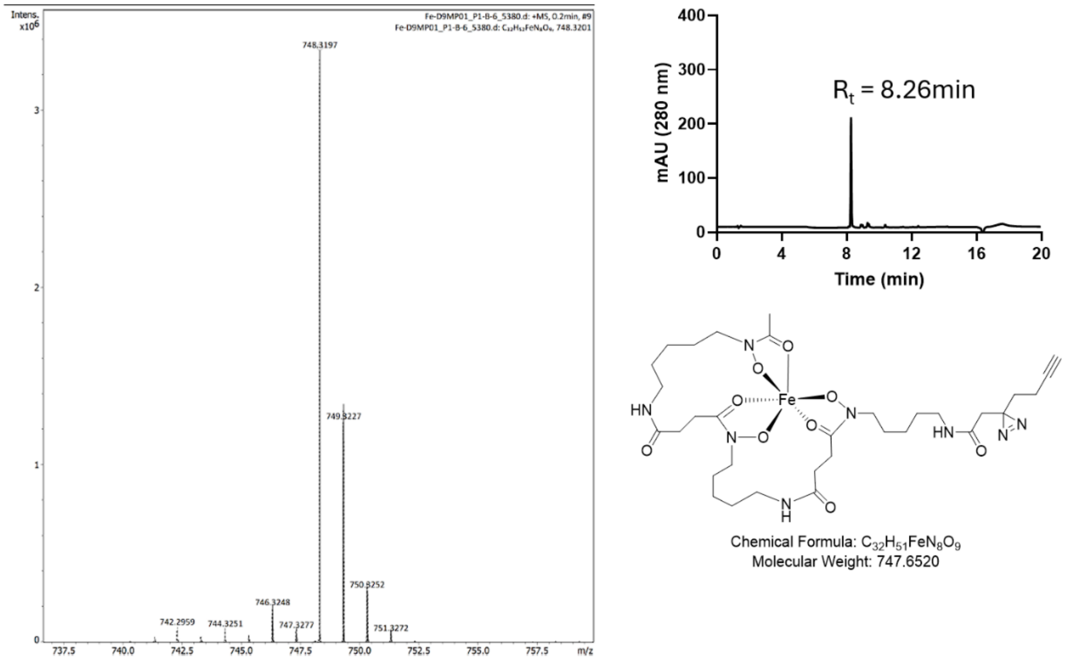

Figure S3. Fe-DFO-azir-01 HRMS and HPLC trace (Method D).

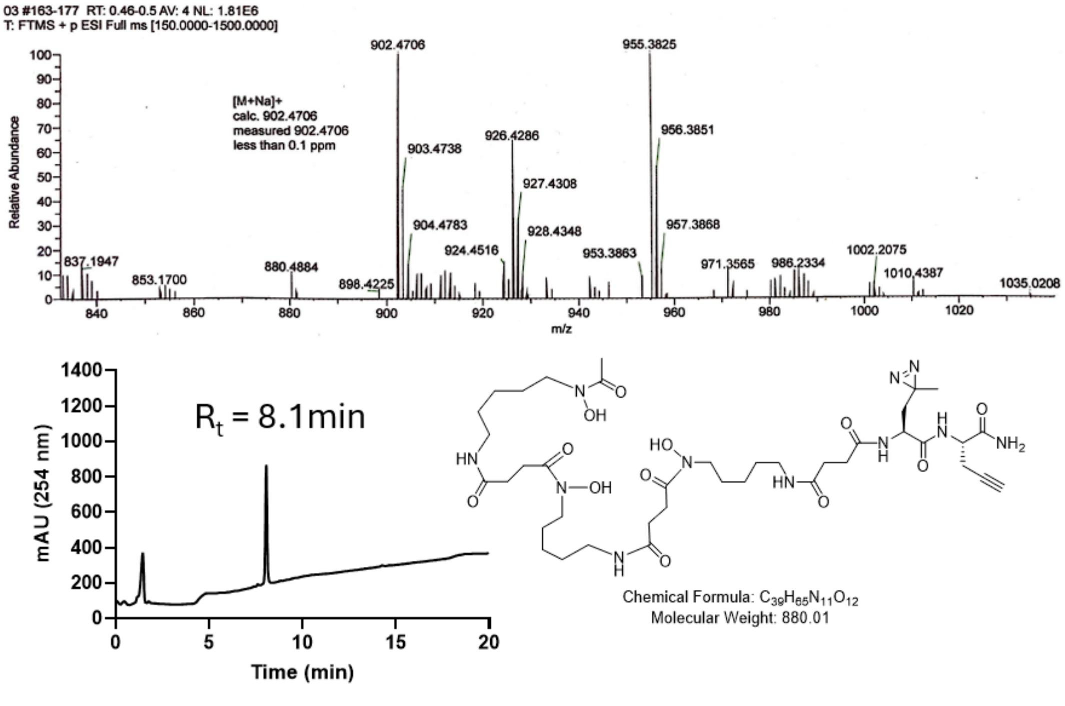

Figure S4. DFO-azir-02 HRMS and HPLC trace (Method D).

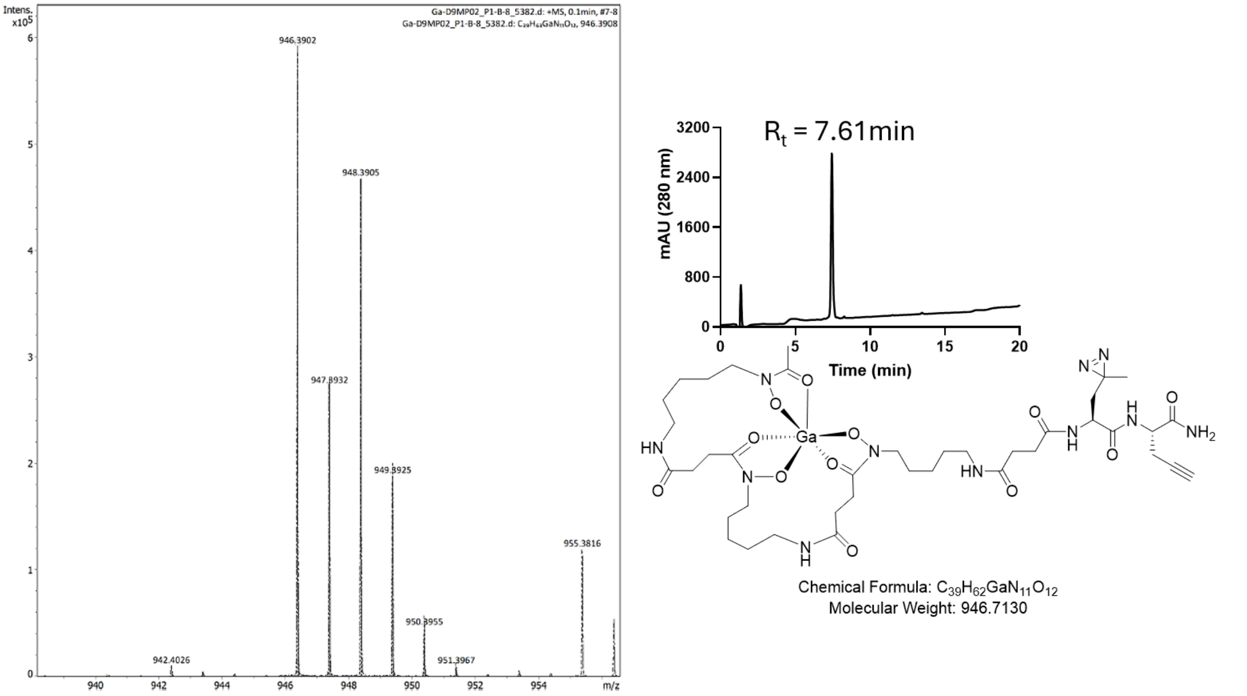

Figure S5. Ga-DFO-azir-02 HRMS and HPLC trace (Method D).

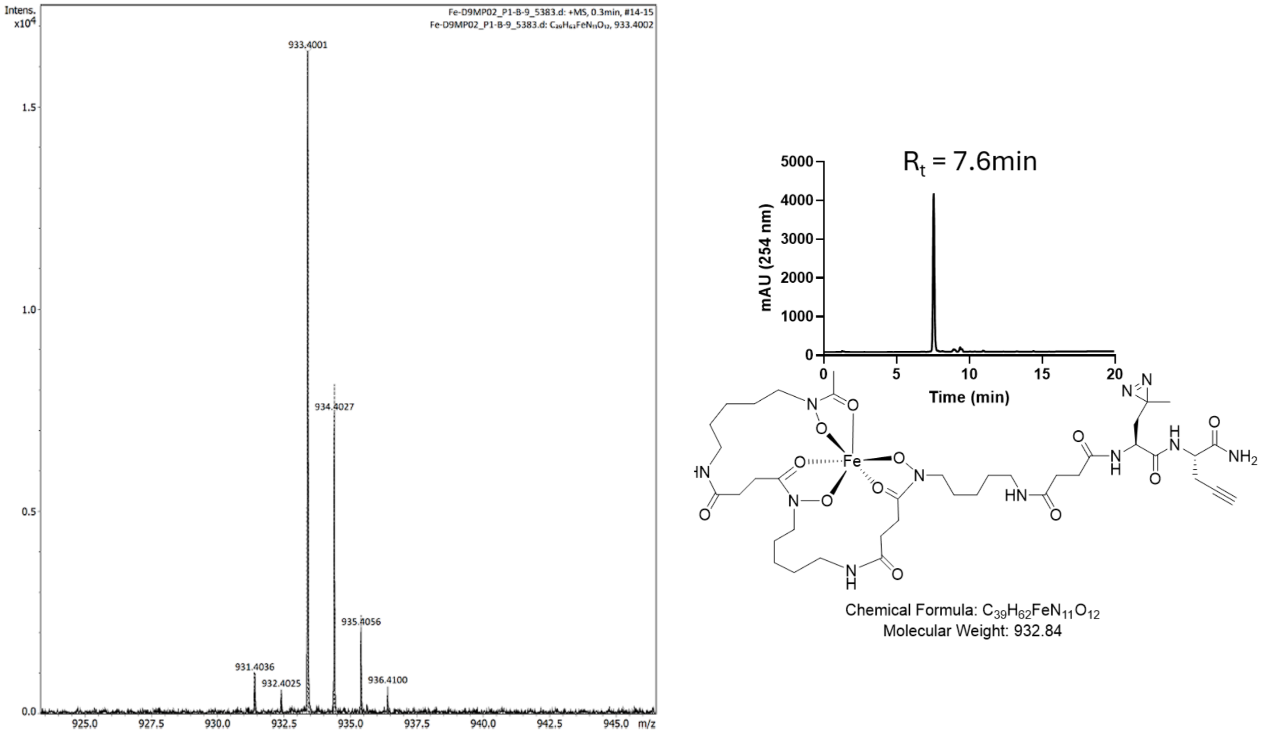

Figure S6. Fe-DFO-azir-02 HRMS and HPLC trace (Method D).

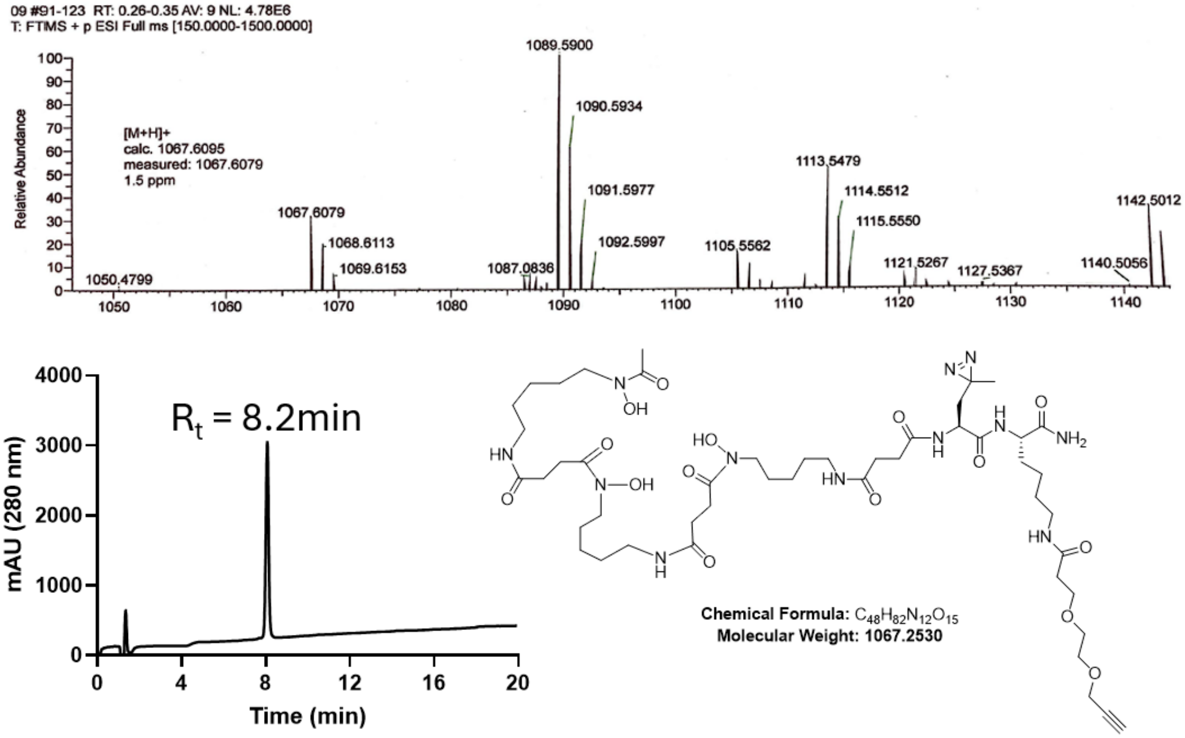

Figure S7. DFO-azir-03 HRMS and HPLC trace (Method D).

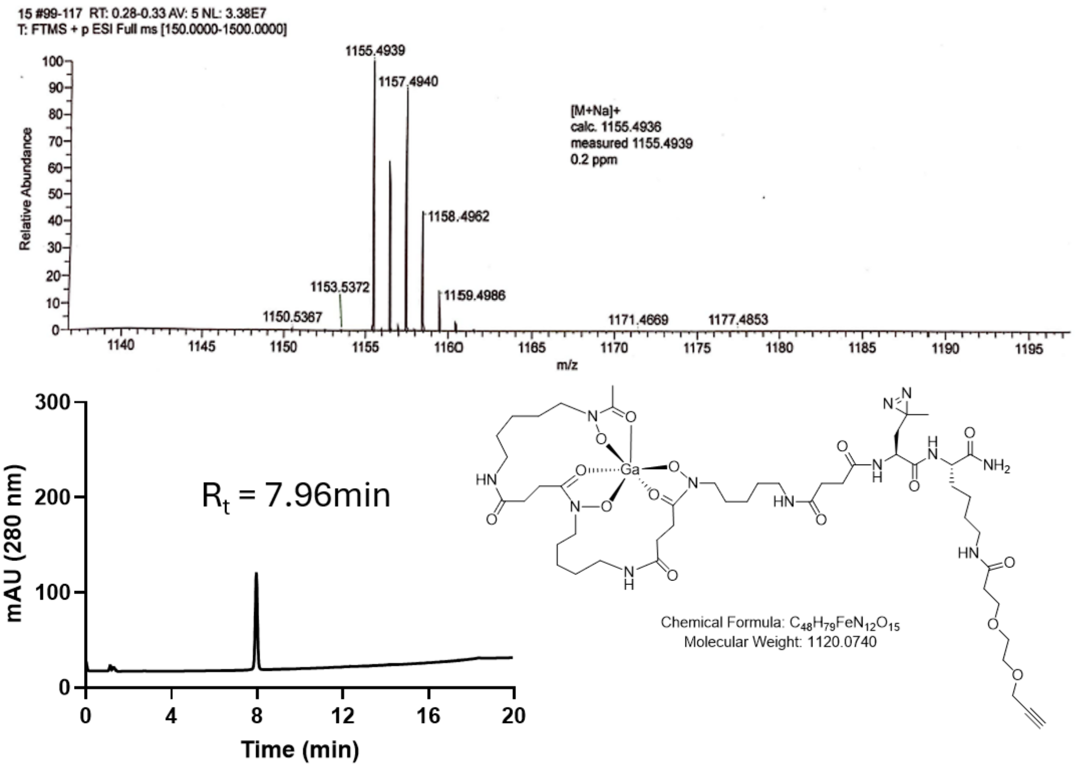

Figure S8. Ga-DFO-azir-03 HRMS and HPLC trace (Method D).

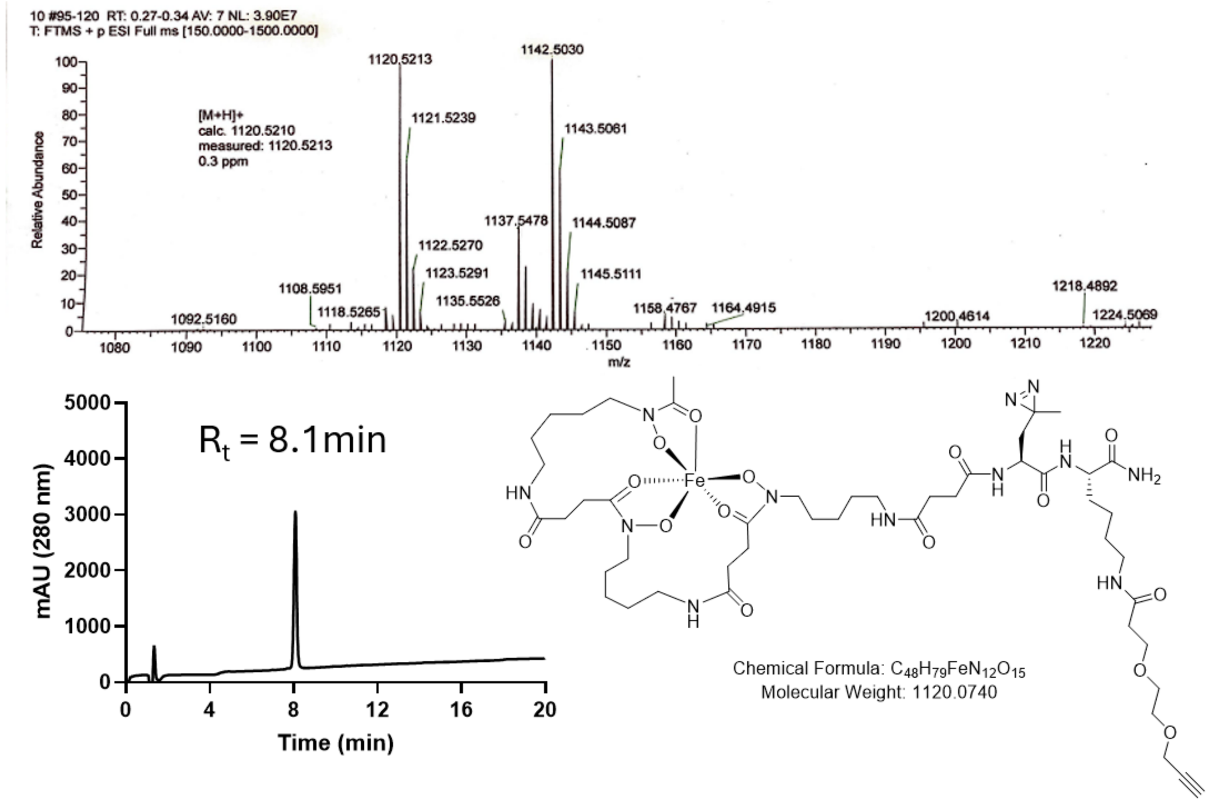

Figure S9. Fe-DFO-azir-03 HRMS and HPLC trace (Method D).

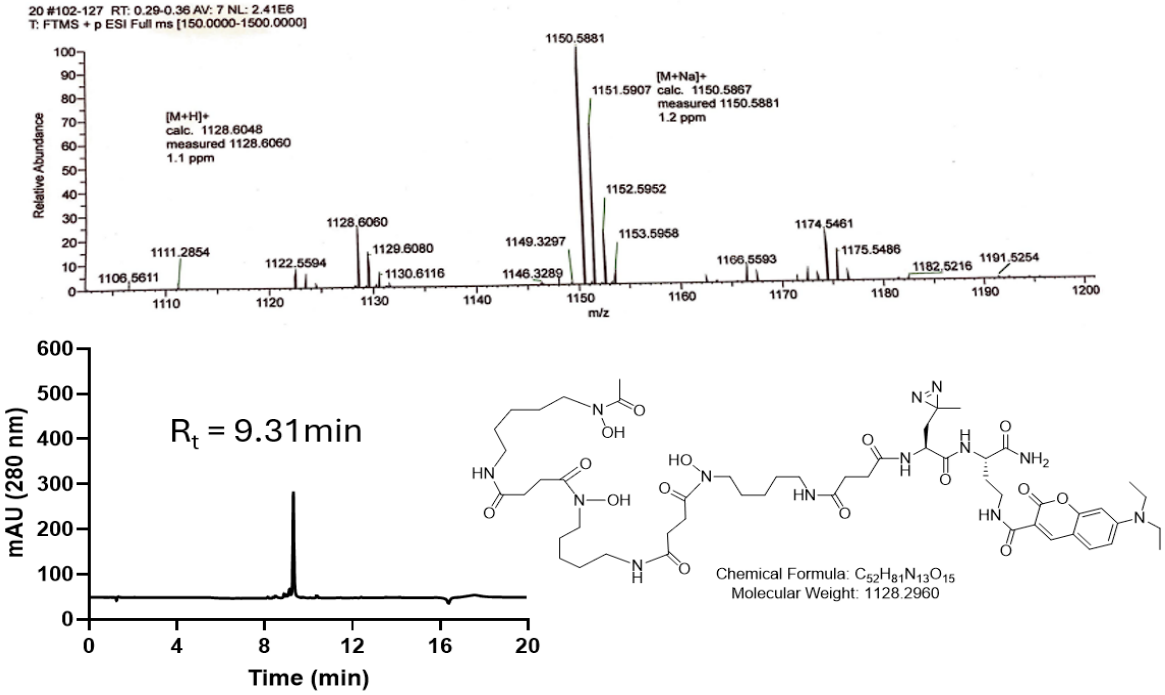

Figure S10. DFO-azir-04 HRMS and HPLC trace (Method D).

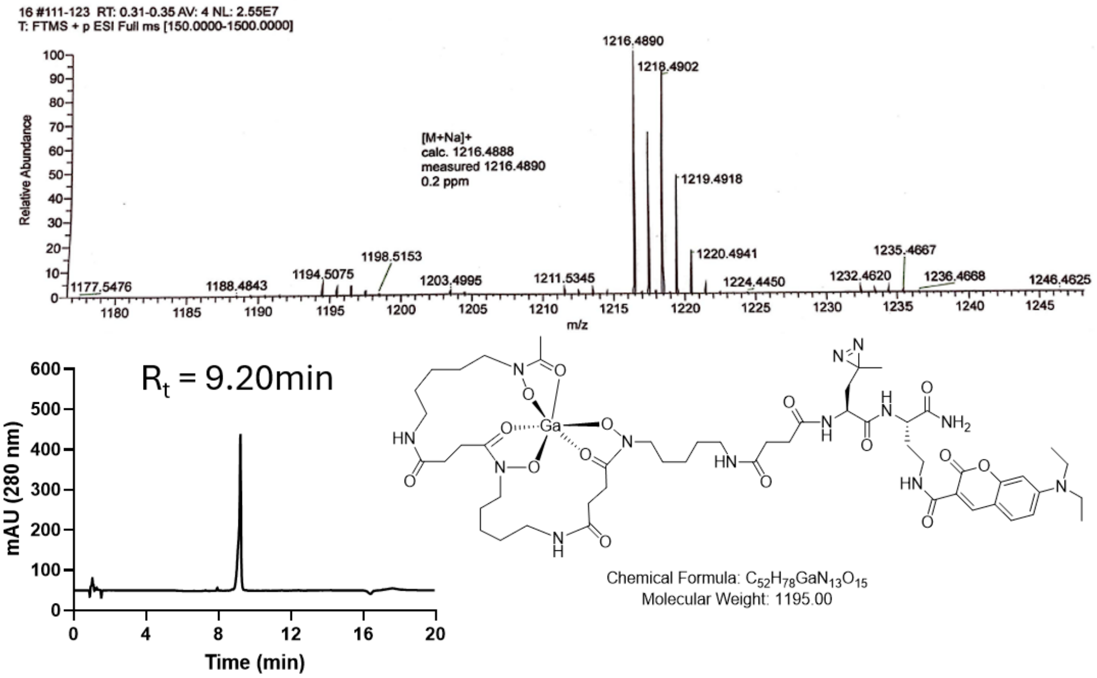

Figure S11. Ga-DFO-azir-04 HRMS and HPLC trace (Method D).

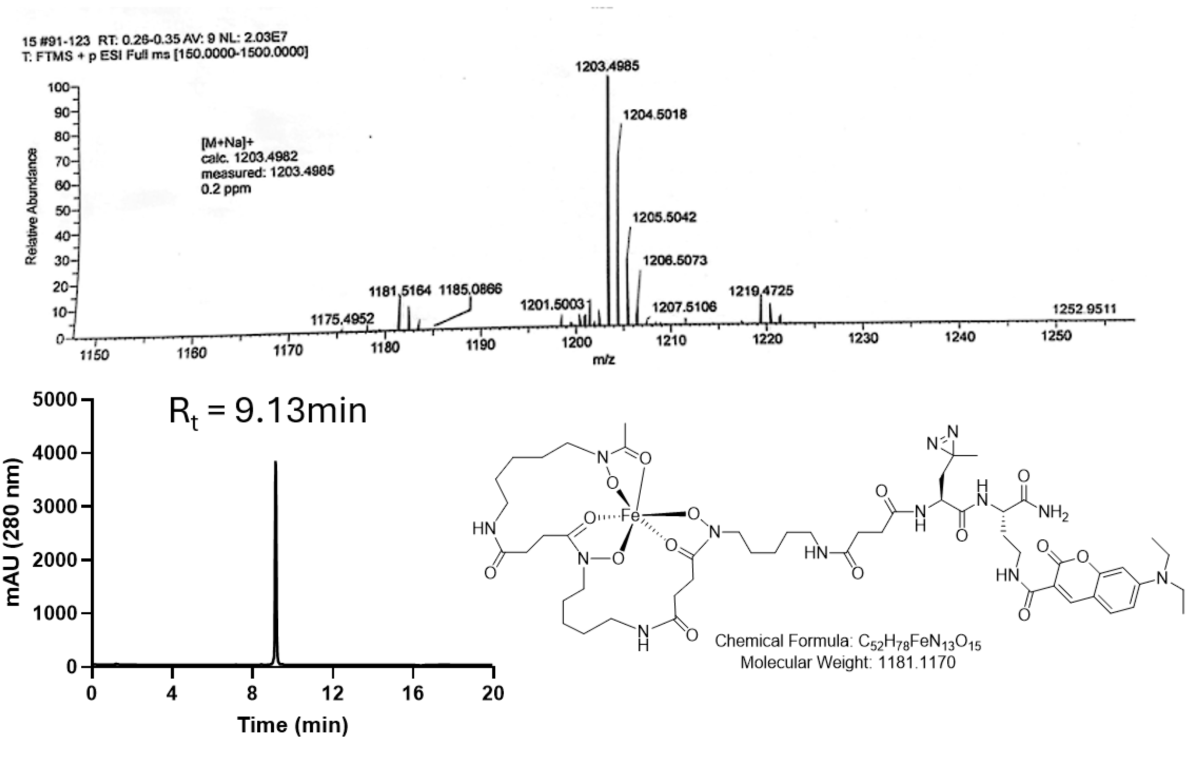

Figure S12. Fe-DFO-azir-04 HRMS and HPLC trace (Method D).

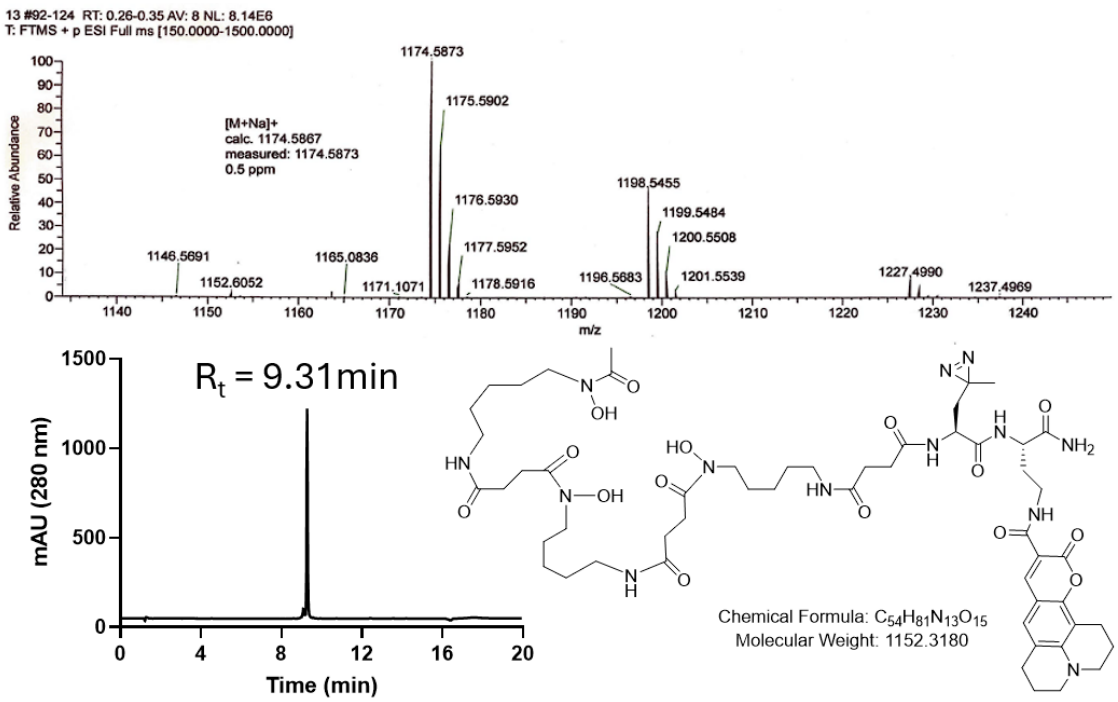

Figure S13. DFO-azir-05 HRMS and HPLC trace (Method D).

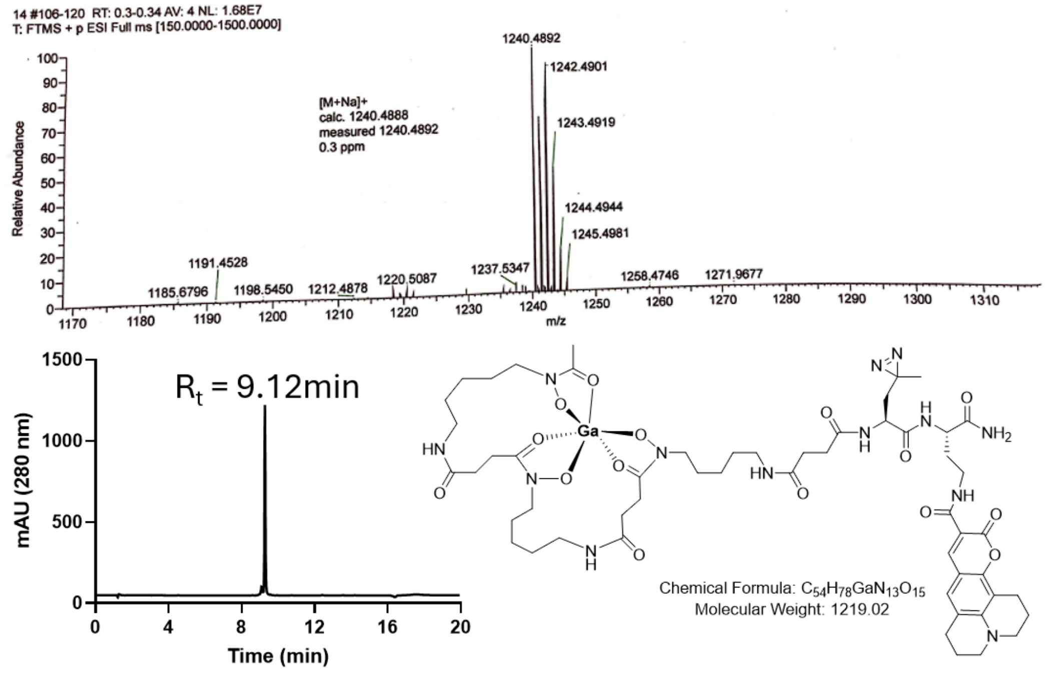

Figure S14. Ga-DFO-azir-05 HRMS and HPLC trace (Method D).

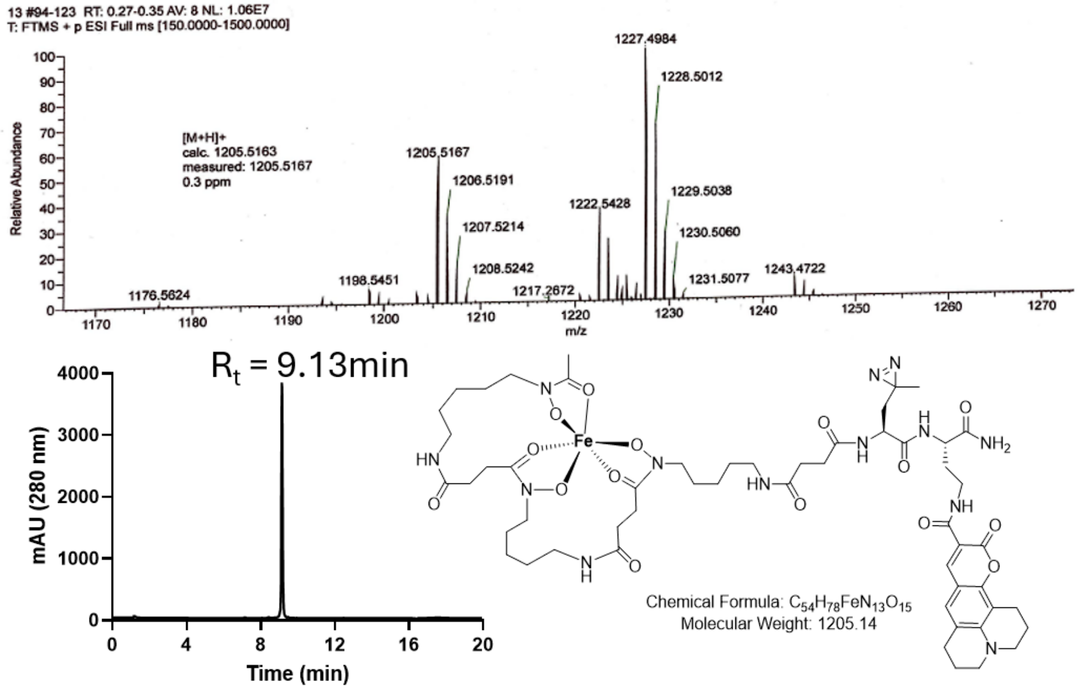

Figure S15. Fe-DFO-azir-05 HRMS and HPLC trace (Method D).

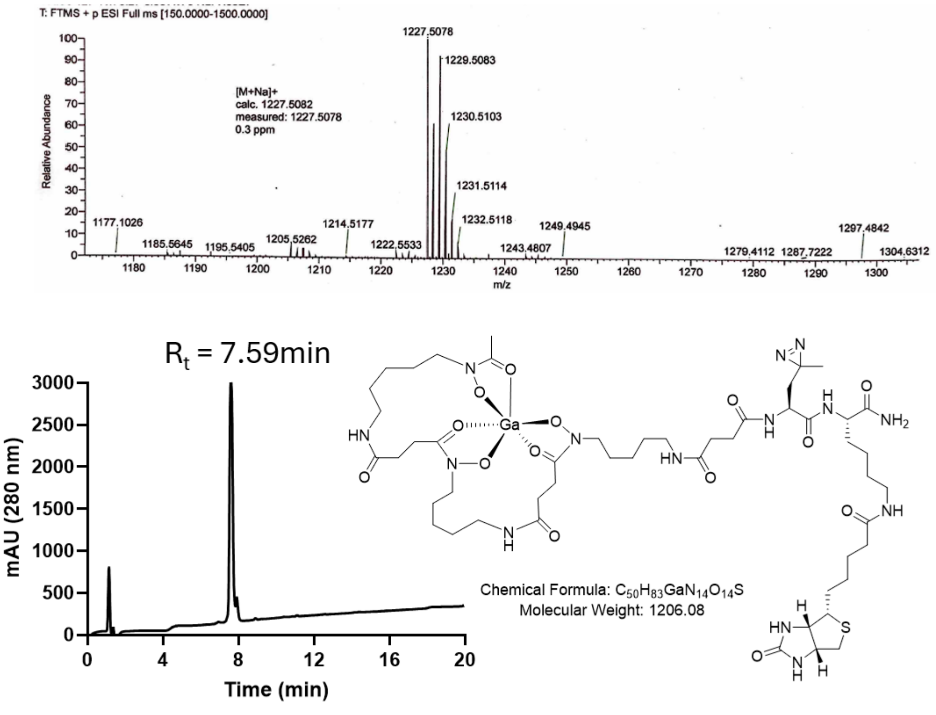

Figure S16. Ga-DFO-azir-06 HRMS and HPLC trace (Method D).

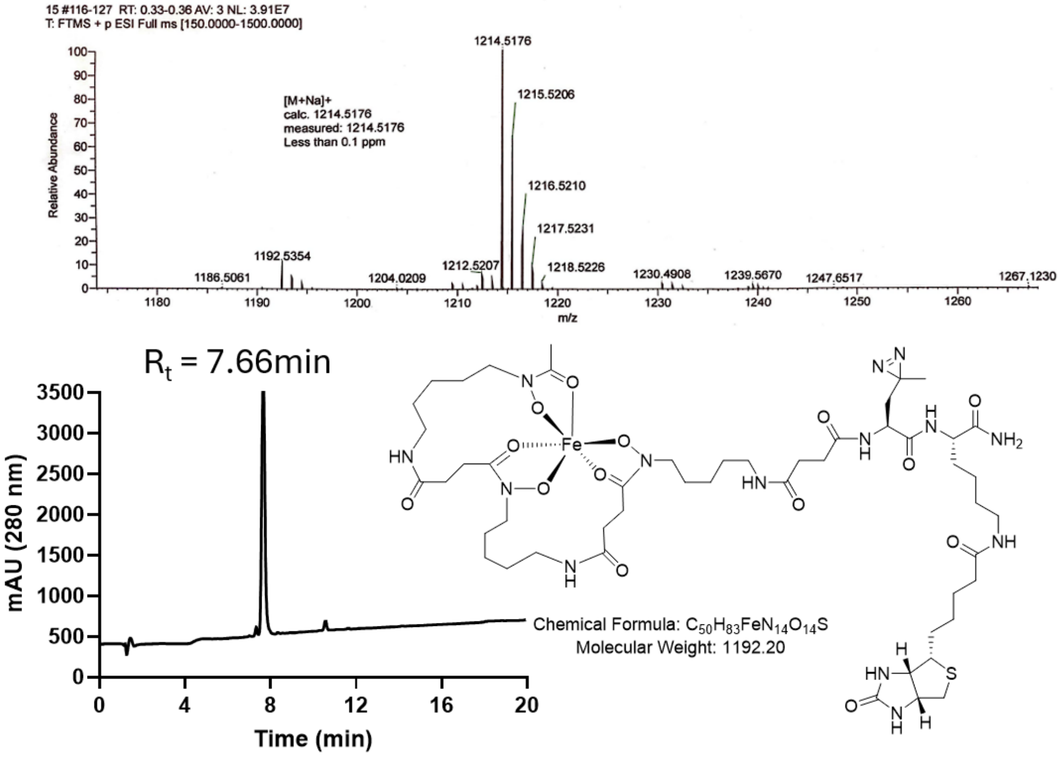

Figure S17. Fe-DFO-azir-06 HRMS and HPLC trace (Method D).

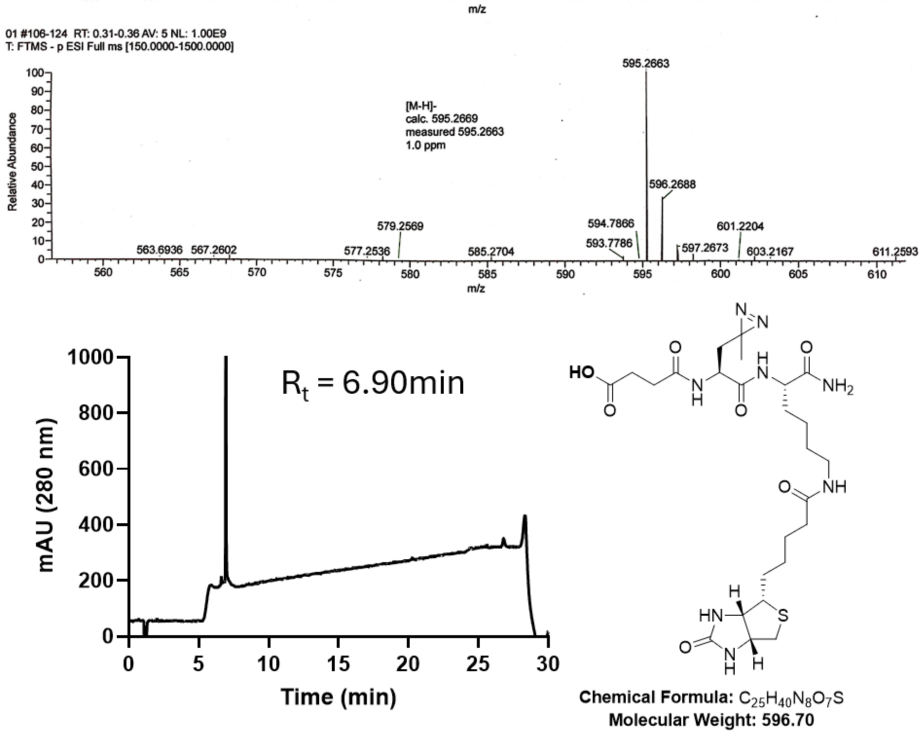

Figure S18. azir-biotin HRMS and HPLC trace.

1.3.2 NMR spectra

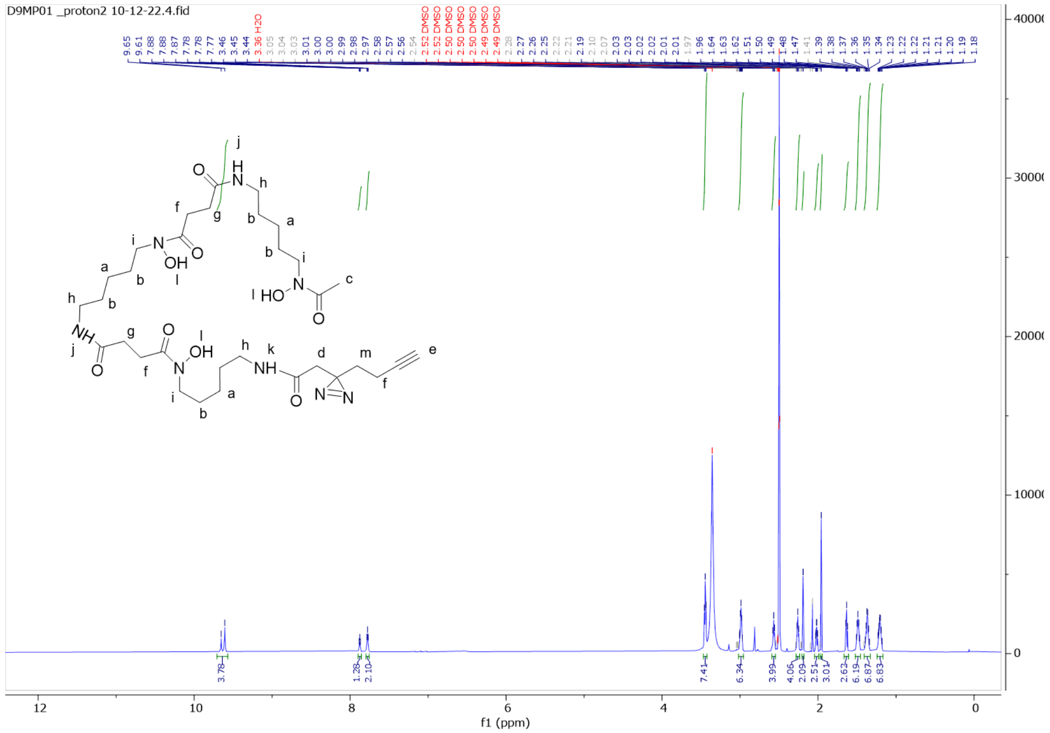

Figure S19. ^{1}H-NMR spectrum of **DFO-azir-01**. 500 MHz, DMSO-d$_6$.

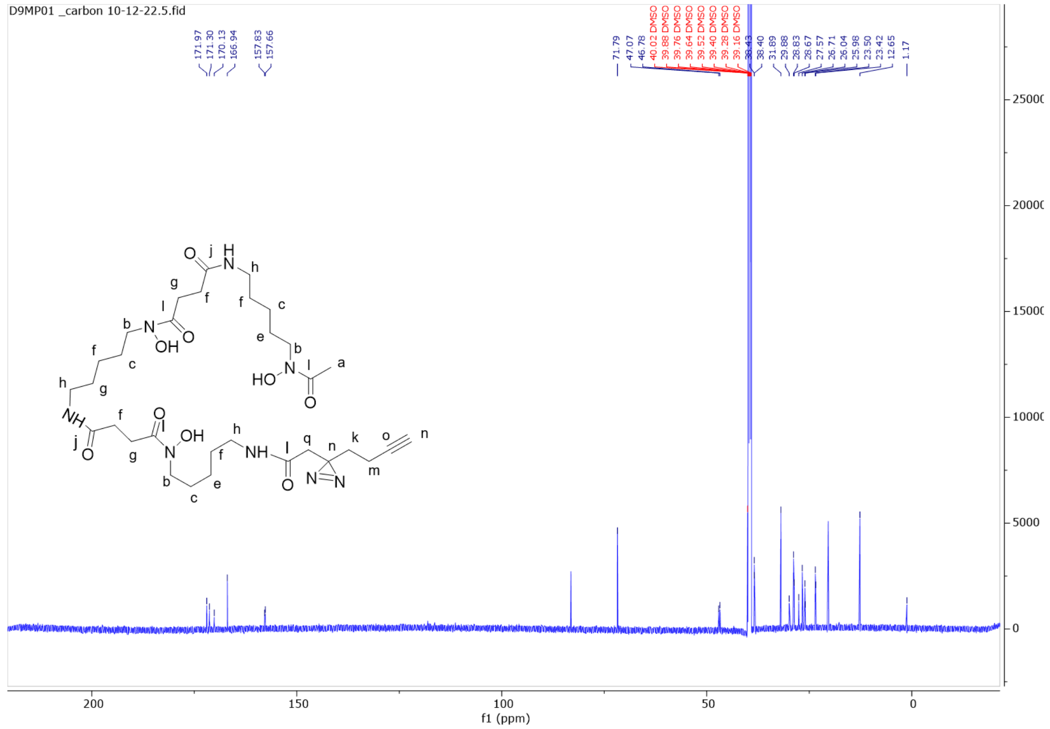

Figure S20. ^{13}C-NMR spectrum of **DFO-azir-01**. 500 MHz, DMSO-d$_6$.

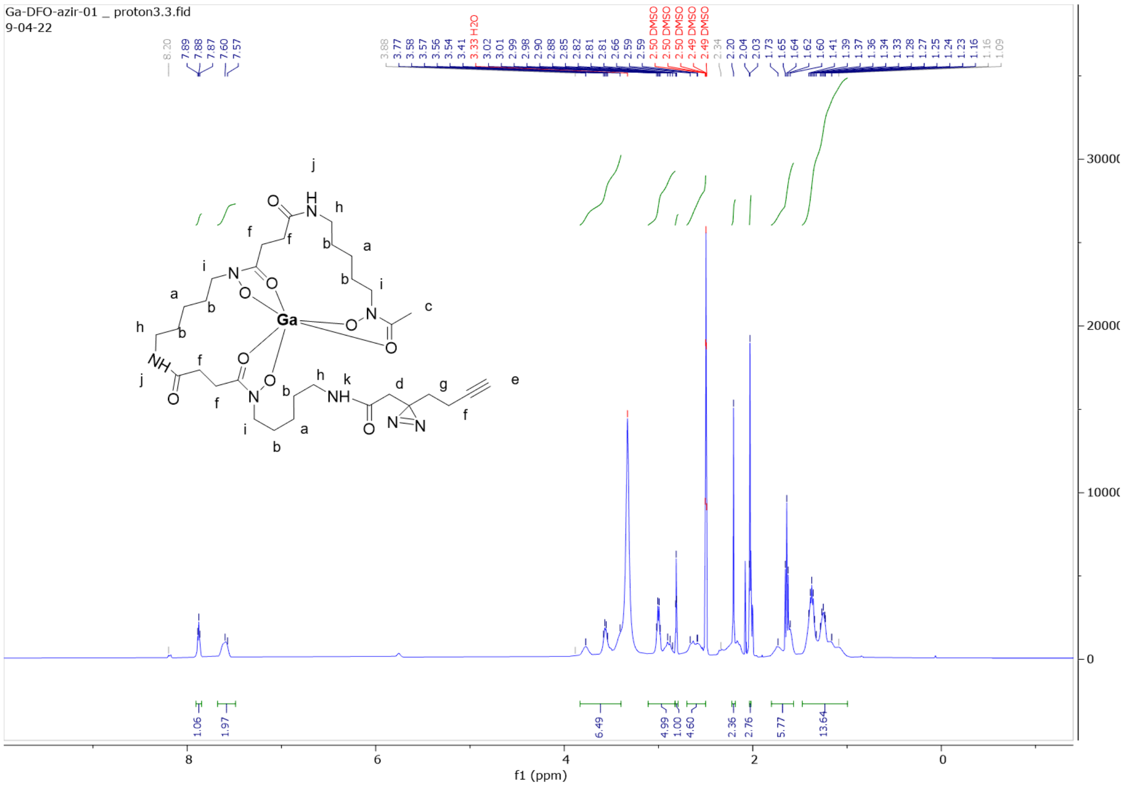

Figure S21. ^{1}H-NMR spectrum of **Ga-DFO-azir-01**. 500 MHz, DMSO-d_6.

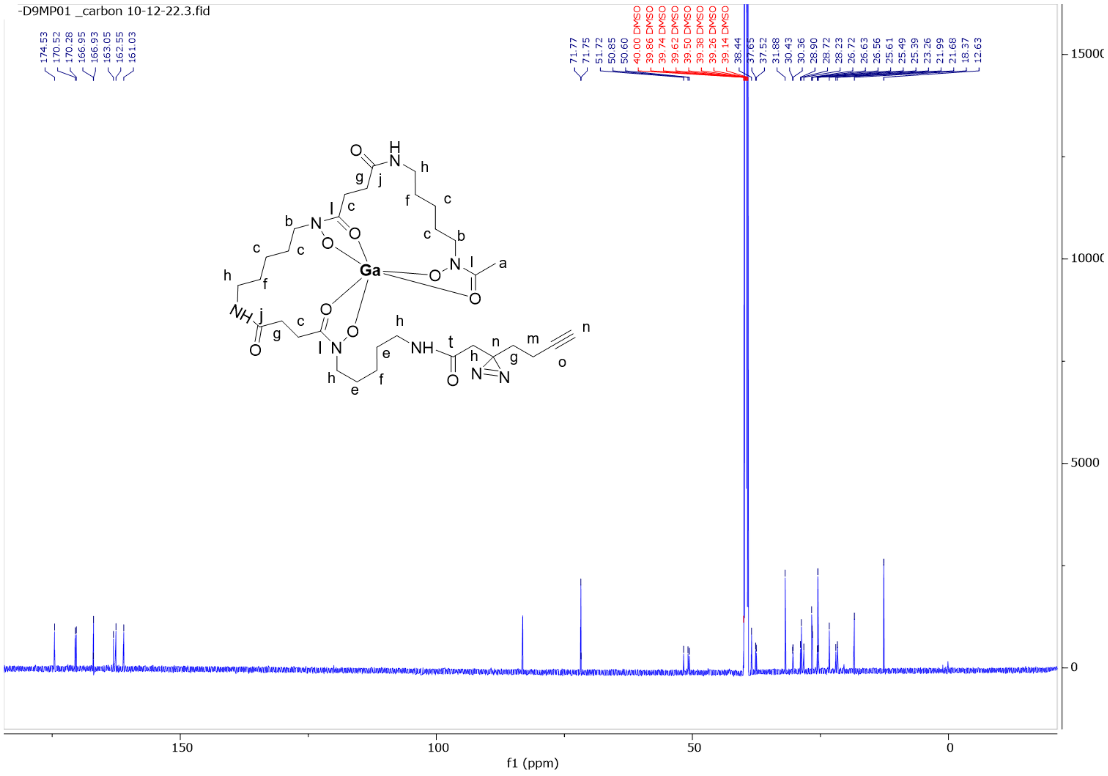

Figure S22. ^{13}C-NMR spectrum of **Ga-DFO-azir-01**. 500 MHz, DMSO-d_6.

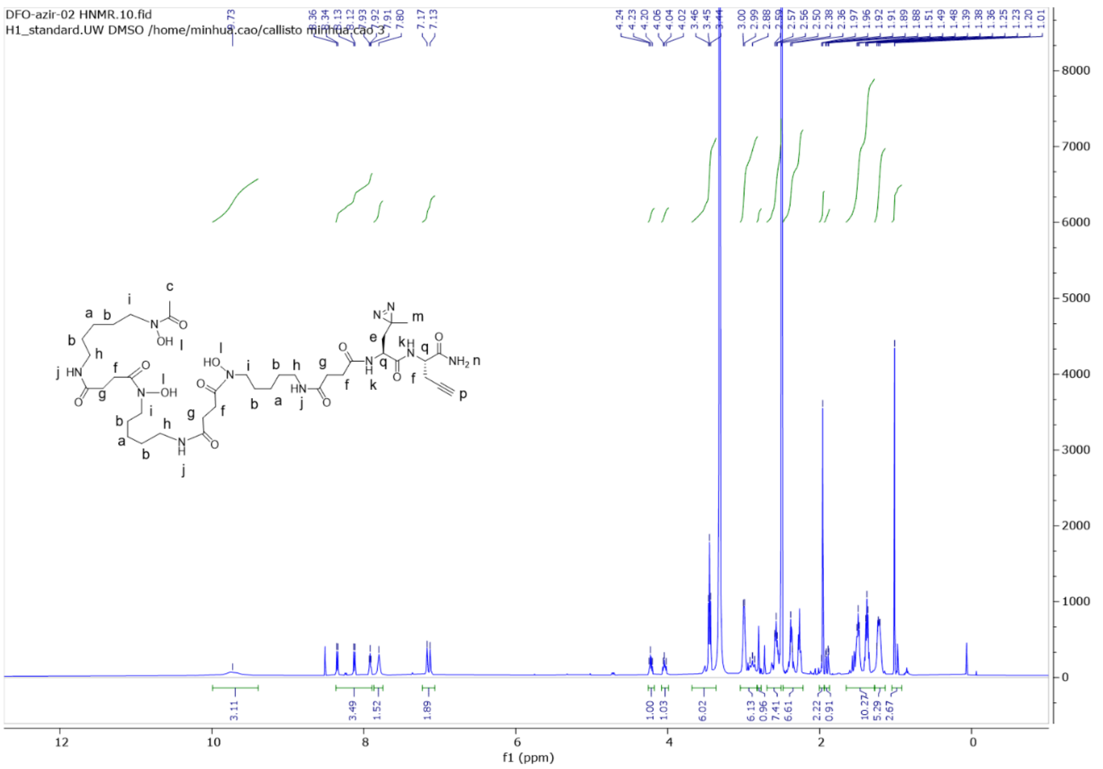

Figure S23. ^{1}H-NMR spectrum of **DFO-azir-02**. 500 MHz, DMSO-d$_6$.

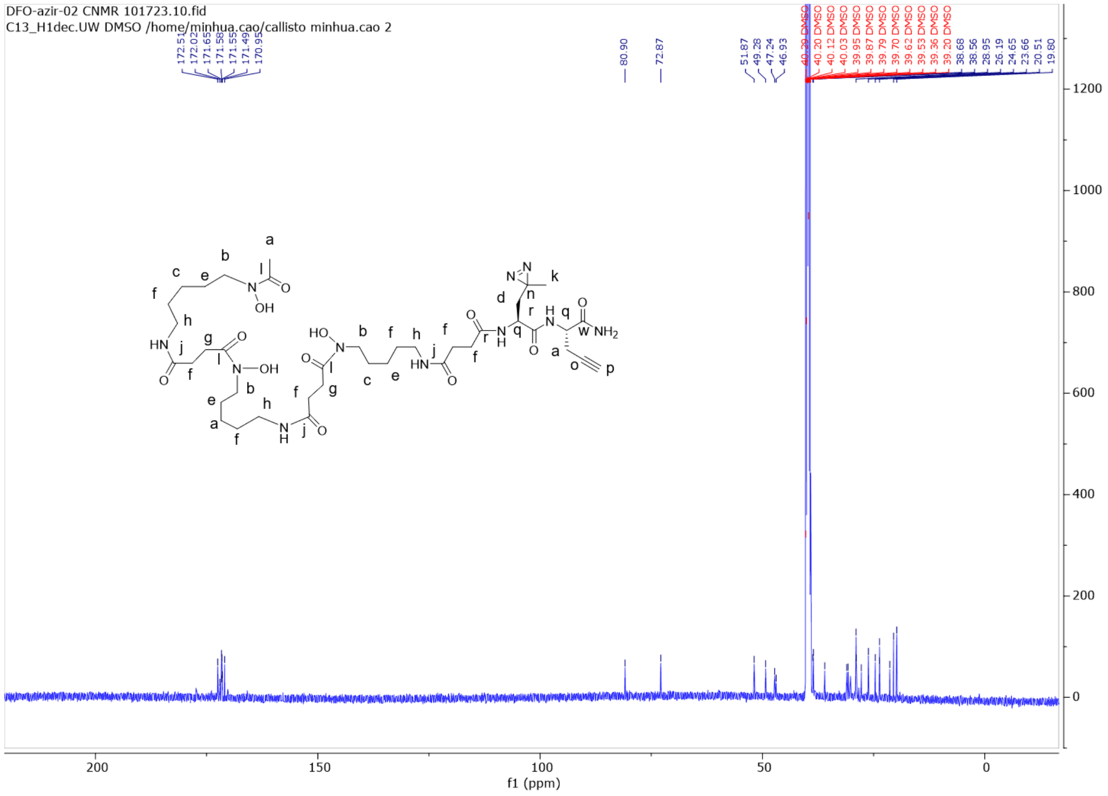

Figure S24. ^{13}C-NMR spectrum of **DFO-azir-02**. 500 MHz, DMSO-d$_6$.

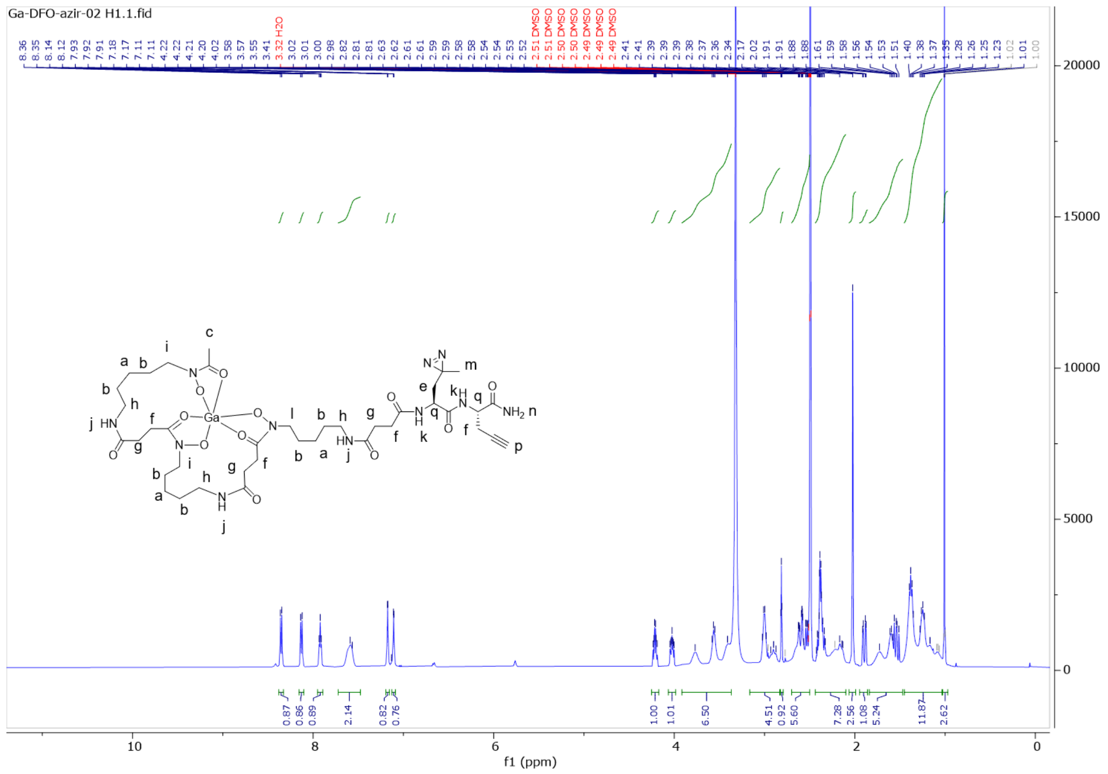

Figure S25. ^{1}H-NMR spectrum of **Ga-DFO-azir-02**. 500 MHz, DMSO-d$_6$.

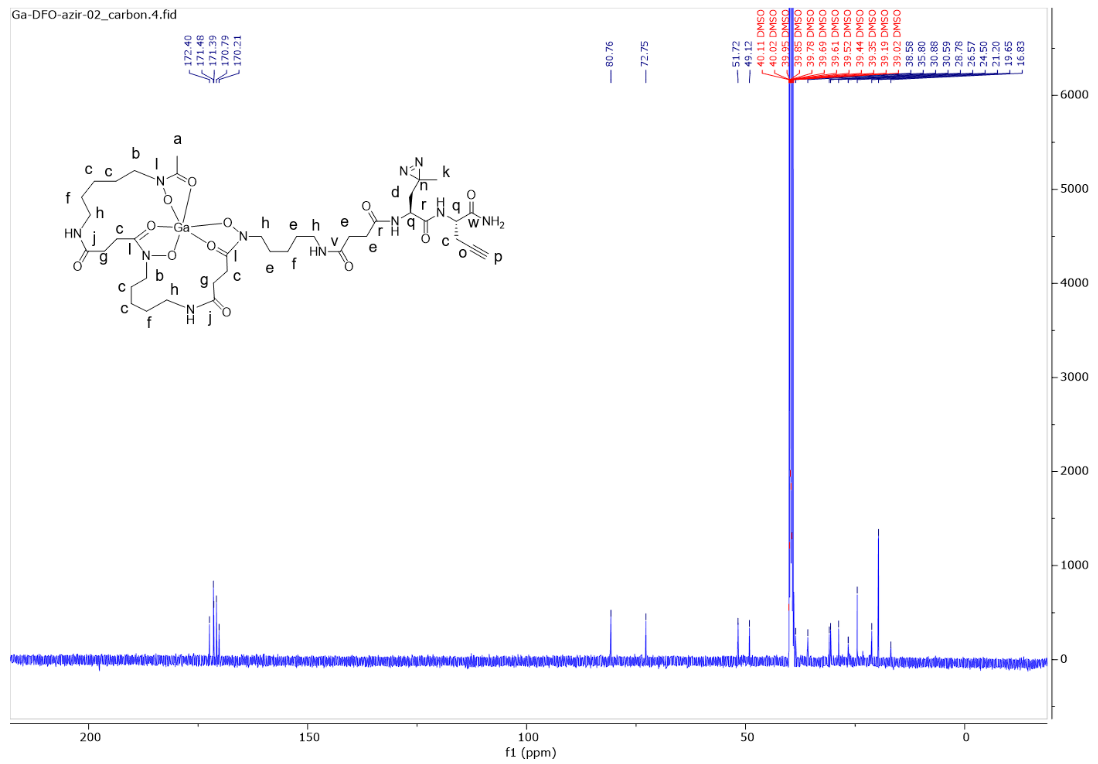

Figure S26. ^{13}C-NMR spectrum of **Ga-DFO-azir-02**. 500 MHz, DMSO-d$_6$.

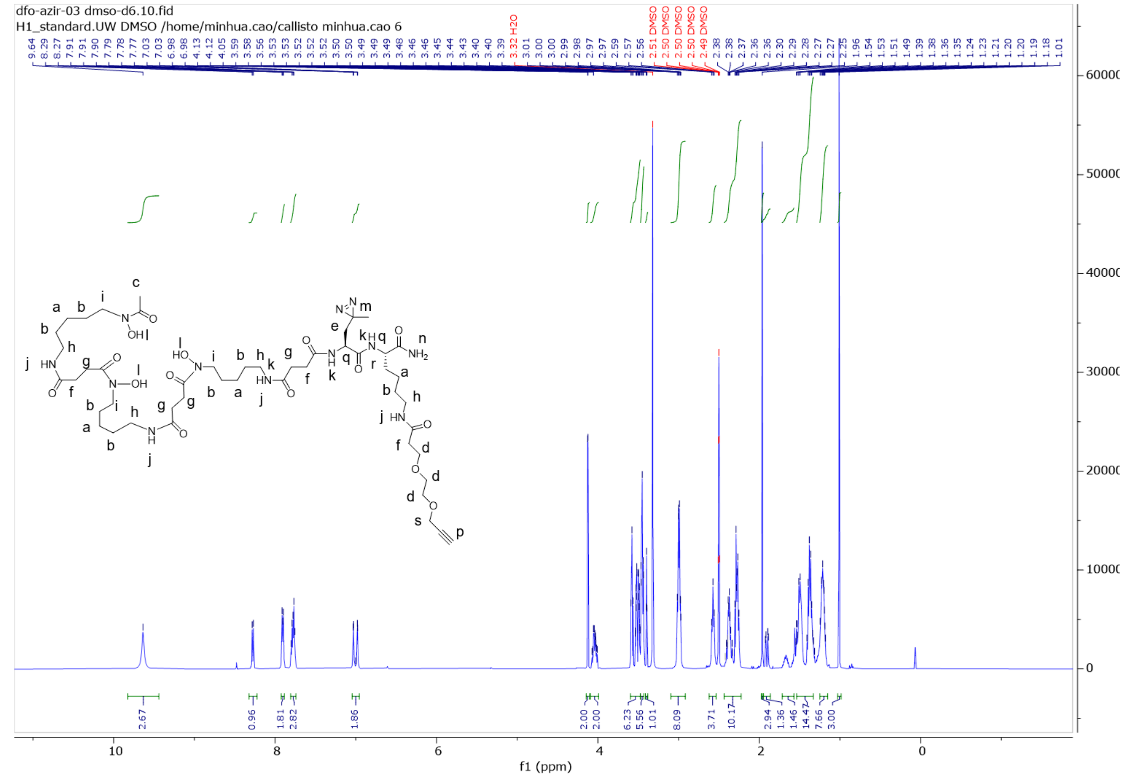

Figure S27. ^{1}H-NMR spectrum of **DFO-azir-03**. 500 MHz, DMSO-d_6.

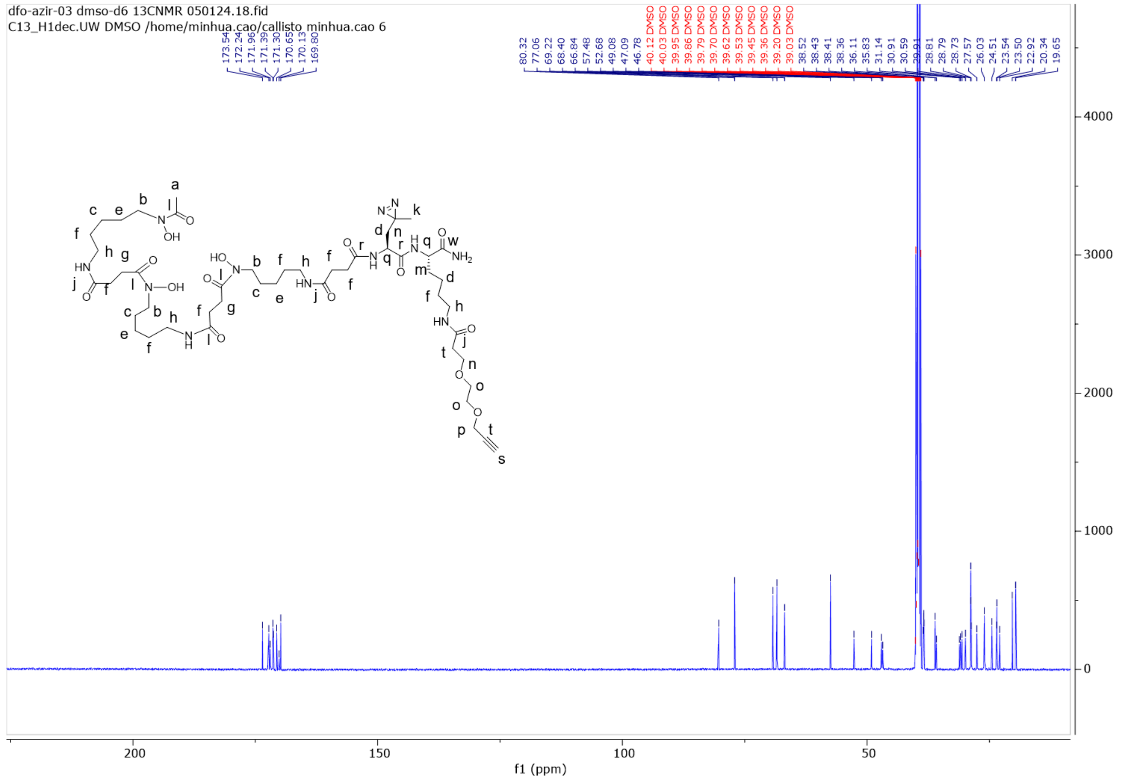

Figure S28. ^{13}C-NMR spectrum of **DFO-azir-03**. 500 MHz, DMSO-d_6.

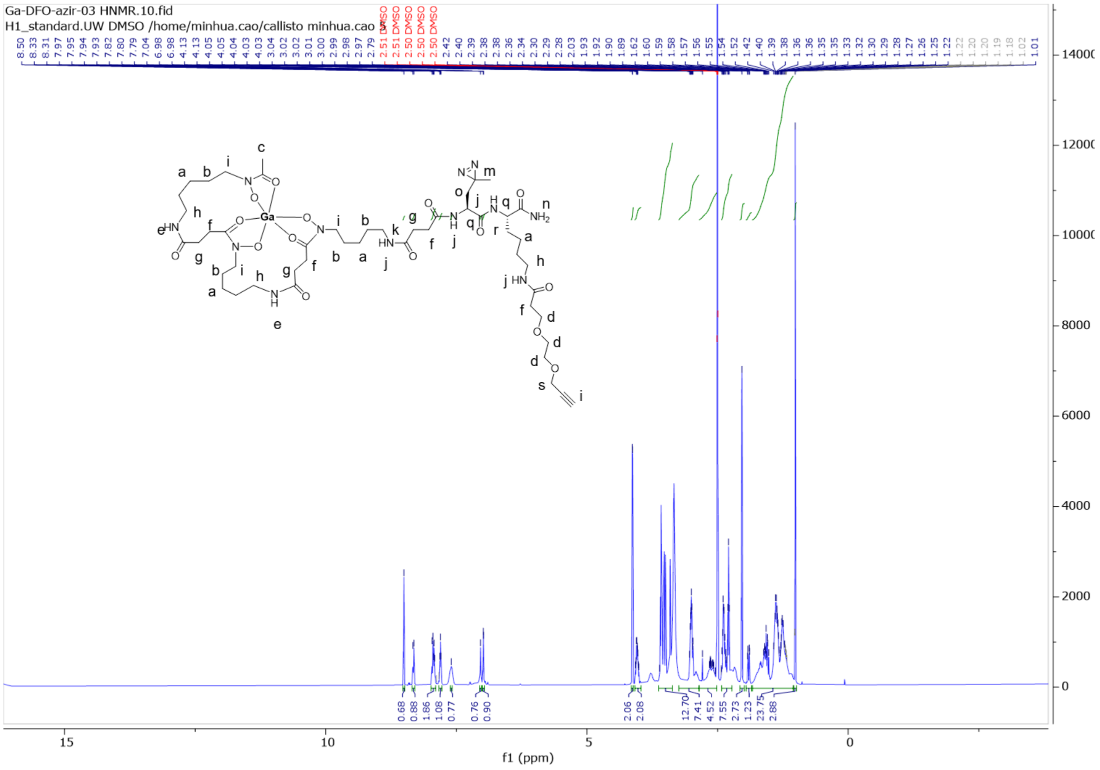

Figure S29. ^{1}H-NMR spectrum of **Ga-DFO-azir-03**. 500 MHz, DMSO-d_6.

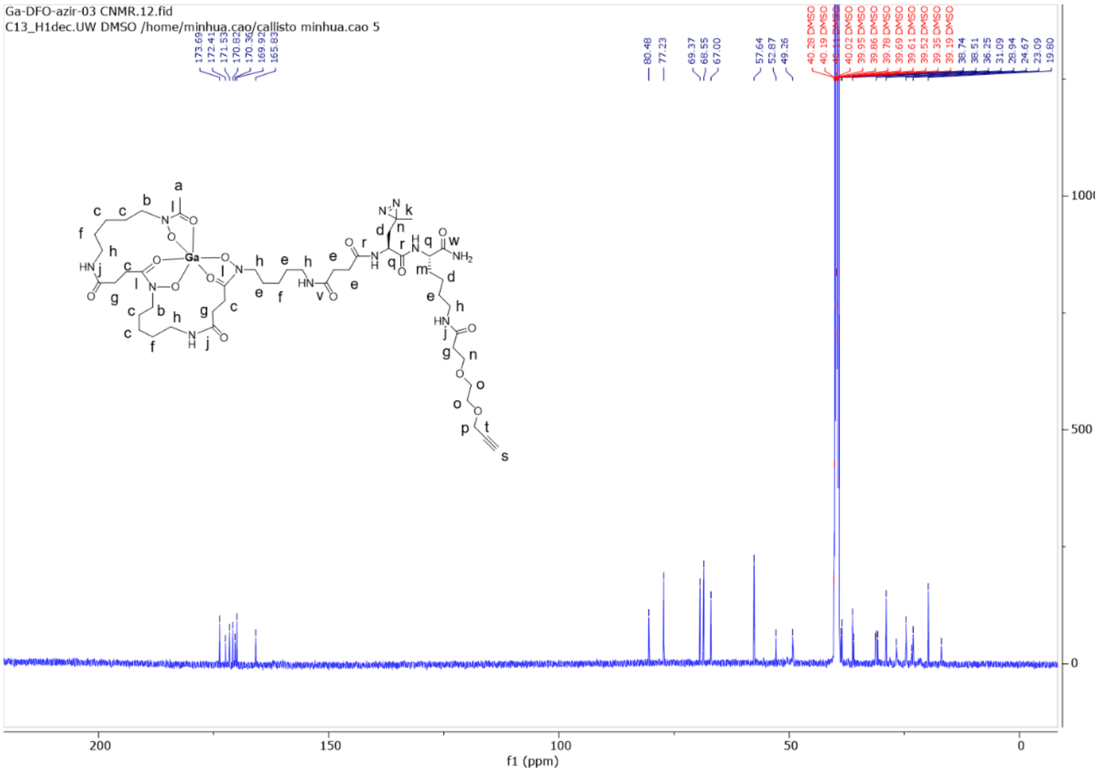

Figure S30. ^{13}C-NMR spectrum of **Ga-DFO-azir-03**. 500 MHz, DMSO-d_6.

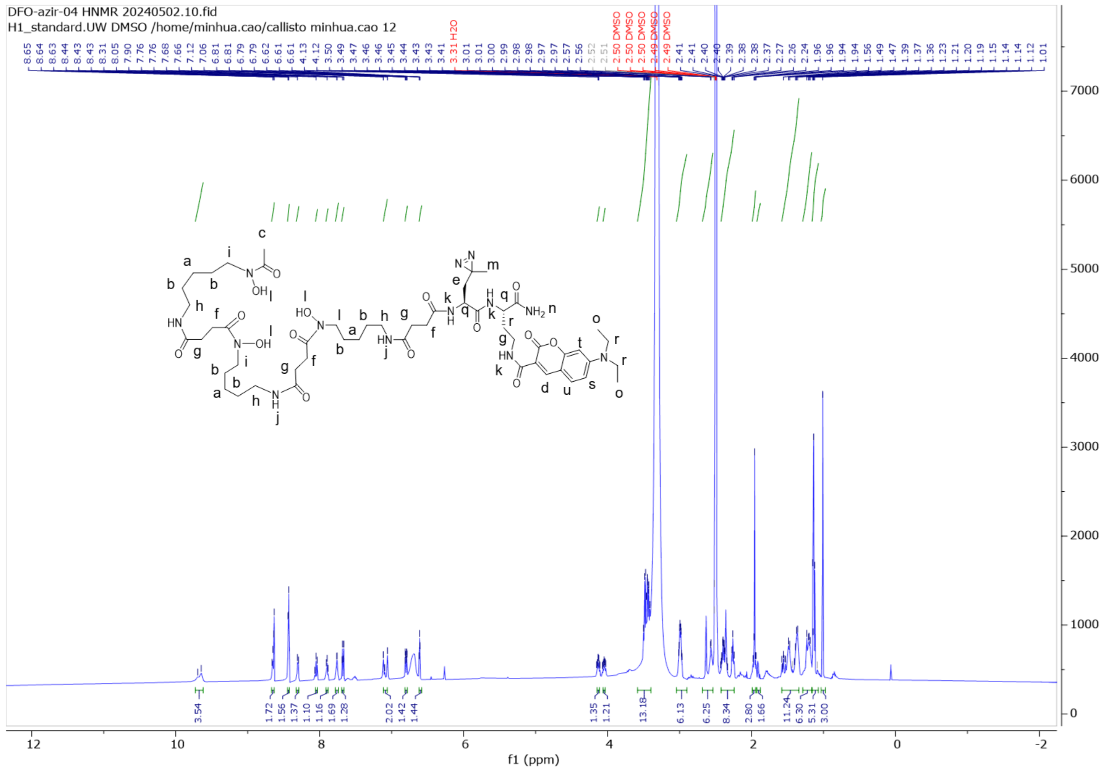

Figure S31. ^{1}H-NMR spectrum of **DFO-azir-04**. 500 MHz, DMSO-d_6.

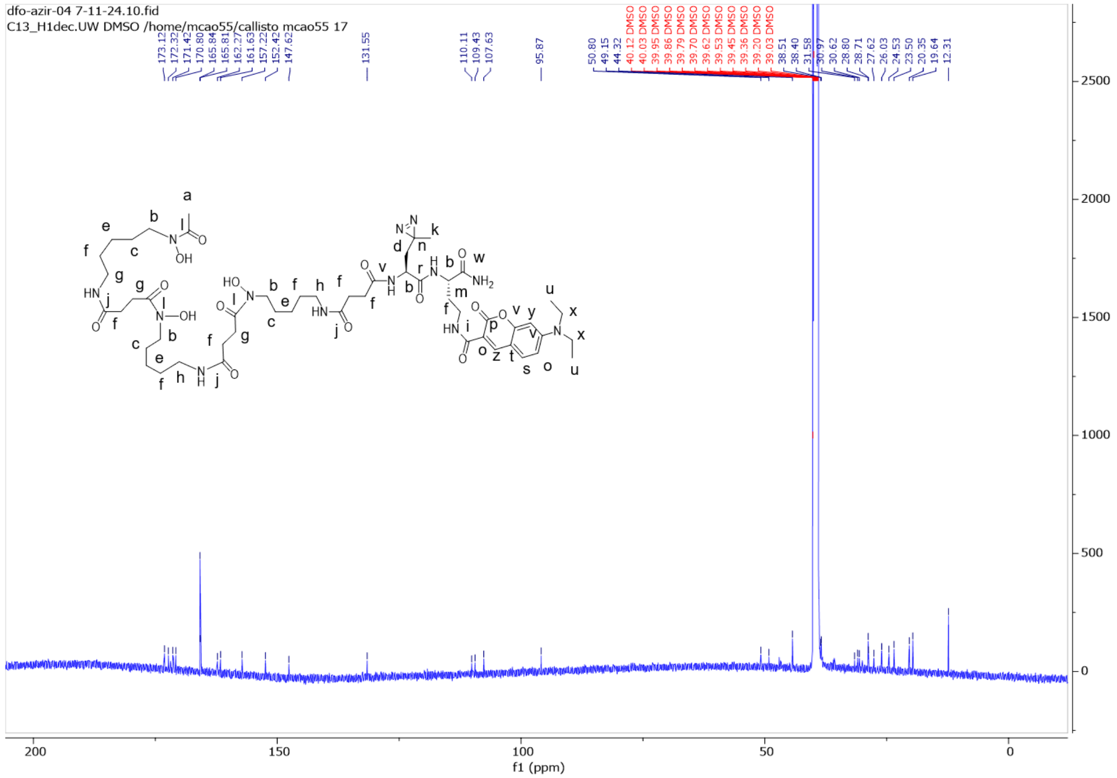

Figure S32. ^{13}C-NMR spectrum of **DFO-azir-04**. 500 MHz, DMSO-d_6.

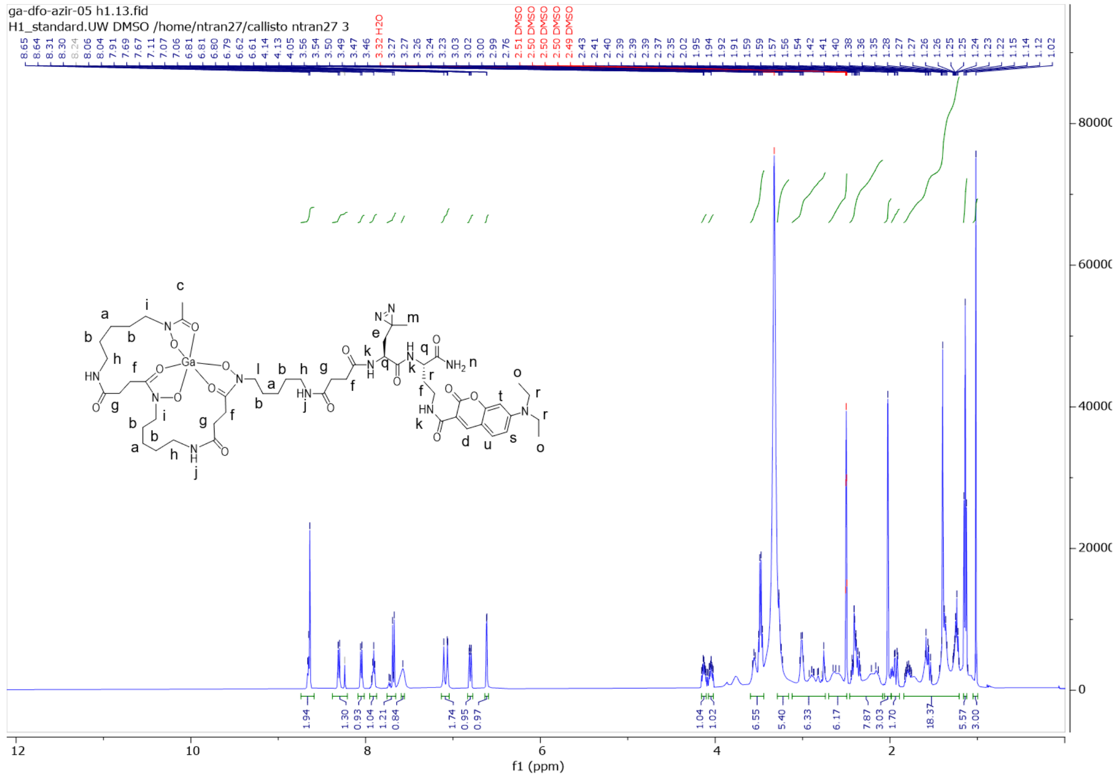

Figure S33. ^{1}H-NMR spectrum of **Ga-DFO-azir-04**. 500 MHz, DMSO-d$_6$.

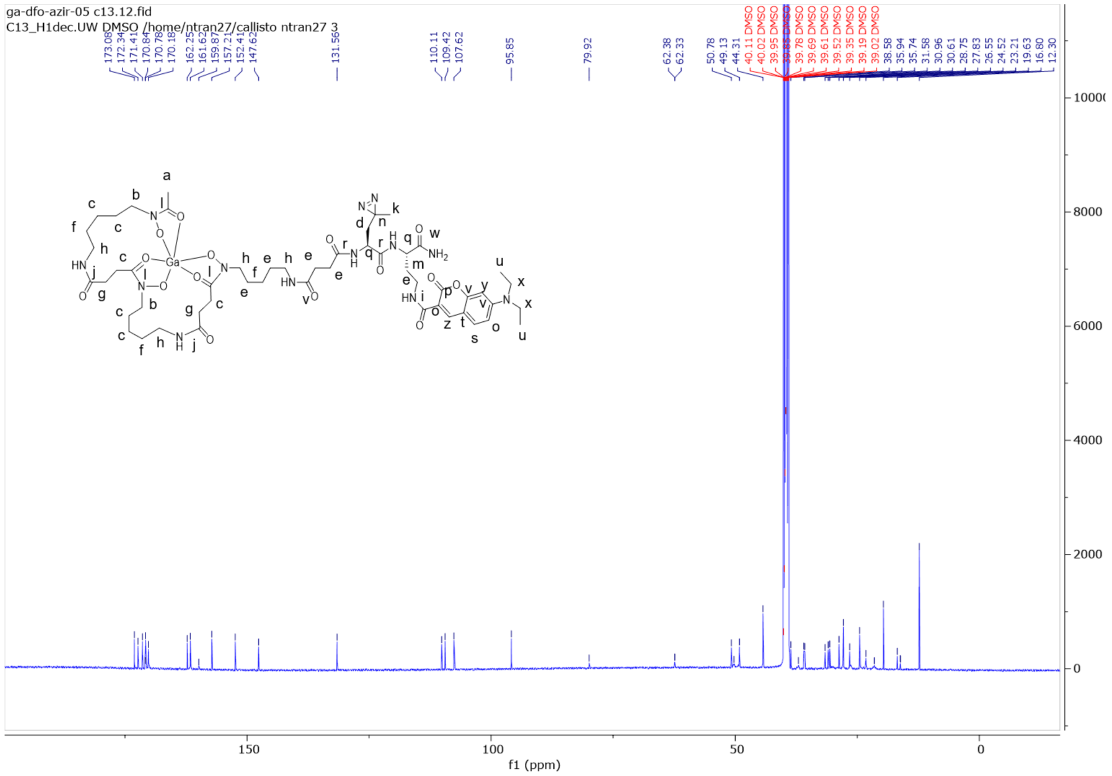

Figure S34. ^{13}C-NMR spectrum of **Ga-DFO-azir-04**. 500 MHz, DMSO-d$_6$.

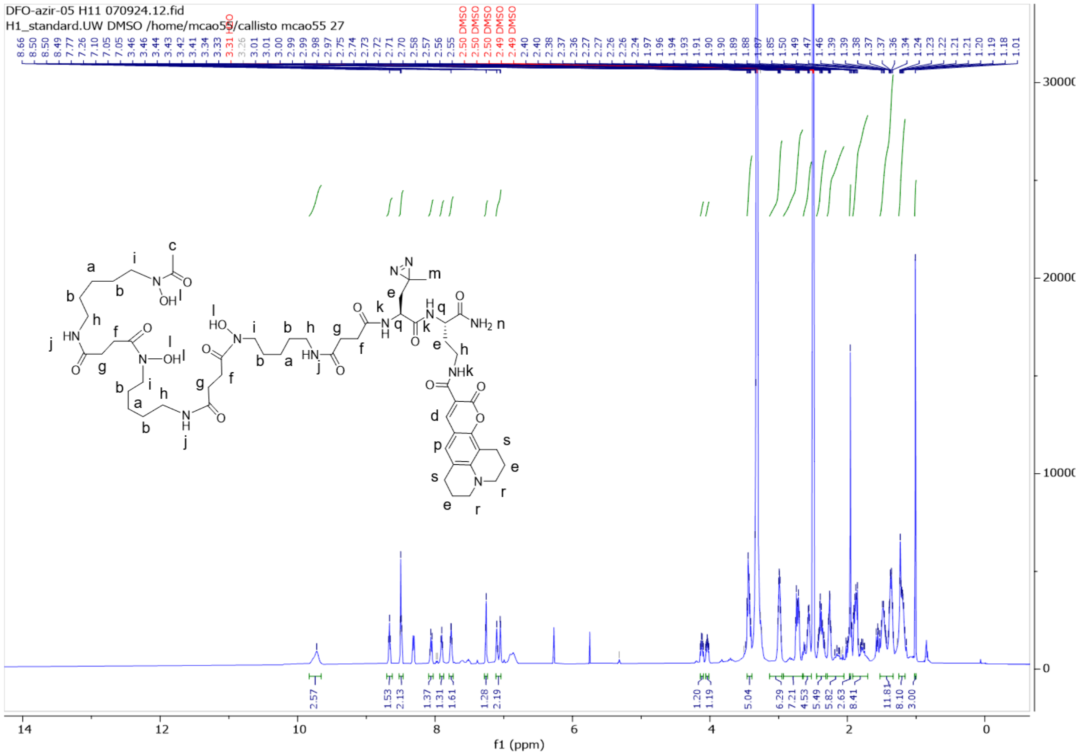

Figure S35. ^{1}H-NMR spectrum of **DFO-azir-05**. 500 MHz, DMSO-d_6.

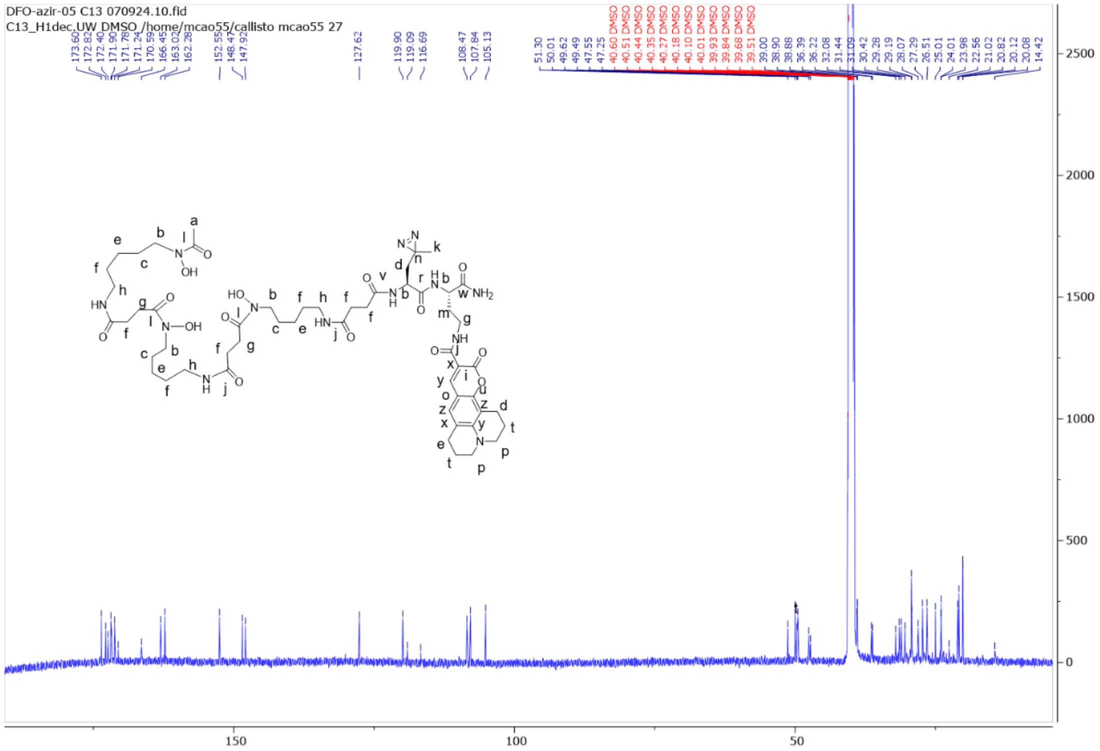

Figure S36. ^{13}C-NMR spectrum of **DFO-azir-05**. 500 MHz, DMSO-d_6.

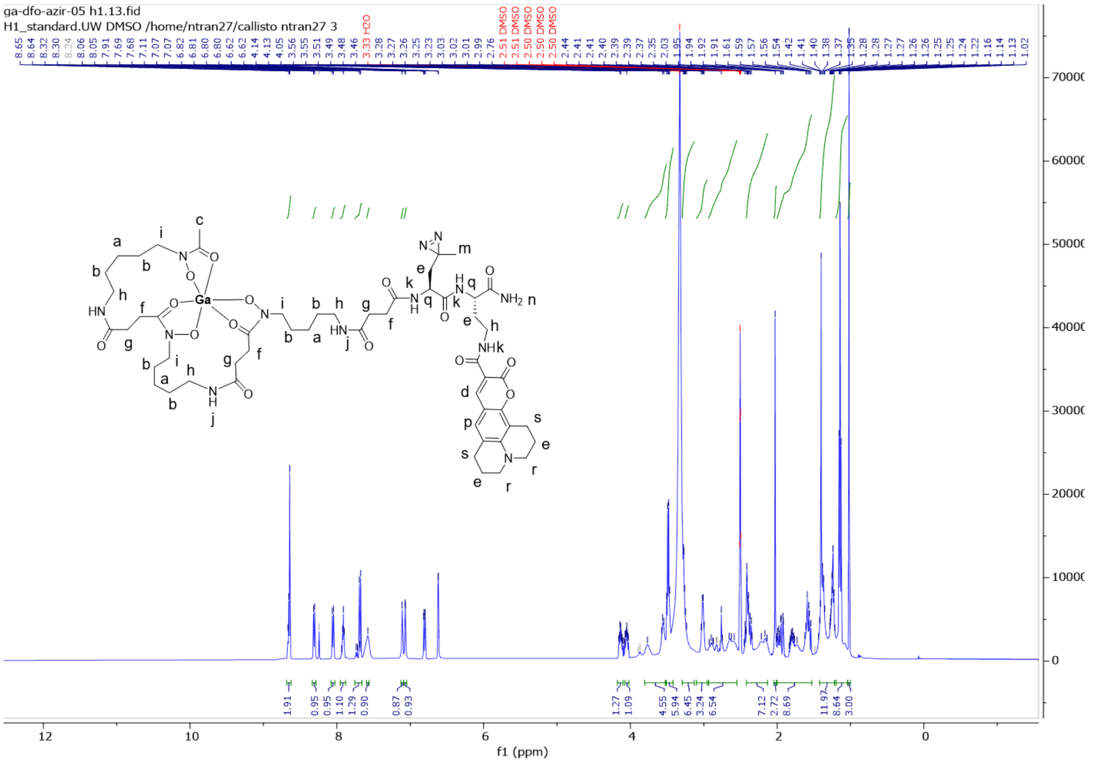

Figure S37. ^{1}H-NMR spectrum of **Ga-DFO-azir-05**. 500 MHz, DMSO-d_6.

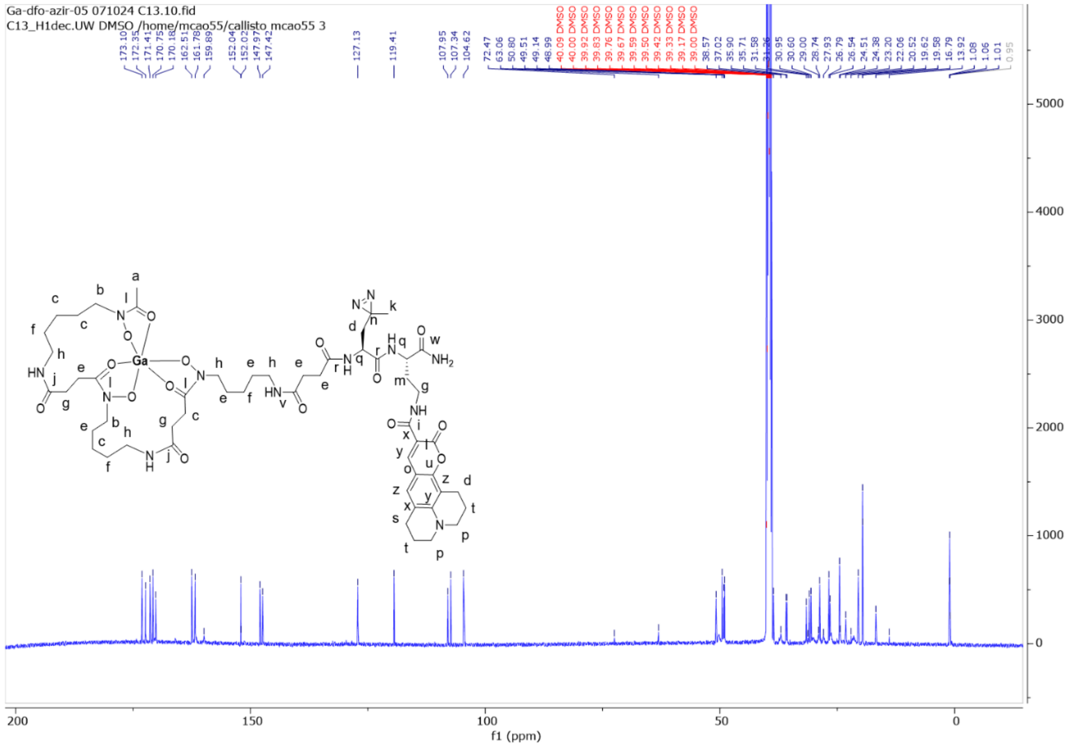

Figure S38. ^{13}C-NMR spectrum of **Ga-DFO-azir-05**. 500 MHz, DMSO-d_6.

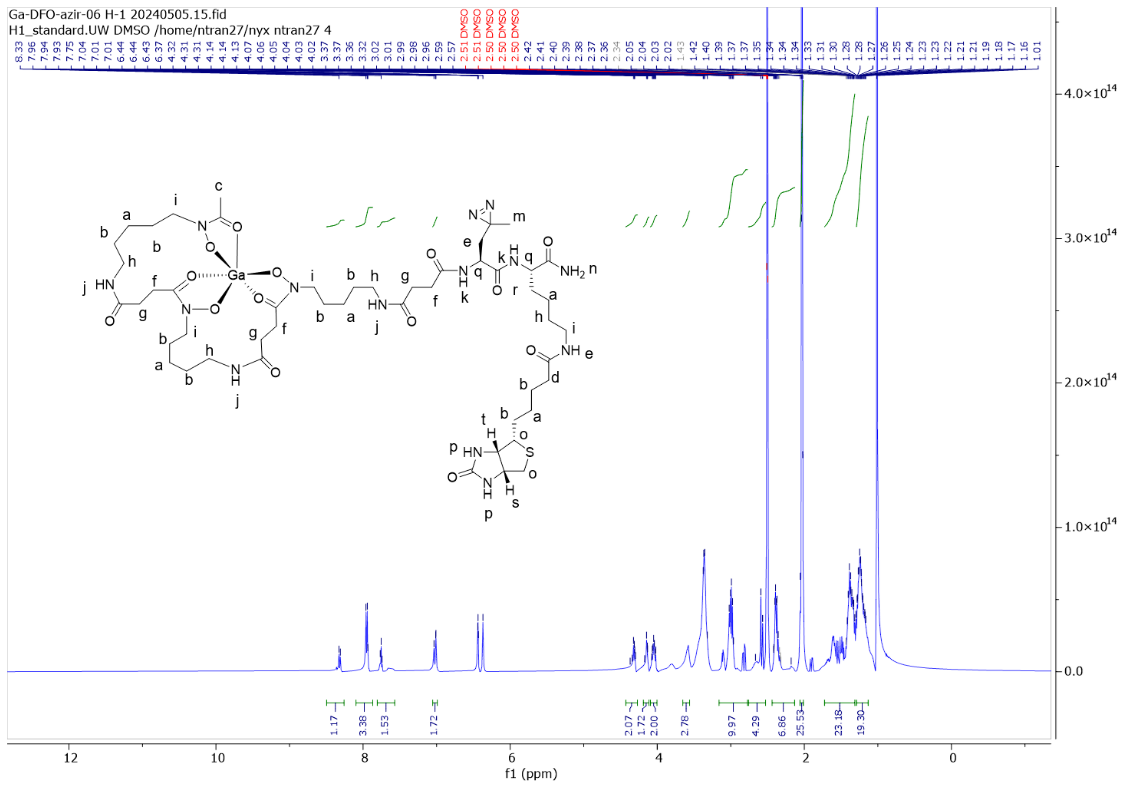

Figure S39. ^{1}H-NMR spectrum of **Ga-DFO-azir-06**. 500 MHz, DMSO-d_6.

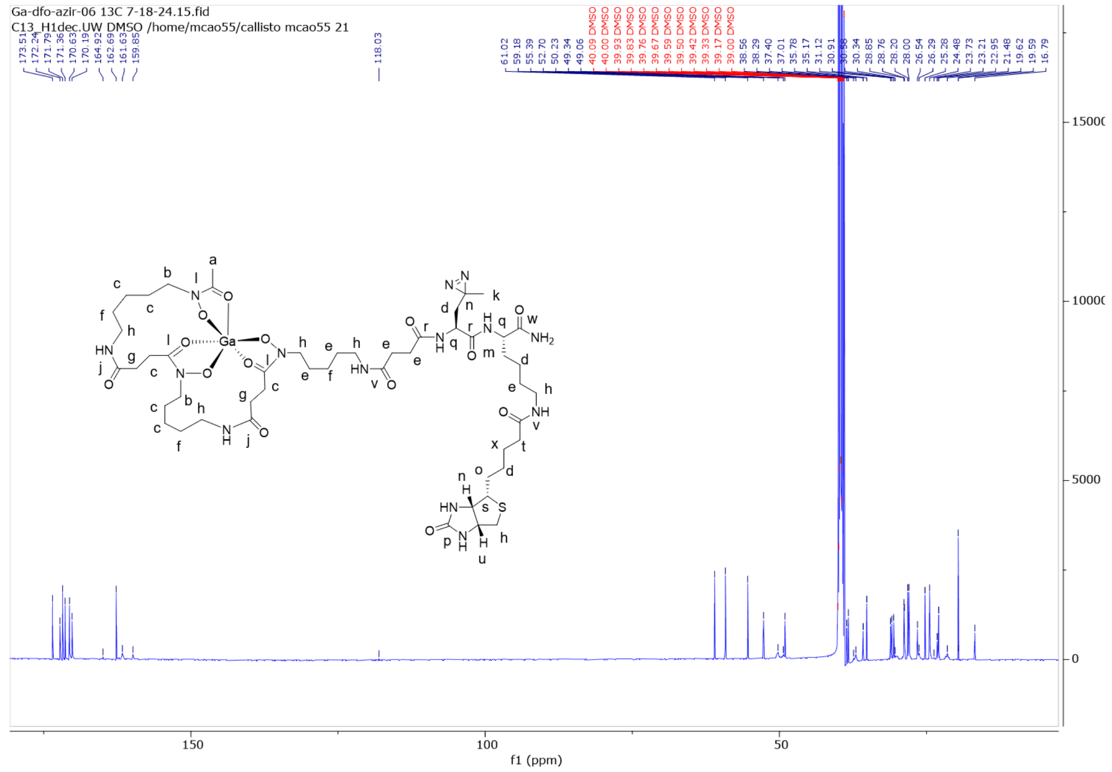

Figure S40. ^{13}C-NMR spectrum of **Ga-DFO-azir-06**. 500 MHz, DMSO-d_6.

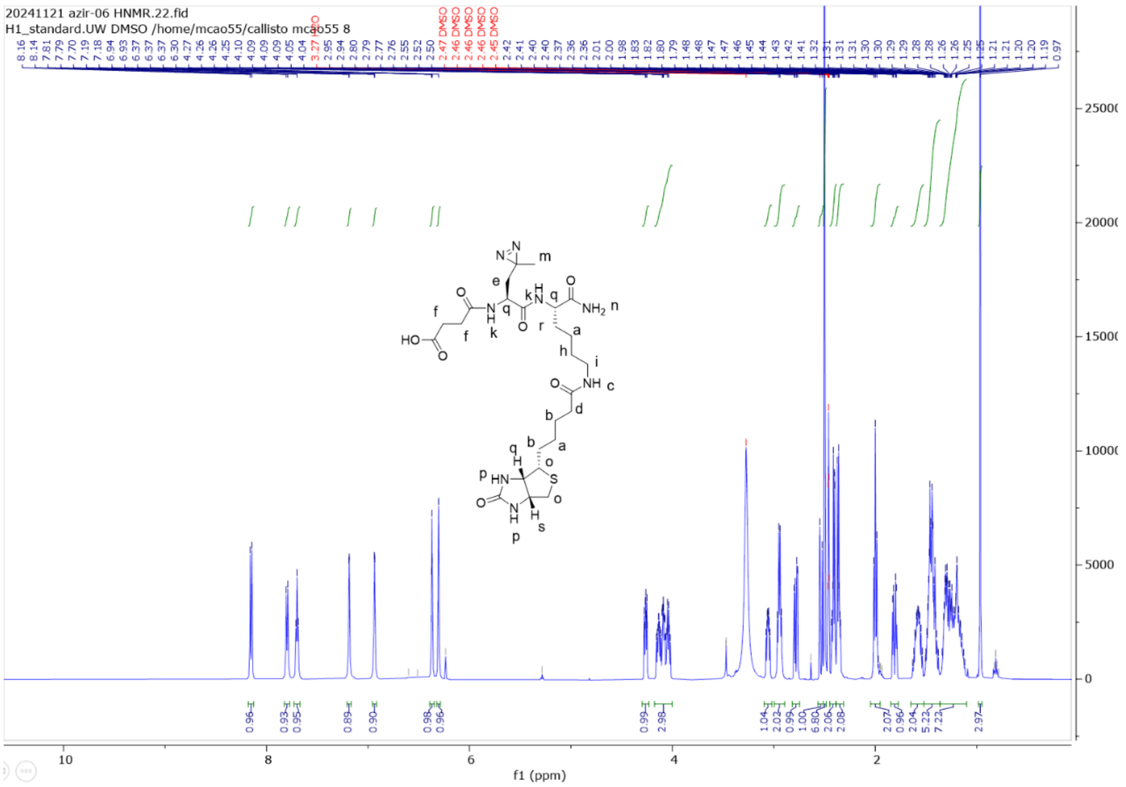

Figure S41. ^{1}H-NMR spectrum of **azir-biotin**. 500 MHz, DMSO-d_6.

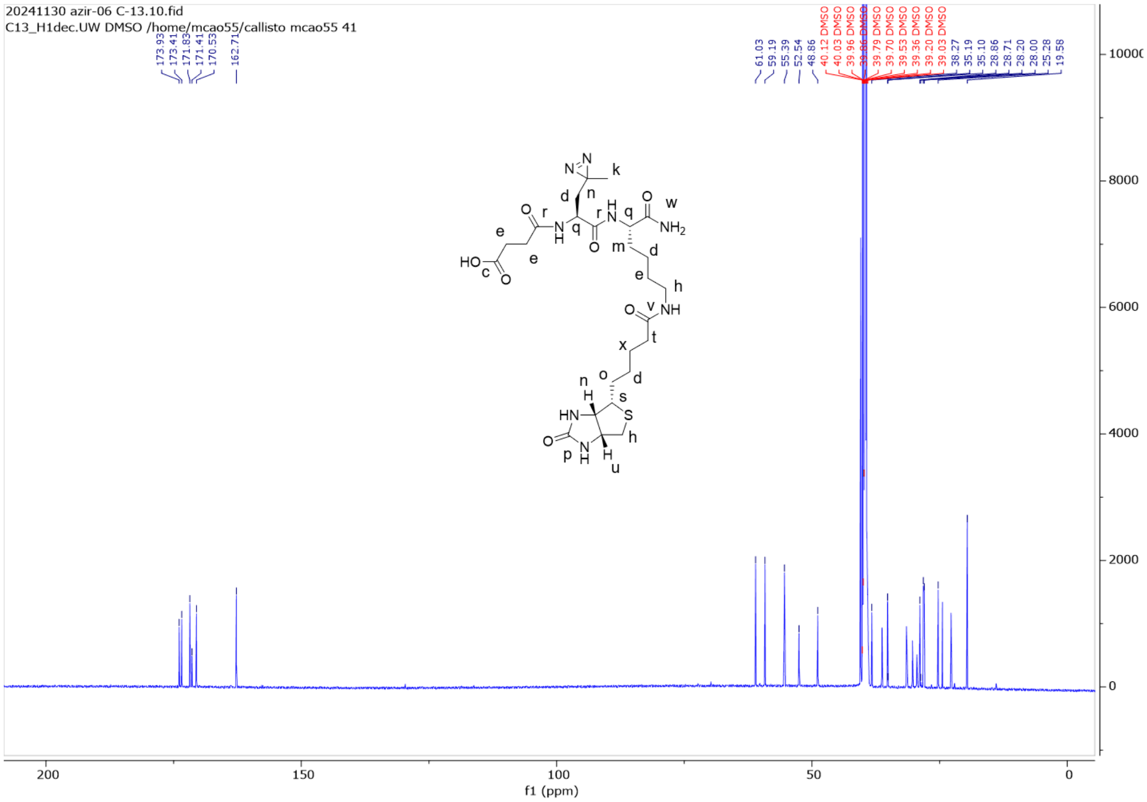

Figure S42. ^{13}C-NMR spectrum of **azir-biotin**. 500 MHz, DMSO-d_6.

1.4 Quantum yields

Quantum yield for each complex and corresponding ligands were determined using the following equation:

$$\Phi_x = \Phi_S \frac{\text{Gradient}_x}{\text{Gradient}_S}$$

Where "S" refers to the organic coumarin fluorophore standard (7-(diethylamino)coumarin-3-carboxylic acid, Φ = 0.50 for **M-DFO-azir-04**; coumarin 343, Φ = 0.62 for **M-DFO-azir-05**) and "x" is the unknown. The gradient is the slope of the graph plotting integrated emission intensity against peak absorption value for a range of concentrations. The excitation wavelength for each complex and corresponding ligand was their corresponding λmax (7-(diethylamino)coumarin-3-carboxylic acid: Ex = 415 nm, Em = 465 nm; coumarin 343: Ex = 444 nm, Em = 480 nm). All samples were measured in ethanol.

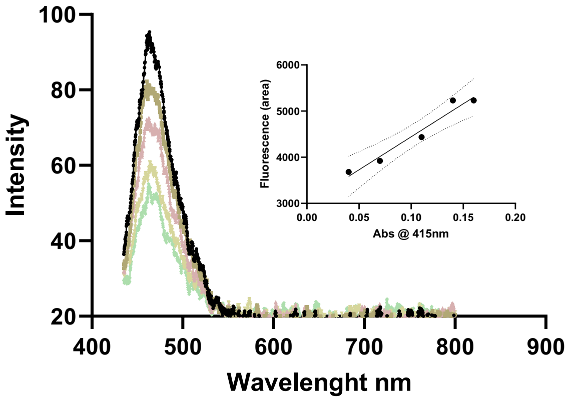

Figure S43. 7-(Diethylamino)coumarin-3-carboxylic Φ = 0.5

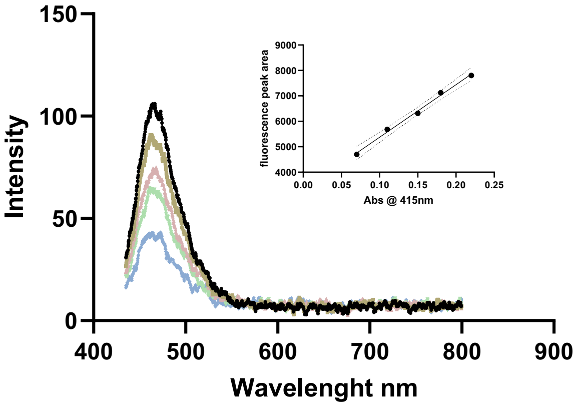

Figure S44. DFO-azir-04 Φ = 0.8

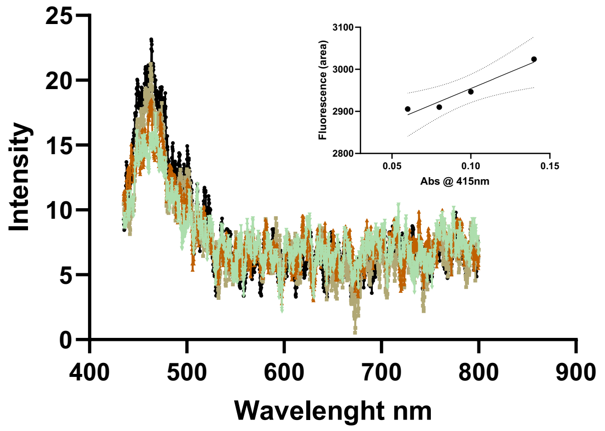

Figure S45. Fe-DFO-azir-04 Φ =0.08

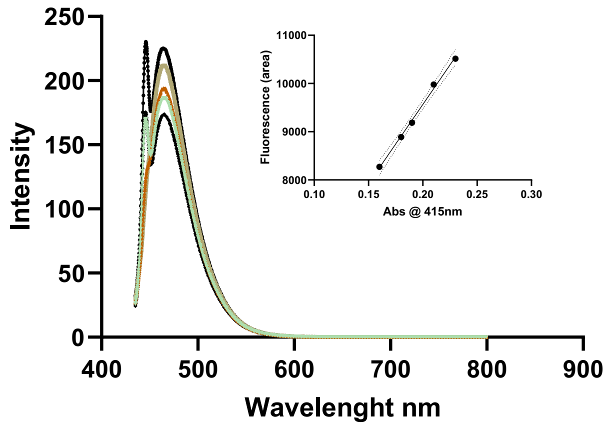

Figure S46. Ga-DFO-azir-04 Φ = 1

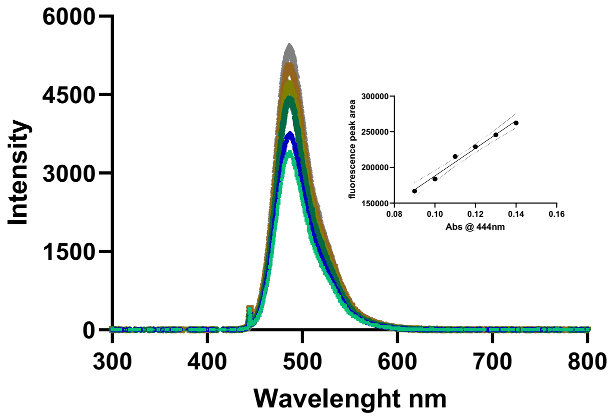

Figure S47. Coumarin 343 Φ = 0.63

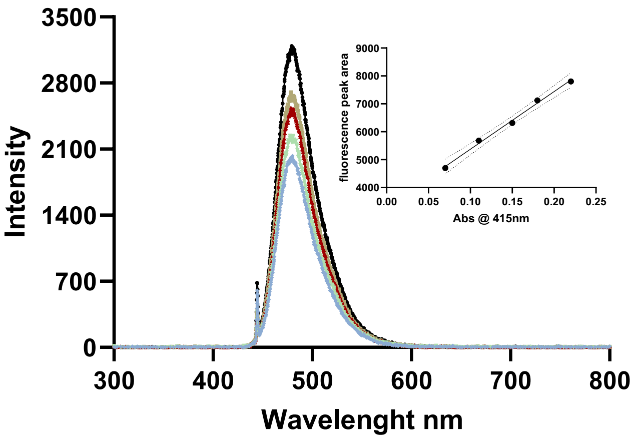

Figure S48. Ga-DFO-azir-05 Φ =0.48

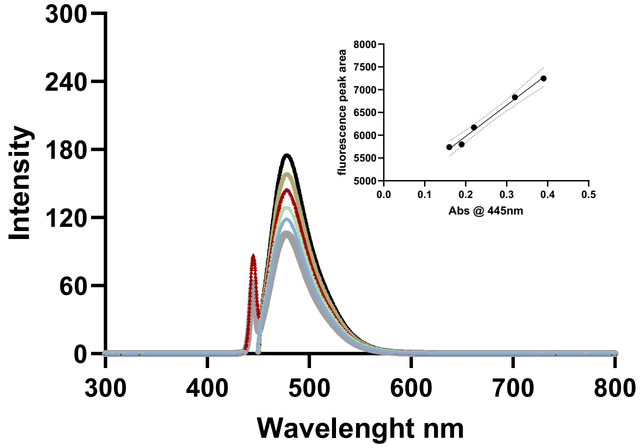

Figure S49. Fe-DFO-azir-05 Φ = 0.06

2. Co-crystal structures

Crystallization and structure determination.

For crystallization, FoxA (10 mg/ml) was mixed with 3-4 fold excess ligand (Fe-D1 or Fe-DFO-azir-01) and incubated on ice for approximately 30 min. Crystals of FoxA-Fe-D1 complex were grown in 1.6-2.0 M ammonium sulfate, 0.1 M HEPES pH 7, 0.6% β-octyl glucopyranoside (β-OG). For crystallization of FoxA-Fe-DFO-azir-01 complex the sample was irradiated with a UV-lamp (365 nm) for 15 min on ice and crystals were grown in 2.0 M ammonium sulphate, 0.1 M Tris pH 8.5 (or 0.1M BICINE pH 9.0), 0.4-0.8% β-octyl glucopyranoside. All crystals appeared after 3-5 days and grew to 20-100 µm maximum size.

X-ray diffraction data were collected at the P14 beamline EMBL, Hamburg. All data were processed with XDS[5] and reduced with AIMLESS[6]. Crystals belonged to the $P3_221$ space group as in the previously determined FoxA-ferrioxamine B structure[7]. The structures of the complexes were solved using molecular replacement in Phaser[8] using apo *FoxA* (pdb: 6I96) as a search model and refined using REFMAC5[9, 10] to 2.4 – 2.7 Å resolution. Coordinates have been deposited to the Protein Data Bank (pdb: 9TU2; 9TUG).

Table S1. X-ray data collection and refinement statistics

Model	*FoxA* + Fe-D1	*FoxA* + Fe-DFO-azir 01
PDBID	pdb_00009TU2	pdb_00009TUG
Beamline	PETRA III P13	PETRA III P13
Wavelength (Å)	0.984	0.984
Space group	P3221	P3221
Cell dimensions		
a, b, c (Å)	95.083, 95.083, 177.280	95.70, 95.70, 178.28
α, β, γ (°)	90.0, 90.0, 120.0	90, 90, 120
Resolution (Å)	82.344–2.424 (2.724–2.424)	82.9–2.64 (2.84–2.64)
Rpim	0.047 (0.457)	0.029 (0.449)
Rmeas	0.200 (1.881)	0.114 (1.759)
I / σI	12.0 (1.8)	14.9 (1.6)
CC1/2	0.996 (0.785)	0.998 (0.710)
Completeness (%) spherical/elliptical	63.0 / 94.5	53.9 / 91.8
Redundancy	18.3 (16.6)	16.1 (15.1)
**Refinement**		
Resolution (Å)	82.6–2.47	82.9–2.64
No. reflections	26167 (343)	15353 (770)
Rwork / Rfree	0.221 / 0.261	0.246 / 0.292
No. atoms	5517	5364
Protein	5312	5268
Ligand/ion	205	96
B-factors (Å²)		
Protein	58.7	76.5
Ligand/ion	92.7	94.1
R.m.s. deviations		
Bond lengths (Å)	0.016	0.013

Bond angles (°)	1.86	2.07
Ramachandran (%)		
Favored regions	95.83	92.65
Allowed regions	3.43	6.75
Outliers	0.75	0.60
MolProbity score	7.47	27.3

*Values in parentheses are for highest-resolution shell.

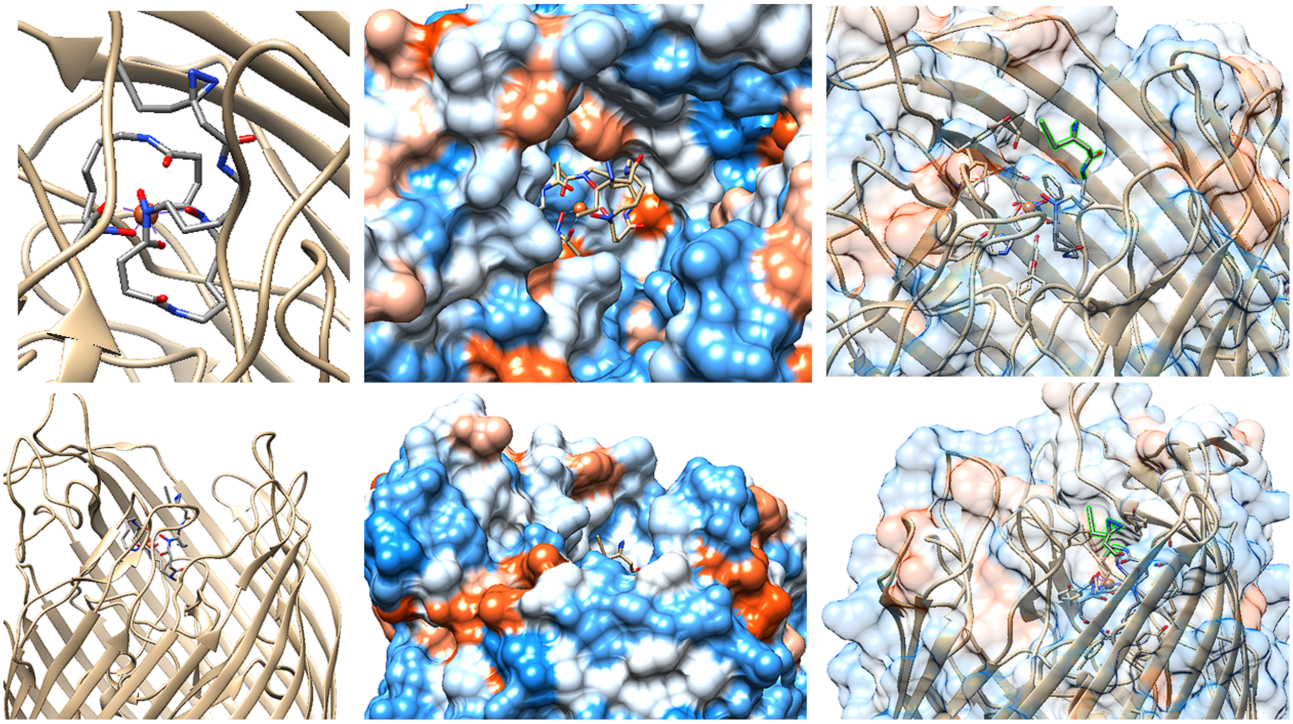

Figure S50. Co-crystal structure of FoxA in complex with **Fe-DFO-azir-01**. The diazirine-alkyne handle of **Fe-DFO-azir-01** is highlighted in green.

3. Computational experiments

Ligand parameters

Force field parameters for **M-DFO-azir-02** were generated using the second-generation General Amber Force Field (GAFF2).[11] Ligand geometries were first optimized in Gaussian 16 (Gaussian 16 Rev. C.01 (Wallingford, CT, 2016)) using the B3LYP functional,[12, 13] with Grimme's D3BJ dispersion correction,[14] together with the polarizable continuum model[15] (PCM) for the solvation. Electrostatic potential (ESP) calculations were then carried out at the Hartree–Fock (HF) level with the 6-31G* basis set in the gas phase. Finally, the partial charges were fitted using the RESP module in AmberTools23 (Amber 2023, University of California: San Francisco, 2023).

MD Simulations

The systems were constructed using the CHARMM-GUI membrane builder.[16] The initial model was derived from the crystal structure of P. aeruginosa FoxA (PDB ID 6I96). The ligand, **M-DFO-azir-02**, was positioned by aligning its desferrioxamine moiety with that of Ferrioxamine B in the crystal structure. The complex of FoxA and ligand was then embedded in an asymmetric bilayer composed of lipopolysaccharides (LPS) in the outer leaflet and phospholipids (PL) in the inner leaflet. For the PL layer, a composition of PE:PG:CL (15:4:1) was used. All systems were solvated with TIP3P water molecules. Both Mg^{2+} and Ca^{2+} ions were added to neutralize the LPS molecules, while 0.15 M NaCl was used to neutralize the whole system.

Simulations were performed using Gromacs 2022.5. The Amber14sb force field was used for the protein, Lipid14 was used for lipids, and GAFF2 force field was applied for the ligand. Long-range electrostatics were calculated using the particle mesh Ewald method(PME). A 12 Å cut-off for Lennard–Jones interactions was used along with a force-based switching function starting at 10 Å. All simulations were performed in the NPT ensemble at p = 1 bar and T = 310K. The pressure was maintained using Parrinello−Rahman barostat and the temperature was maintained by velocity-rescaled thermostat. The LINCS algorithm was used to constrain the bond vibrations involving hydrogen atoms, and a time step of 2 fs was adopted. Before production runs, the system was minimized in energy, heated to 310K and pre-equilibrated in the NVT ensemble with stepwise releases of harmonically restraints on heavy atoms. Simulations were then subjected to production runs of 1μs, and the last 500-ns trajectories were used for analysis.

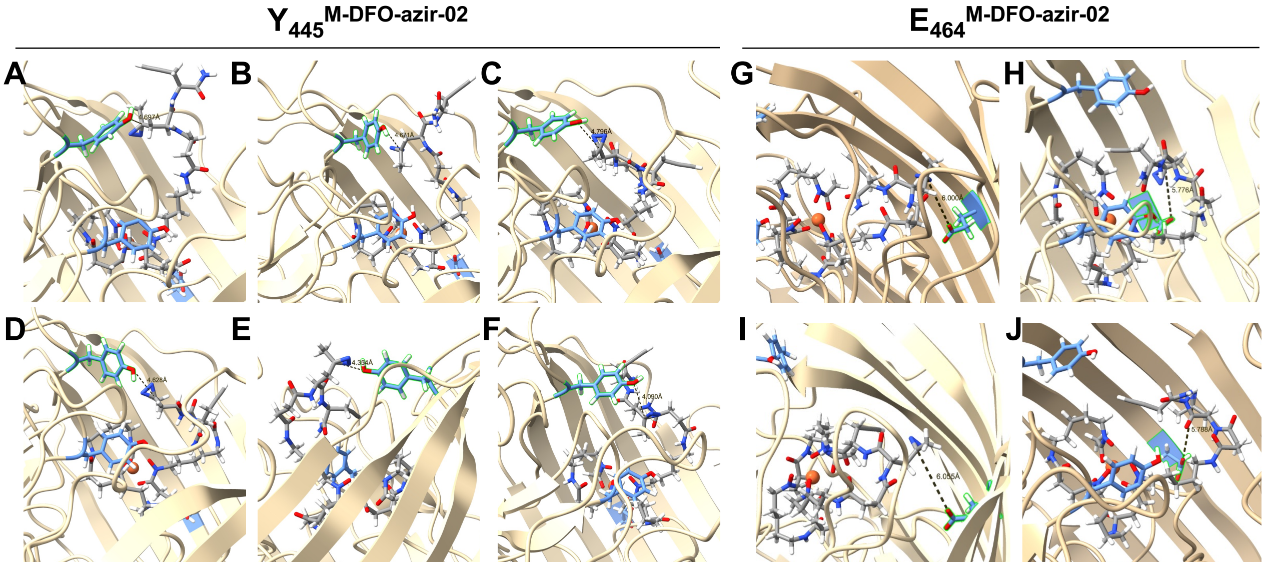

Figure S51. Various computational models of **M-DFO-azir-02** show the diazirine in close proximity to Y445 (A-F) and E464 (G-J) whilst the M[DFO] moiety sits within the binding pocket of FoxA. Models A, B and F show the alkyne moiety outside of the beta-barrel and possibly available for conjugation through CuAAC.

4. Amino acids reactivity screening

Photo cross-link reaction with single amino acid

Stock solutions of the Ac-x-Ome (X= Tyr, Aps, Gln, Arg, and His 19mM) prepared in acetonitrile or DMSO and **M-DFO-azir-01**,**-02**,**-04**,**-05**, or **-06** (M= apo, Ga, Fe) (0.4 mM) were prepared in 50% acetonitrile in water. **M-DFO-azir-01**,**-02**,**-04**,**-05**, or **-06** (5 µL, 1 eq.) and the amino acid (20 µL, 100 eq.) were added in sequence to a 96 well plate in triplicate and were irradiated with a broadband UV lamp (365 nm LED) over an ice pack for 15 min. Samples were filtered and injected into the LC-MS (method C).

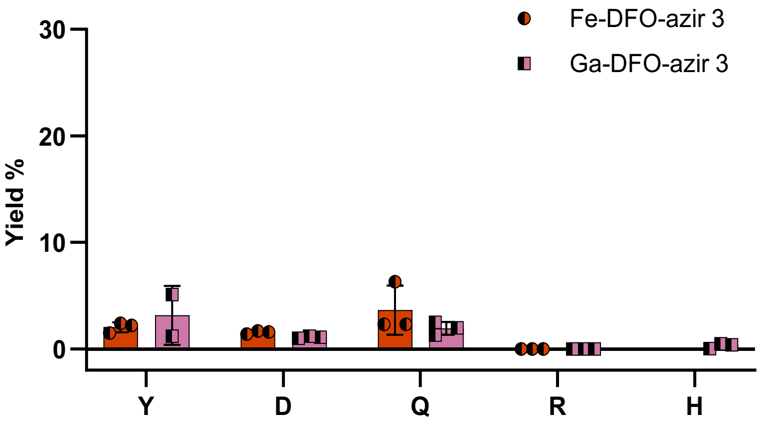

Figure S52. M-DFO-azir-03 reactivity with single Ac-X-Ome amino acids.

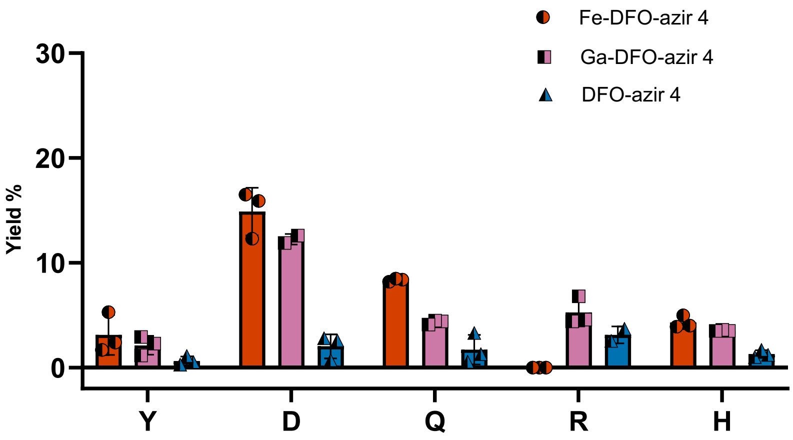

Figure S53. M-DFO-azir-04 reactivity with single Ac-X-Ome amino acids.

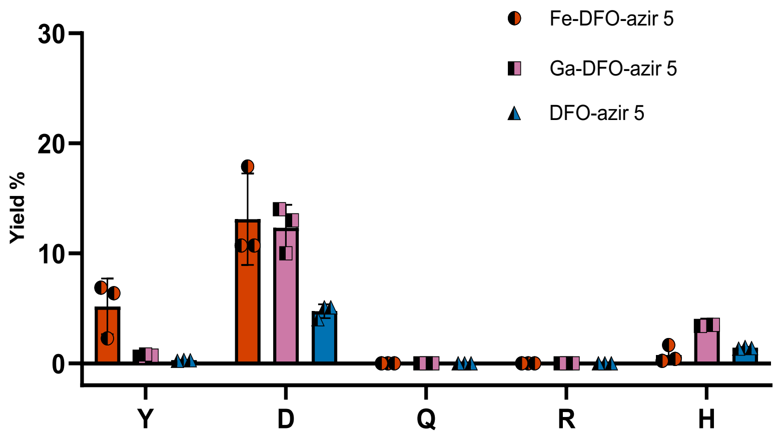

Figure S54. M-DFO-azir-05 reactivity with single Ac-X-Ome amino acids.

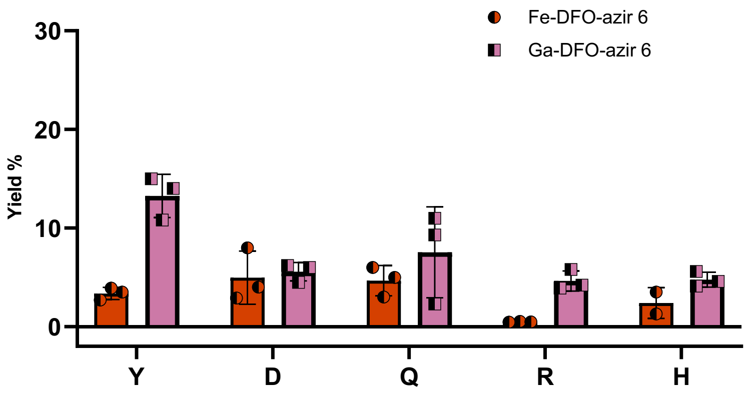

Figure S55. M-DFO-azir-06 reactivity with single Ac-X-Ome amino acids.

5. Biological assays

5.1 General biological methods

Bacteria culture

E. coli (K-12) and P. aeruginosa (PAO1): An overnight culture grown in LB medium was inoculated to OD_{600nm} = 0.01 into fresh iron-deficient LB medium (containing 200 µM bipyridine) and grown until the OD_{600} reached 0.6.

E. coli Lemo21 C43(DE3): *E. coli* Lemo21 (DE3) (New England Biolabs) were transformed as per manufacturer's instructions using 140 ng of FoxA pET28a plasmid (1 µL) and grown on selection LB agar plates containing 25 µg/mL Kanamycin and 35 µg/mL Chloramphenicol at 37 °C for 16 hours. A single colony was gathered and incubated with fresh 10 mL LB medium, 25 µg/mL Kanamycin and 35 µg/mL Chloramphenicol at 37 °C, 210 rpm for 16 hours. This overnight culture was inoculated to OD_{600nm} = 0.01 in 400 mL iron-deficient LB medium (containing 200 µM bipyridine), 25 µg/mL Kanamycin and 35 µg/mL Chloramphenicol at 37 °C, 210 rpm until OD_{600nm} reached 0.6. The bacterial culture was cooled, induced with 0.1 mM IPTG for protein expression, and incubated for 16 hours at 20 °C.[7]

SDS-PAGE

Protein samples were mixed with loading buffer (5% β-mercaptoethanol in either 2X Laemmli buffer or 5X loading buffer containing 5% SDS, 50% glycerol, 0.1% bromophenol blue, 250 mM Tris-HCl pH 6.8) at a 1:1 or 1:5 sample-to-buffer ratio and heated to 95 °C for 3 minutes. The samples were then subjected to electrophoresis using either 8–16% or 4–20% Mini-PROTEAN® TGX Stain-Free™ Protein Gels (#4568103, #4561093 or #4561094) at 220 volts for 30 minutes or 130 volts in 1 hour in 1X TGS buffer (Tris/Glycine/SDS, #1610732).

Precision Plus Protein™ All Blue Prestained Protein Standards (250-10 kDa, #1610373) or PageRuler™ Prestained Protein Ladder (180-10 kDa, #26616) were used as molecular weight markers. Following electrophoresis, the gels were fixed in a solution of 30% ethanol and 10% acetic acid in water, washed with DI water for 10 minutes, and then imaged using a Typhoon 9400 Variable Mode Imager for fluorescence or stained with Coomassie R-250. For enrichment experiments, the gels were rinsed, fixed and stained with either Coomassie R-250 or Pierce™ Silver Stain kit (#24612) according to manufacturer's instructions.

Western blot

The gel was transferred to a nitrocellulose membrane (45 µm, 8.5 x 13.5 cm, Bio-Rad, CAS# 1620167) in 20% methanol TGX 1x buffer for 1 hour at 100 volts, at 4 °C. Protein transfer was verified by Ponceau S staining for 10 minutes at room temperature. The stain was removed by washing five times with TBST buffer until the stain disappeared. The membrane was blocked with 5% BSA (TBST, 0.03% azide) for 30 minutes at room temperature. After washing three times for 5 minutes each with TBST, the membrane was incubated with primary antibody (Penta-His Antibody, Mouse IgG1, QIAGEN, CAS#: 34660) with mild stirring for 16 hours at 4 °C. Following three washes of 5 minutes each with TBST, the membrane was incubated with secondary antibody (Rabbit anti-Mouse IgG (H+L) Cross-Adsorbed Secondary Antibody, DyLight™ 800, Invitrogen™, CAS# PISA510164) for 1 hour at room temperature. After three final washes of 5 minutes each with TBST, the membrane was imaged using an Invitrogen™ iBright™ FL1500 Imaging System.

5.2 Protein covalent tagging

5.2.1 FoxA purification

Bacterial culture and lysis

A 100 mL bacterial culture with an OD_{600nm} of 0.6 was pelleted by centrifugation at 4 °C, 4000 g for 15 minutes and resuspended in 5 mL of lysis buffer 1 (30 mM Tris, pH 7.4, 225 mM NaCl, 1% v/v Triton X-100, 1% w/v OG, 10% v/v glycerol) a final concentration of 2 mM of PMSF. Cells were lysed by sonication on ice using a Fisher Scientific Model 505 Sonic Dismembrator CL-18 with 1s bursts and a 4s off interval at 70-80% intensity for a total of 8 minutes of sonication. The lysate was incubated overnight at 4 °C on a rotary shaker. Insoluble material was removed by centrifugation at 4 °C, 30 000 g for 30 minutes. The first supernatant was set aside and the pellet was resuspended in lysis buffer 2 (30 mM Tris, pH 7.4, 225 mM NaCl, 5% v/v Triton X-100, 5% w/v OG, 10% v/v glycerol) and sonicated as described above for total of 1min. Insoluble material was once more removed by centrifugation at 4 °C, 30 000 g for 30 minutes. The two supernatants were combined for affinity chromatography.

Affinity column chromatography

A Ni-Sepharose 6 Fast Flow histidine-tagged protein purification resin was used for the purification of *FoxA*. 1 ml of resin was equilibrated with 20 column volumes (CV) of 25 mM imidazole in lysis buffer 1. The equilibrated resin was added to the lysate and incubated for 1 hour at 4 °C on a rotary shaker. The flow-through was reloaded onto the column twice, and the column was washed with 20 CV of lysis buffer containing 25 mM imidazole. The protein was eluted with 250 mM imidazole in lysis buffer 1 in five 300

μL fractions. The fractions were analyzed by SDS-PAGE to identify the protein-containing fractions followed by western blot. The protein was solvent exchange to lysis buffer 1 and protein concentration was quantified by BCA assay.

5.2.2 BSA and FoxA covalent tagging

Photo cross-link reaction with BSA and FoxA

BSA was prepared in DPBS (0.4% C8E4), and FoxA was prepared in DPBS (0.4% C8E4). M-DFO-azir-0x were dissolved in water for metal complexes or DMSO for the apo-ligand. For each reaction, 7.5 μL of probe (0.4 mM, 100 eq) and 7.5 μL of either BSA or FoxA (0.4 mg/mL, 1 eq) were sequentially added to a 96-well plate, followed by the addition of 15 μL of DPBS buffer. FoxA samples were incubated in the dark at room temperature for 30 minutes. Subsequently, they were photoirradiated with a 365 nm UV lamp over an ice pack for 15 minutes. The samples were then transferred to small conical vials and washed twice with 15 μL of DPBS buffer. BSA samples were precipitated with acetone at -80 °C three times and then centrifuged at 30 000 g for 10 minutes to remove excess unreacted probe. Pellets were resolubilized in water. The BSA conjugation was analyzed by MALDI and western blot. *FoxA* samples were confirmed by western blot and submitted directly for MS/MS analysis without further treatment.

Fluorophore conjugation

Photo cross-linked labeled BSA or FoxA was dissolved in DPBS (pH 7.3, 25 mM, 1% SDS, 1% Triton-X). For the CuAAC reaction, the following cocktail was added: Azide Fluor 545 (10 mM, 2 μL), a premixed solution of $CuSO_4$ (50 mM, 2 μL) and TBTA (1.67 mM, 6 μL), and tris(2-carboxyethyl)phosphine (TCEP, 52 mM, 2 μL). The reaction mixture was stirred overnight in the dark at room temperature.

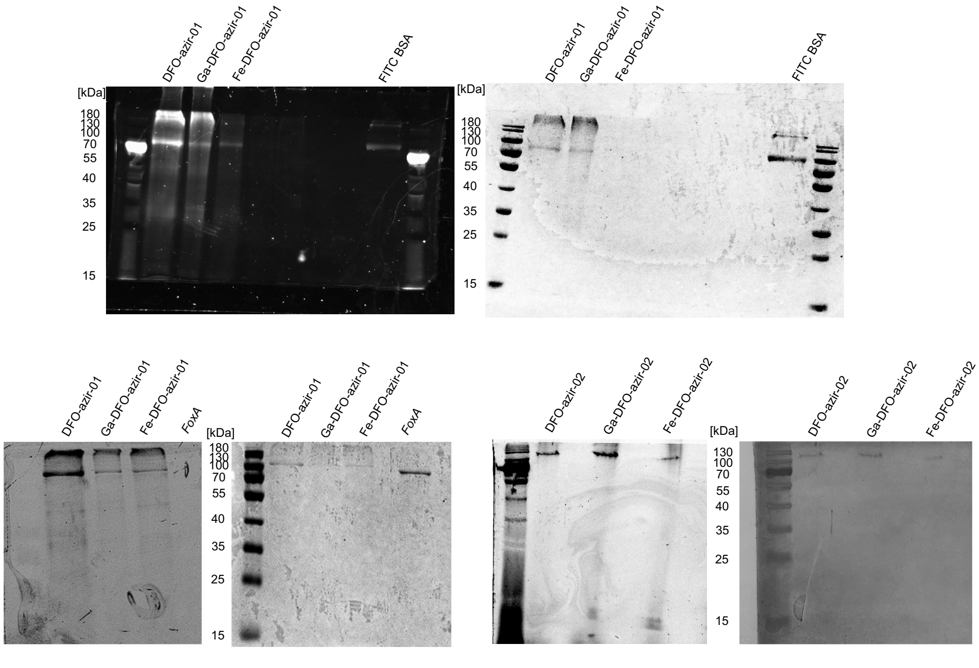

Figure S56. Top panel: Purified BSA protein tagged with **M-DFO-azir-01** and conjugated to Azide Fluor 545 were visualized by gel-based fluorescent imaging (left panel) and total protein staining (right panel). Botton panel: Purified FoxA protein tagged with **M-DFO-azir-01** and **M-DFO-azir-02** and conjugated to Azide Fluor 545 were visualized by gel-based fluorescent imaging (left panel) and total protein staining (right panel).

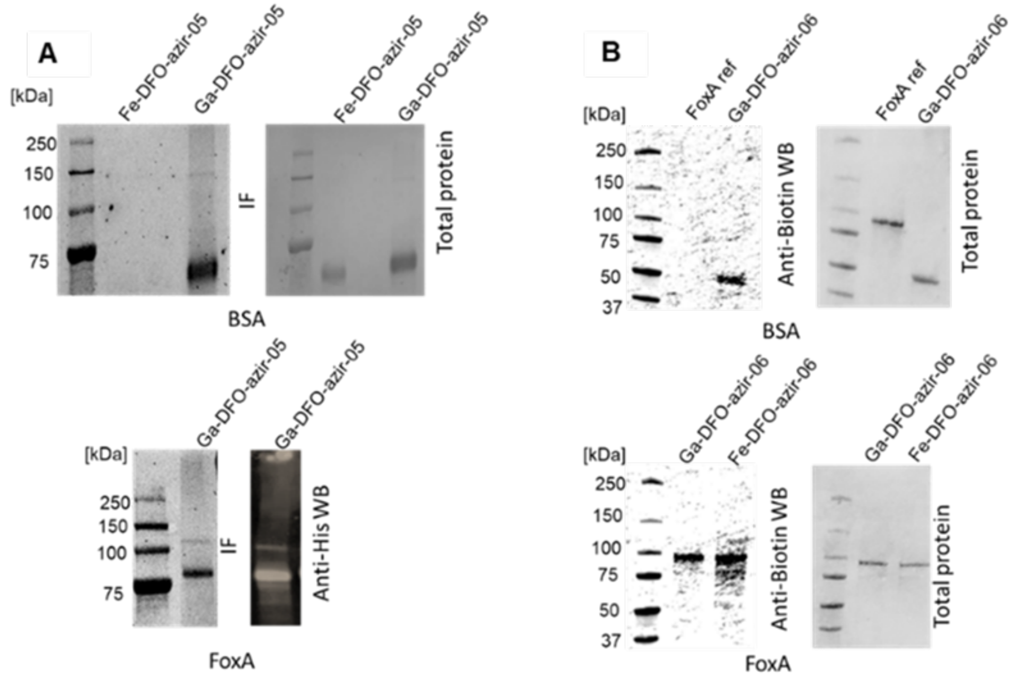

Figure S57. M-DFO-azir-05 (A) and **M-DFO-azir-06 (B)** labeling of BSA and FoxA in vitro. Photo-irradiation (15 min) was performed prior to a 30-minute incubation for FoxA and with no incubation for BSA.

5.3 Bacterial lysate and live cell labeling

Bacterial lysate labeling

The lysate was obtained as described in the *FoxA* purification protocol. Sample concentration was determined using a nanodrop. The control probe **MP01** (final concentration 5 µM) was added to 100 µL of bacterial cell lysate and exposed to 365 nm UV light for 15 minutes on ice. For CuAAC, the following cocktail was added to the reaction mixture: Azide Fluor 545 (10 mM, 2 µL), a premixed solution of CuSO4 (50 mM, 2 µL) and TBTA (1.67 mM, 6 µL), and tris(2-carboxyethyl) phosphine (TCEP, 52 mM, 2 µL). The reaction mixture was incubated at 45°C in the dark while stirring overnight. The reaction was quenched by adding an equal volume of loading buffer (5% β-mercaptoethanol in 2X Laemmli). 15 µL of the sample were loaded per lane on the gel (gel conditions as previously described).

Live cell labeling

The bacterial pellet was obtained by centrifugation (50 mL, 4000 g, 15 min) and washed with 50 mL DPBS (4 °C, 4000 g, 15 min). The cell pellet was resuspended in 5 mL DPBS, and 1 mL was transferred to a 6-well plate. The **M-DFO-azir-0x** probe (final concentration 25 µM) was added to the resuspended bacterial sample and incubated at 37 °C for 30 min (**M-DFO-azir-01** and **-02**) or 2-4 h (**M-DFO-azir-04**, **-05**, and **-06**) in the dark. The samples were UV-irradiated (365 nm) on ice for 15 min with rocking. Bacteria were transferred to 2 mL Eppendorf tubes, pelleted, and washed three times with 500 µL DPBS buffer. Cell lysate was prepared as described in the FoxA purification protocol. For **M-DFO-azir-01** and **-02** the CuAAC reaction was performed as mentioned previously.

Competition labeling with Fe-DFO

The bacterial culture was grown and prepared as previously described. To each well, increasing concentrations of Fe-DFO were added (0 eq., 1 eq. 25 µM, 10 eq. 250 µM, and 100 eq. 2.5 mM), followed

by the addition of probes **M-DFO-azir-05** and **M-DFO-azir-06** (final concentration 25 µM) to the resuspended bacterial samples. These samples were then incubated at 37 °C for 4 h in the dark. Following incubation, the samples were transferred to 2 mL Eppendorf tubes, pelleted, and washed three times with 500 µL DPBS buffer. The cells were lysed with 5% SDS lysis buffer (30 mM Tris, pH 7.4, 225 mM NaCl, 5% w/v SDS, 10% v/v glycerol) and a final concentration of 2 mM of PMSF by heating at 95 °C for 6 min. Protein concentration was quantified by BCA assay and normalized. SDS-PAGE, as described above, was used to separate the proteins, which were then visualized using a Typhoon 9400 Variable Mode Imager for fluorescence or stained with Coomassie R-250.

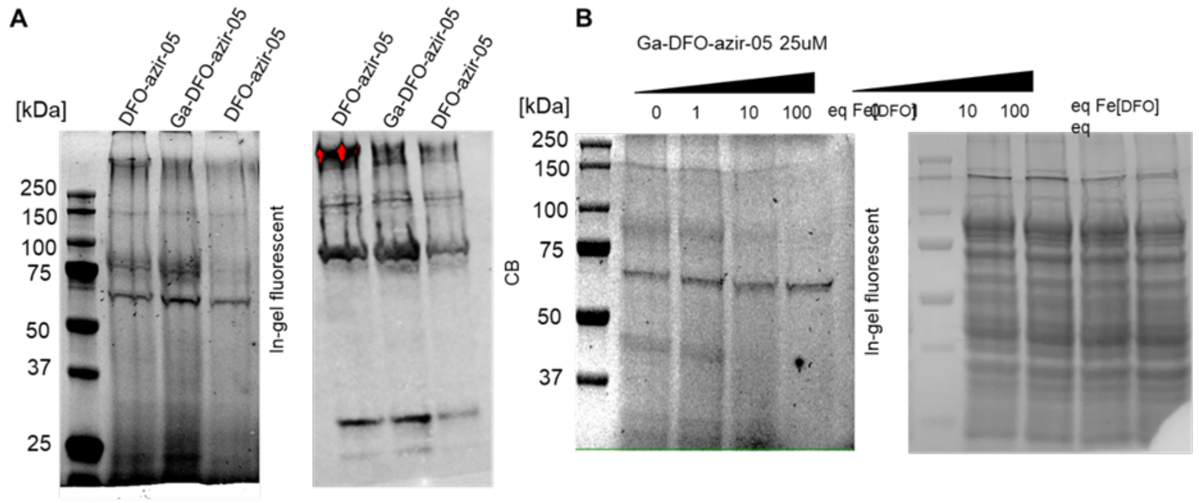

Figure S58. (A) In-gel fluorescence of **Ga-DFO-azir-05** and **DFO-azir-05** (25 µM) in *E.coli* Lemo21 cell lysate prior 4h incubation. **(B)** Challenge displacement experiment where **Ga-DFO-azir-05** (25 µM) was co-incubated with increasing amount of Fe-DFO for 4h in *E.coli* Lemo21 cells. CB= Coomassie blue stain.

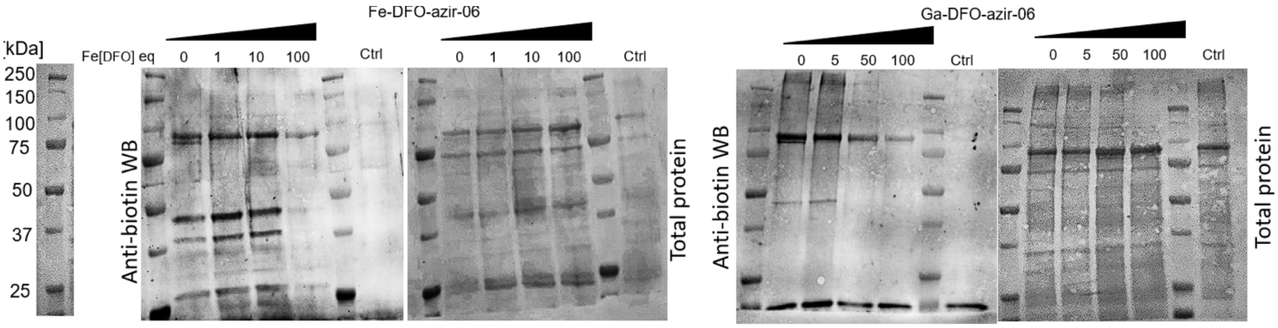

Figure S59. A challenge displacement experiment was conducted to investigate the interaction between **Fe-** and **Ga-DFO-azir-06** (25 µM) in the presence of increasing concentrations of Fe-DFO. A negative control (Ctrl) lacking the probe was also included. All samples were incubated for 4 hours in *E. coli* Lemo21 cells under iron-normal media conditions.

5.4 Enrichment experiments

5.4.1 Enrichment in E. coli Lemo21 (DE3) cells

Bacterial cultures were transformed and grown as described in the general biological methods. 400 mL of bacterial culture at an OD_{600nm} of 0.6 was washed with 400 mL PBS and resuspended in 8 mL PBS. The suspension was distributed into a 12-well plate (1 mL per well). The probe was added to a concentration of 125 µM to the bacterial solution. Bacteria were incubated with the probe for 4 hours at 37 °C, 145 rpm,

protected from light. The plate was irradiated at 365 nm for 15 minutes on ice. Samples were gathered, pelleted and washed three times with 1 mL PBS. Pellets were resuspended in lysis buffer (30 mM Tris, pH 7.6, 225 mM NaCl, 1% w/v Triton X-100, 1% w/v OG, 10% w/v glycerol; 10 mL per 1 g of pellet) containing 2 mM PMSF. Cells were lysed using a fine-tip sonicator with cycles of alternating 1-second pulses on and 1-second pulses off for a total of 3 minutes on and 3 minutes off. Lysates were incubated on a rotary shaker for 20 hours at 4 °C. Subsequently, samples were centrifuged at 30,000 g for 30 min at 4 °C to separate the supernatant (SN1) from the pellet. Pellets were resuspended in 500 µL of 5% Triton-X-100, 5% OG lysis buffer (30 mM Tris, pH 7.6, 225 mM NaCl, 5% w/v Triton X-100, 5% w/v OG, 10% w/v glycerol), sonicated for 1 min, and incubated on a rotary shaker for 20 hours at 4 °C. Samples were centrifuged at 14,100 g for 10 min at 20 °C to separate the supernatant (SN2) and debris. Supernatants SN1 and SN2 were combined and protein concentration was determined by Bradford assay using Pierce™ BCA protein assay kit (#23227). 200 µL of Pierce™ high-capacity streptavidin agarose (#20353) was equilibrated with lysis buffer (3 × 500 µL) and incubated with the combined supernatants SN1 and SN2 for 2 hours at room temperature on a rotary shaker. The resin was washed three times with 1 mL lysis buffer, with a 10 min incubation on a rotary shaker for each wash. The first and second elution (E1 and E2) was achieved by adding 50 µL of elution buffer (4% SDS, 50 mM Tris, 5 mM EDTA, 25% glycerol, 5% β-mercaptoethanol, pH 6.8), inverting a few times at room temperature. The third (E3) elution was achieved by adding 50 µL of elution buffer (4% SDS, 50 mM Tris, 5 mM EDTA, 25% glycerol, 5% β-mercaptoethanol, pH 6.8) and heating at 95 °C for 3 minutes. Elutions were run by SDS-PAGE and stained with Coomassie before analysis of bands by MS/MS.

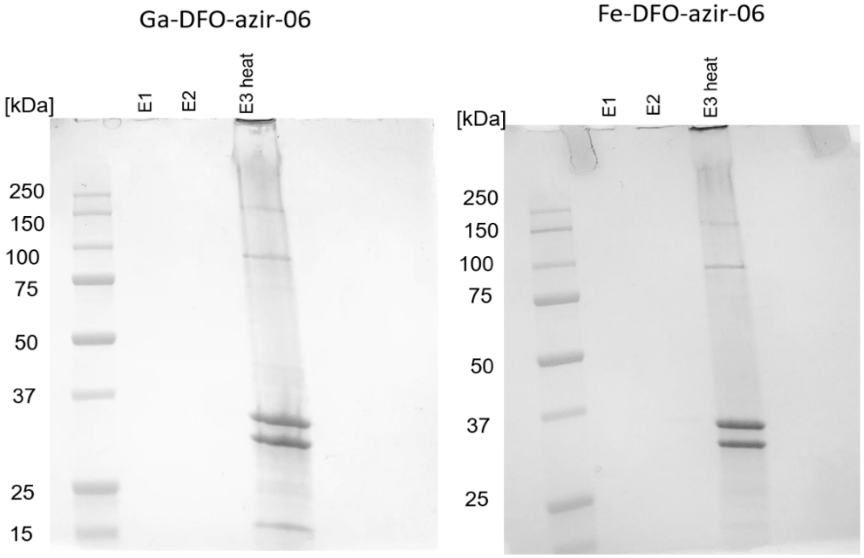

Figure S60. Enrichment of biotinylated proteins from *E. coli* Lemo21 lysate using streptavidin affinity purification in SDS-PAGE with Coomassie staining.

5.4.2 Enrichment in P. aeruginosa PAO1 and E. coli K-12 cells

Bacterial cultures were grown as described in the general biological methods. 450 mL of bacterial culture at an OD_{600nm} of 0.6 was washed with 450 mL PBS and resuspended in 9 mL PBS. The suspension was distributed into a 12-well plate (1 mL per well). The probe was added to a concentration of 125 µM to the bacterial solution. Bacteria were incubated with the probe for 4 hours at 37 °C, 145 rpm, protected from light. The plate was irradiated at 365 nm for 15 minutes on ice. Samples were gathered, pelleted and washed three times with 1 mL PBS. Pellets were resuspended in lysis buffer (30 mM Tris, pH 7.6, 225 mM NaCl, 1% w/v Triton X-100, 1% w/v OG, 10% w/v glycerol; 10 mL per 1 g of pellet) containing Roche

cOmplete™, Mini, EDTA-free Protease Inhibitor Cocktail (Sigma-Aldrich, 1183617000). Cells were lysed using a fine-tip sonicator with cycles of alternating 1-second pulses on and 1-second pulses off for a total of 3 minutes on and 3 minutes off. Lysates were incubated on a rotary shaker for 20 hours at 4 °C. Subsequently, samples were centrifuged at 30,000 g for 30 min at 4 °C to separate the supernatant (SN1) from the pellet. Pellets were resuspended in 500 µL of 5% Triton-X-100, 5% OG lysis buffer (30 mM Tris, pH 7.6, 225 mM NaCl, 5% w/v Triton X-100, 5% w/v OG, 10% w/v glycerol) containing Roche cOmplete™, Mini, EDTA-free Protease Inhibitor Cocktail (Sigma-Aldrich, 1183617000), sonicated for 1 min, and incubated on a rotary shaker for 6 hours at 4 °C. Samples were centrifuged at 14,100 g for 10 min at 20 °C to separate the supernatant (SN2) and debris. Supernatants SN1 and SN2 were combined and protein concentration was determined by Bradford assay using Pierce™ BCA protein assay kit (#23227). 200 µL of Pierce™ high-capacity streptavidin agarose slurry (#20353) was equilibrated with lysis buffer (3 × 500 µL) and incubated with combined SN1 and SN2 for each sample. Total protein content was normalized to 2 mg across all samples. Incubation on streptavidin was done at 4 °C for 16 h on a rotary shaker. The resin was washed three times with 1 mL 0.2% SDS in PBS 1X, with a 10 min incubation on a rotary shaker for each wash. Elution of enriched proteins was achieved by adding 100 µL of elution buffer (25 mM biotin, 50 mM Tris, 5% DMSO, pH 8) and heating at 95 °C for 5 minutes. Eluates were submitted as liquid for MS/MS analysis.

6. Tandem mass spectrometry analysis

6.1 Material and methods

Enzymatic "In Gel" Digestion

Coomassie stained gel pieces were de-stained completely in MeOH/H20/NH4HCO3 [50%/50%/100mM], dehydrated for 5 min in ACN/H_20/NH_4HCO_3 [50%/50%/25mM] then once more for 30sec in 100% ACN. Dried in a Speed-Vac for 1 min, reduced in 25mM DTT [Dithiotreitol in 25 mM NH4HCO3] for 15 min at 56°C, alkylated with 55mM CAA [Chloroacetamide in 25mM NH4HCO3] in darkness at room temperature for 15 min, washed once in H20, dehydrated for 2 min in ACN/H20/NH4HCO3 [50%:50%:25mM] then once more for 30sec in 100% ACN. Dried again and rehydrated with 20µl of trypsin solution with 0.01% ProteaseMAX™ surfactant [10ng/µl Trypsin from Promega Corp. in 25mM NH4HCO3/0.01% w/v of ProteaseMAX™ from Promega Corp.]. Let stand for 2min at room temperature then additional 30µl of overlay solution [25mM NH4HCO3/0.01% w/v of ProteaseMAX™] was added to keep gel pieces immersed throughout the digestion. The digestion was conducted for 3hrs at 42°C. Peptides generated from digestion were transferred to a new tube and acidified with 2.5% TFA [trifluoroacetic Acid] to 0.3% final. Gel pieces were additionally extracted with ACN:H2O:TFA [70%:29.25%:0.75%] for 10min while vortexing and solutions combined and dried completely in a Speed-Vac (~20min). Extracted peptides were solubilized in 30µl of 0.05% TFA. Degraded ProteaseMAX™ was removed via centrifugation [max speed, 10minutes] and the peptides solid phase extracted (Pierce® C18 tips, 10µl volume from Thermo Scientific) according to manufacturer protocol. Peptides were eluted off the C18 SPE column with 5µl of acetonitrile/H2O/TFA (70%:30%:0.1%) dried to completion then resolubilized in 20µl total volume for with 0.1% formic acid and 5% Acetonitrile.

NanoLC-MS/MS

Peptides were analyzed on an Orbitrap Fusion™ Lumos™ Tribrid™ platform, where 2µl volume for each sample was injected using Dionex UltiMate™3000 RSLCnano delivery system (ThermoFisher Scientific) equipped with an EASY-Spray™ electrospray source (held at constant 50°C). Chromatography of peptides prior to mass spectral analysis was accomplished using capillary emitter column (PepMap® C18, 2µM, 100Å, 500 x 0.075mm, Thermo Fisher Scientific). NanoHPLC system delivered solvents A: 0.1% (v/v)

formic acid , and B: 80% (v/v) acetonitrile, 0.1% (v/v) formic acid at 0.30 µL/min to load the peptides at 2% (v/v) B, followed by quick 1 minute gradient to 5% (v/v) B and gradual analytical gradient from 5% (v/v) B to 25% (v/v) B over 46 minutes followed by a secondary gradient from 25% (v/v) B to 37.5% (v/v) B over 16 minutes and it concluded with a rapid 5 minute ramp to 95% (v/v) B and a 4 minute flash-out. As peptides eluted from the HPLC-column/electrospray source survey MS scans were acquired in the Orbitrap with a resolution of 120,000 followed by HCD-type MS2 fragmentation into Ion Trap (30% collision energy) under ddMSnScan 1 second cycle time mode with peptides detected in the MS1 scan from 350 to 1600 m/z; redundancy was limited by dynamic exclusion and MIPS filter mode ON.

<u>Data analysis</u>

Raw MS/MS data were converted to mgf file format using MSConvert (ProteoWizard: Open Source Software for Rapid Proteomics Tools Development) for downstream analysis. Resulting mgf files were used to search against Escherichia coli (UP000000625; 4,521 entries) or Pseudomonas aeruginosa (UP000002438; 5,681 entries) Uniprot reference proteome database along with a list of common lab contaminants (172 total entries) using in-house Mascot search engine 2.7.0 [Matrix Science] with variable Methionine oxidation, Asparagine and Glutamine deamidation plus fixed cysteine Carbamidomethylation. A user defined metal-coordinated or no metal containing versions of DFO-azir-0x conjugated probe on aspartic acid, glutamic acid and tyrosine were also considered as variable modification. Peptide mass tolerance was set at 10 ppm and fragment mass at 0.6 Da. Protein annotations, significance of identification and spectral based quantification were done with help of Scaffold software (version 5.0.1, Proteome Software Inc., Portland, OR). Peptide identifications were accepted if they could be established at greater than 80.0% probability by the Peptide Prophet algorithm (Keller, A et al Anal. Chem. 2002;74(20):5383-92) with Scaffold delta-mass correction. Protein identifications were accepted if they could be established at greater than 95.0% probability and contained at least 2 identified peptides. Protein probabilities were assigned by the Protein Prophet algorithm (Nesvizhskii, Al et al Anal. Chem. 2003;75(17):4646-58). Proteins that contained similar peptides and could not be differentiated based on MS/MS analysis alone were grouped to satisfy the principles of parsimony. Proteins sharing significant peptide evidence were grouped into clusters.

<u>Enzymatic "In Liquid" Digestion</u>

IP eluates (~90µl) were diluted with 110µl of MilliQ water, 110µl of TCA and 800µl of acetone [10% TCA and 50% acetone vol:vol final concentration] to facilitate protein precipitation while incubating on ice for 30minutes. The precipitated proteins were collected by centrifugation for 10 minutes at room temperature with max speed (16,000xg) then washed twice with cold acetone while supernatants were discarded and centrifugation steps were kept the same as before. Subsequently protein pellets were air-dried briefly and re-solubilized in 15µl of 8M Urea in 50mM NH_4HCO_3 (pH8.5) overnight at 4ºC. Reduction followed with 2.5µl addition of 25mM DTT plus 42.5µl of 25mM NH_4HCO_3 (pH8.5) and incubation for 15 minutes at 56°C. Samples were cooled on ice to room temperature and 3µl of 55mM CAA (chloroacetaminde) was added for the alkylation step conducted in darkness at room temperature for 15 minutes. Reaction was quenched with 8µl addition of 25mM DTT. Finally, 4µl of Trypsin/LysC solution [100ng/µl 1:1 Trypsin (Promega):LysC (FujiFilm) mix in 25mM NH_4HCO_3] and 25µl of 25mM NH_4HCO_3 (pH8.5) was added to 100µl final volume. Digestion was conducted for overnight at 37°C in a circulating water bath. Reaction was terminated by acidification with 2.5% TFA [Trifluoroacetic Acid] to 0.3% final.

<u>NanoLC-MS/MS</u>

Digests were desalted using Pierce™ C18 SPE pipette tips (100µl volume) per manufacturer protocol and eluted in 20µl of 70/30/0.1% ACN/H_2O/TFA. Dried to completion in the speed-vac and finally reconstituted

in 15µl of 0.1% formic acid containing 2% acetontitrile. Peptides were analyzed by nanoLC-MS/MS using the Agilent 1100 nanoflow system (Agilent) connected to hybrid linear ion trap-orbitrap mass spectrometer (LTQ-Orbitrap Elite™, Thermo Fisher Scientific) equipped with an EASY-Spray™ electrospray source (held at constant 45°C). Chromatography of peptides prior to mass spectral analysis was accomplished using capillary emitter column (PepMap® C18, 3µM, 100Å, 150x0.075mm, Thermo Fisher Scientific) onto which 3µl of extracted peptides was automatically loaded. NanoHPLC system delivered solvents A: 0.1% (v/v) formic acid , and B: 99.9% (v/v) acetonitrile, 0.1% (v/v) formic acid at 0.50 µL/min to load the peptides (over a 30 minute period) and 0.3µl/min to elute peptides directly into the nano-electrospray with gradual gradient from 0% (v/v) B to 30% (v/v) B over 80 minutes and concluded with 5 minute fast gradient from 30% (v/v) B to 50% (v/v) B at which time a 5 minute flash-out from 50-95% (v/v) B took place. As peptides eluted from the HPLC-column/electrospray source survey MS scans were acquired in the Orbitrap with a resolution of 120,000 followed by CID-type MS/MS fragmentation of 30 most intense peptides detected in the MS1 scan from 350 to 1800 m/z; redundancy was limited by dynamic exclusion.

Data analysis

Elite acquired raw MS/MS data files were converted to mgf file format using MSConvert (ProteoWizard: Open Source Software for Rapid Proteomics Tools Development). Resulting mgf files were used to search against *Pseudomonas aeruginosa* database (UP000002438 UniProt reference proteome, 02/22/2021 download containing 5,681 protein entries) along with a cRAP common lab contaminant database (116 total entries) using in-house Mascot search engine 3.0.0 [Matrix Science] with fixed Cysteine carbamidomethylation, variable Methionine oxidation plus Asparagine or Glutamine deamidation plus user defined azir (435.1940Da), DFO-azir-06 (1110.6107Da), Fe-DFO-azir-06 (1163.5222Da) and Ga-DFO-azir-06 (1176.5128Da) as additional modifications on Glutamate, Aspartate or Tyrosine. Peptide mass tolerance was set at 10ppm and fragment mass at 0.6 Da. Protein annotations, significance of identification and spectral based quantification was done with Scaffold software (version 5.3.3, Proteome Software Inc., Portland, OR). Peptide identifications were accepted if they could be established at greater than 11.0% probability to achieve an FDR less than 1.0% by the Peptide Prophet algorithm (Keller, A et al Anal. Chem. 2002;74(20):5383-92) with Scaffold delta-mass correction. Protein identifications were accepted if they could be established at greater than 99.0% probability to achieve an FDR less than 1.0% and contained at least 2 identified peptides. Protein probabilities were assigned by the Protein Prophet algorithm (Nesvizhskii, Al et al Anal. Chem. 2003;75(17):4646-58). Proteins that contained similar peptides and could not be differentiated based on MS/MS analysis alone were grouped to satisfy the principles of parsimony. Proteins sharing significant peptide evidence were grouped into clusters.

6.2 FoxA labeling sites identification

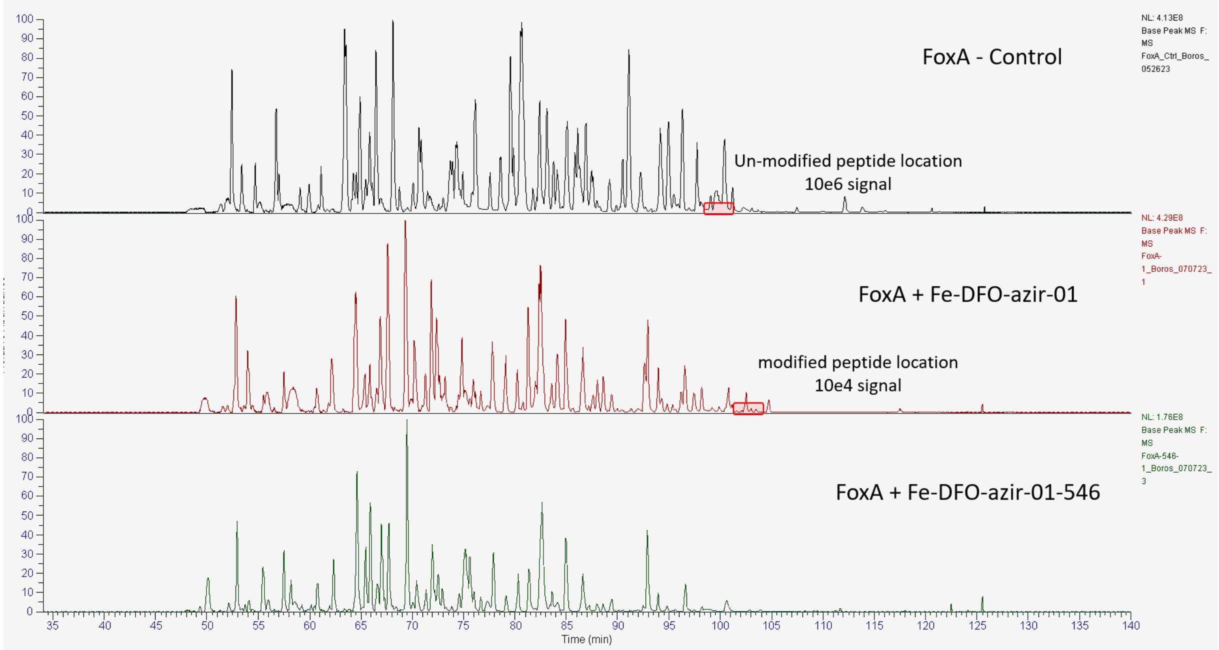

Figure S61. Fe-DFO-azir-01 base Peak Chromatogram traces of FoxA tryptic digest.

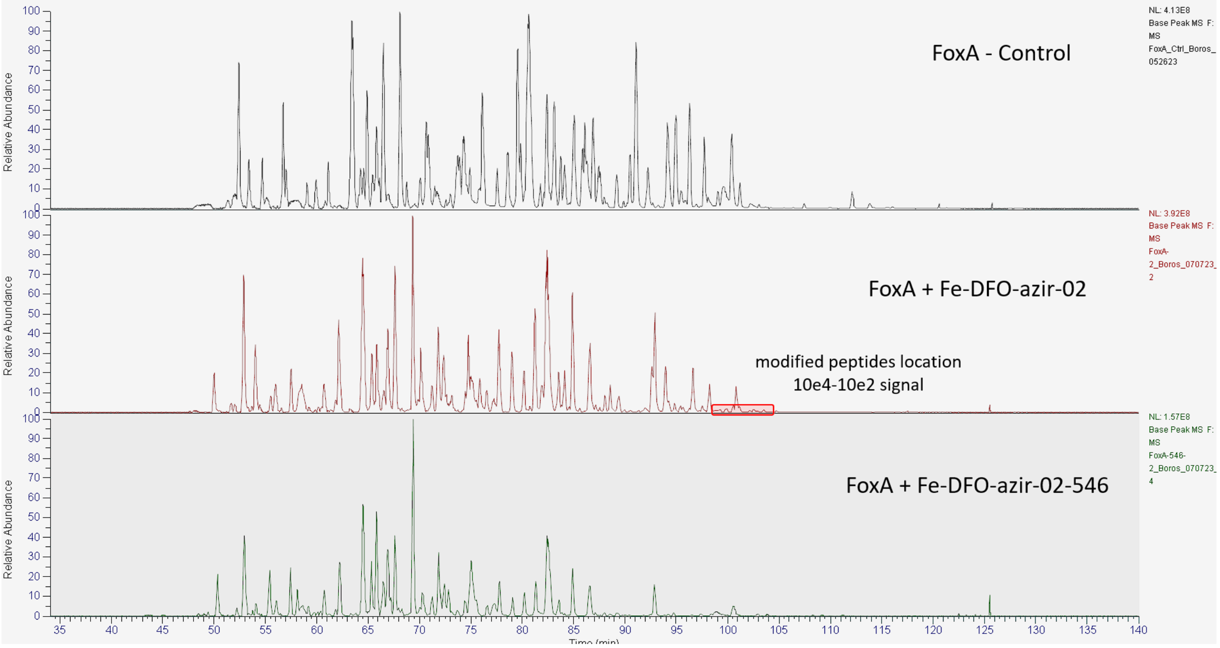

Figure S62. Fe-DFO-azir-02 base peak chromatogram traces of FoxA tryptic digest.

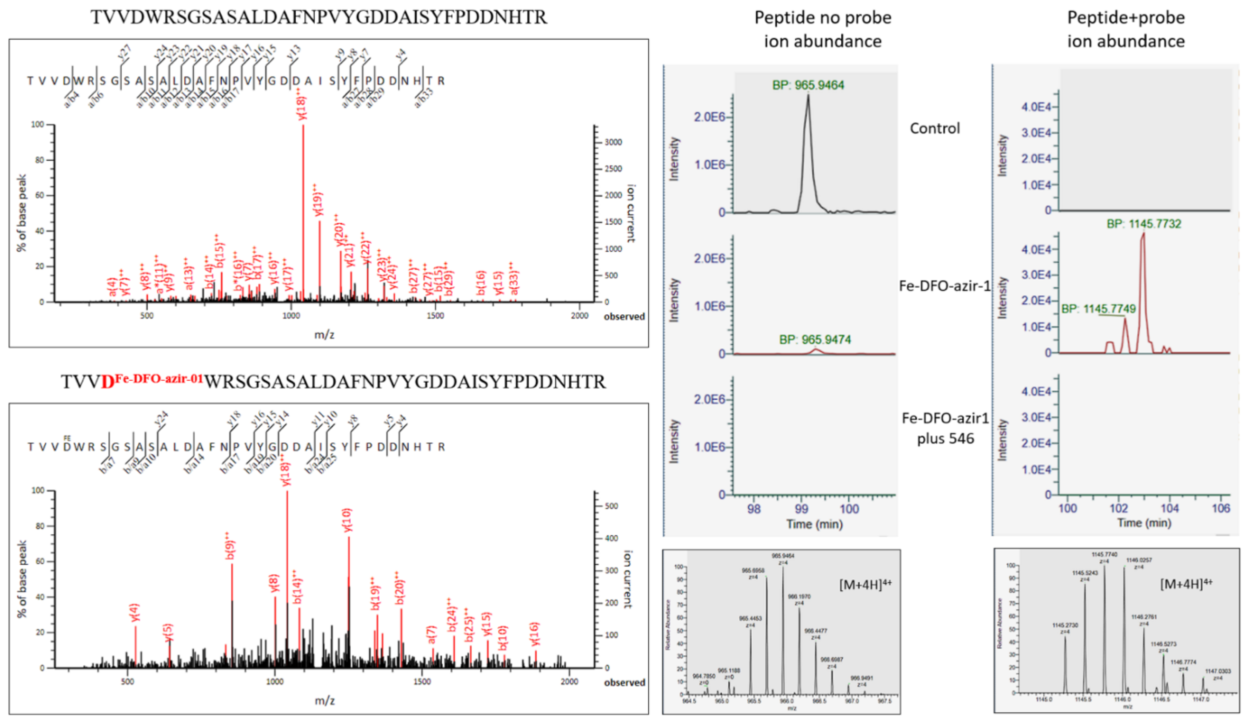

Figure S63. Site of labeling by **Fe-DFO-azir-01** photo-crosslinked of pure FoxA in solution.

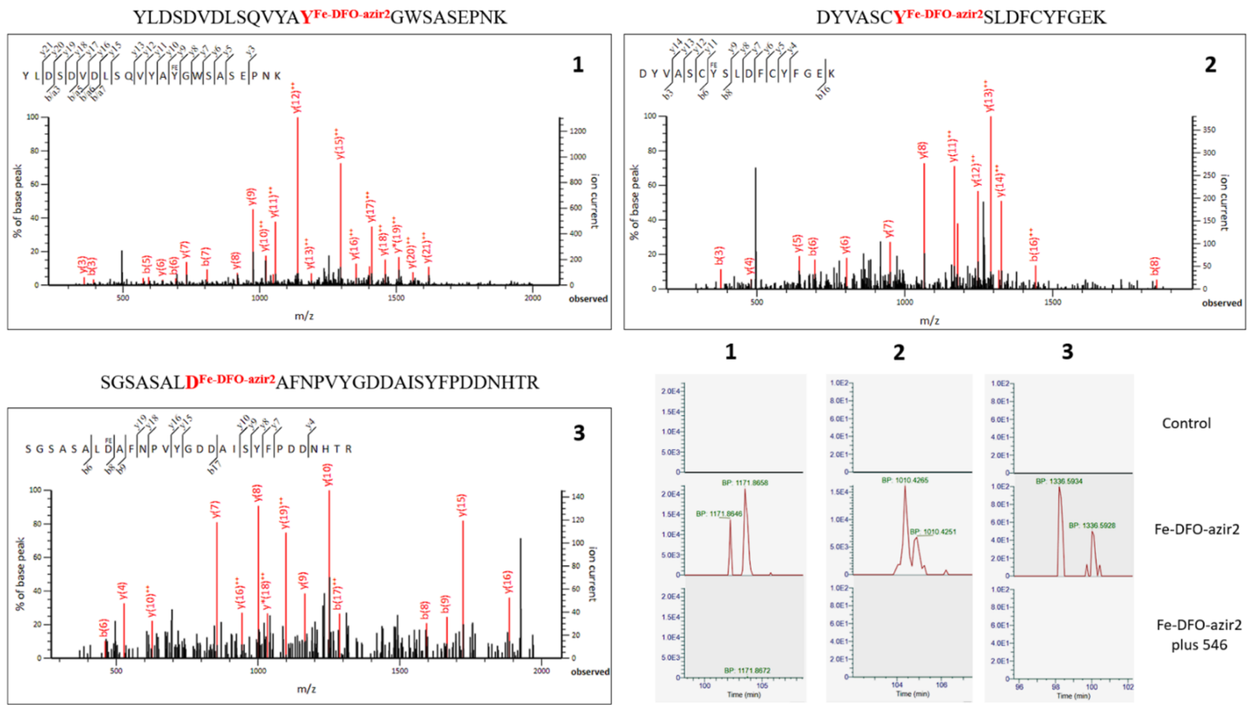

Figure S64. Site of labeling by **Fe-DFO-azir-02** photo-crosslinked to pure FoxA in solution.

DYVASCY^Ga-DFO-azir02^SLDFCYFGEK LNRYFSGARE^Ga-DFO-azir02^HLQAYIVDNMLQAEFATGAAR

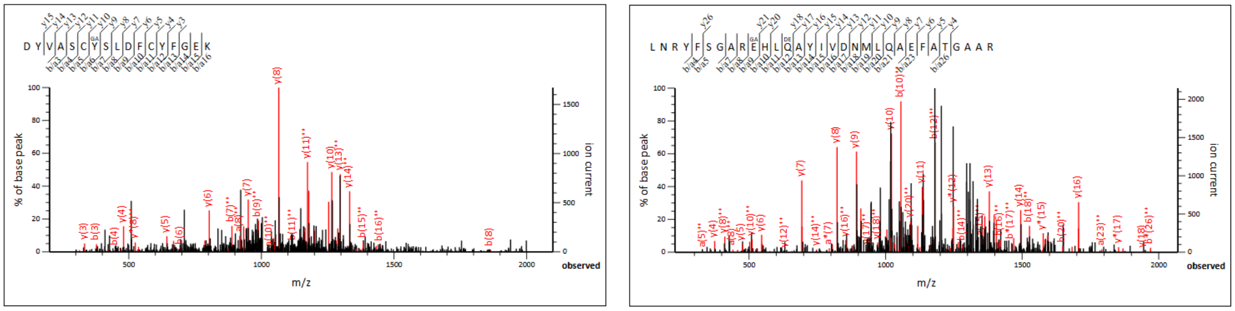

Figure S65. Site of labeling by **Ga-DFO-azir-02** photo-crosslinked to pure FoxA in solution.

A

Protein View: Q9I116

Ferrioxamine receptor FoxA OS=Pseudomonas aeruginosa (strain ATCC 15692 / DSM 22

Detailed information about this protein hit is shown below. (help)

Database: Boros_E_coli
Score: 12125
Monoisotopic mass (M_r): 90085
Calculated pI: 4.97

Sequence similarity is available as an NCBI BLAST search of Q9I116 against nr.

Search parameters

MS data file: \\STING\Users\mass spec\Users\Boros\2025_05\FoxA-Fe03_Boros_050125_15.mgf
Enzyme: Trypsin: cuts C-term side of KR unless next residue is P.
Fixed modifications: Carbamidomethyl (C)
Variable modifications: Deamidated (NQ), DFO-azir3 (DEY), Fe-DFO-azir3 (DEY), Oxidation (M)

Protein sequence coverage: 76%

Matched peptides shown in ***bold red***.

```
  1 MTATAVVLRN APSSLDFPRA SRLSRSVRAA LLSLAMAAGA APLCASAAEA
 51 AAEQARPYAI PAGQLGDVLN RFAREAGITL SATPAQTGGY SSQGLRGSFT
101 VQQGLARLLA DTPLEAEDQG DGSFVLREAP AKDGDVLNMQ AVEVFALGNN
151 LGSTDGYLAT HSQIATKTSK PLLETSQTVS VITREQIDDT ASKTVQQAMR
201 YTPGIFTGQV GASNRYDYVV MRGFADNSVD NIYLDGLKAM GDSGTFSSMQ
251 VDPYFLERID VLKGPSSVLY GRSLPGGLVA LTSKKPLYED YRQITGSIGN
301 MGQKEMGFDF SGPLDEEKRI AYRLIGLGKG SDTQFDHVKE ERYAIAPTLA
351 IDFSDDTTLT LQGYLQHDPN GGYHGGVPAD GTLSHHNGRH ISREFFDGEP
401 SKDDFDRTQR MFGYQLEHRI DDVWSARQNF RYLDSDVDLS QVYAYGWSAS
451 EPNKLNRYFS GAREHLQAYI VDNMLQAEFA TGAARHTLLT GLDYQRRRTV
501 VDWRSGSASA LDAFNPVYGD DAISYFPDDN HTRRLEQTGV YLQDLIDIDQ
551 WRFSLGLRQD WVSVTDKNRS TGSKADDDWE KFTGRIGALY LFDNGLAPYV
601 SYSESFNPNA YSDASGTPLA PTEGKQWELG LKFQAPGSNS FYTASLFHIT
651 QENVASKEPQ DNFYTSVGEV RSQGLELEAH TQLSDNLKLL GSYTYTDITY
701 TKSLDGNQGH TPNQAPKHMA SLWADYAFDA GPLSGLSIGG GARYVGETWA
751 DKENTLRVPD YTLVDARIGY DLGKLGLKGL DVSLNANNLL DKDYVASCYS
801 LDFCYFGEKR NVTATVNYQF
```

B LLADTPLE^Fe-DFO-azir3^AEDQGDGSFVLR

Peptide View

MS/MS Fragmentation of **LLADTPLEAEDQGDGSFVLR**
Found in **Q9I116** in **Boros_E_coli**, Ferrioxamine receptor FoxA OS=Pseudomonas aeruginosa (strain ATCC 15692 / DSM 22644 / C

Match to Query 21754: 3236.582298 from(1079.868042,3+) intensity(146628.5767) rtinseconds(6396.8788) index(16933)
Title: FoxA-Fe03_Boros_050125_15.23085.23085.3 File:"FoxA-Fe03_Boros_050125_15.raw", NativeID:"controllerType=0 control
Data file \\STING\Users\mass spec\Users\Boros\2025_05\FoxA-Fe03_Boros_050125_15.mgf

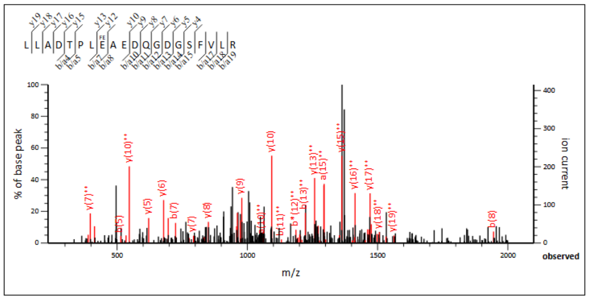

Figure S66. (A) Protein sequence coverage of pure FoxA in solution. **(B)** Site of labeling by **Fe-DFO-azir-03** photo-crosslinked to pure FoxA in solution. No match for the labeled peptide sequence was found in the sequence of native FoxA. No crosslinking was found for **Ga-DFO-azir-03**.

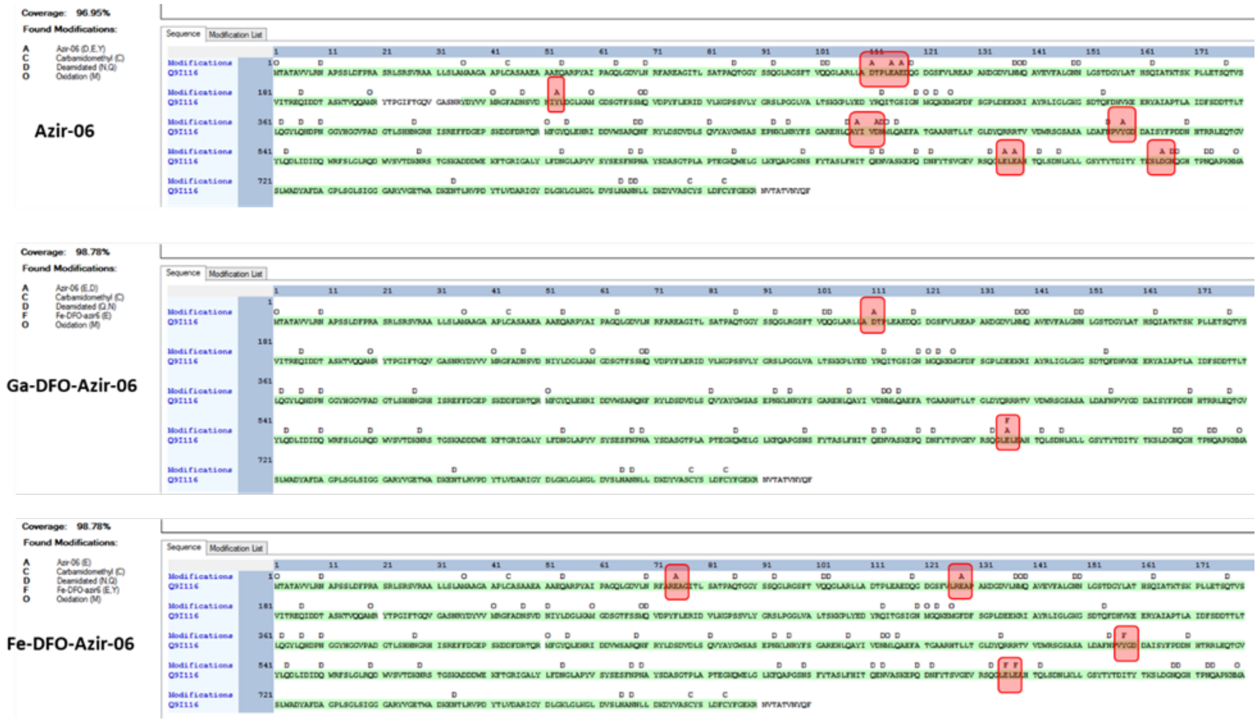

Figure S67. FoxA sites with probe modification after reacting with 50 eq of probes followed by 15min photo-irradiation at 365nm.

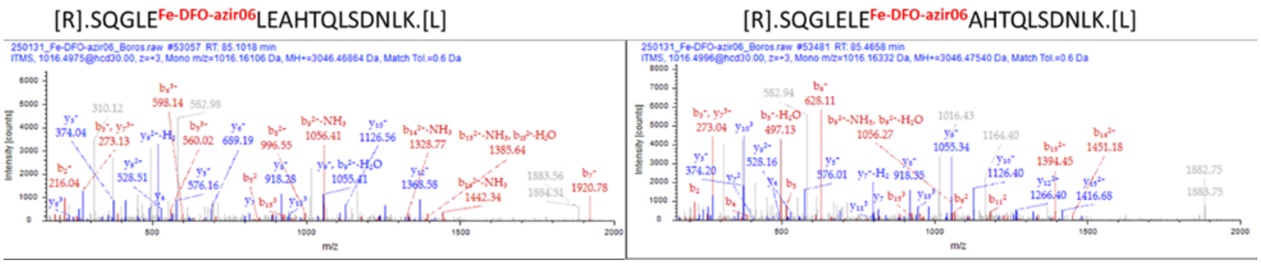

Figure S68. Site of labeling by **Fe-DFO-azir-06** photocrosslinked to pure FoxA in solution.

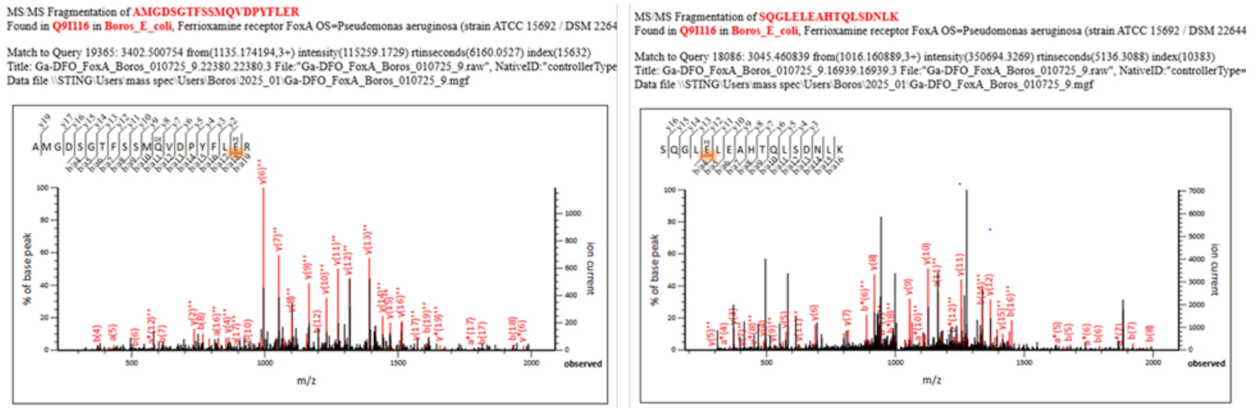

Figure S69. Site of labeling by **Ga-DFO-azir-06** photocrosslinked to pure FoxA in solution (found as **Fe-DFO-azir-06**).

6.3 Analysis of in-gel fluorescence bands

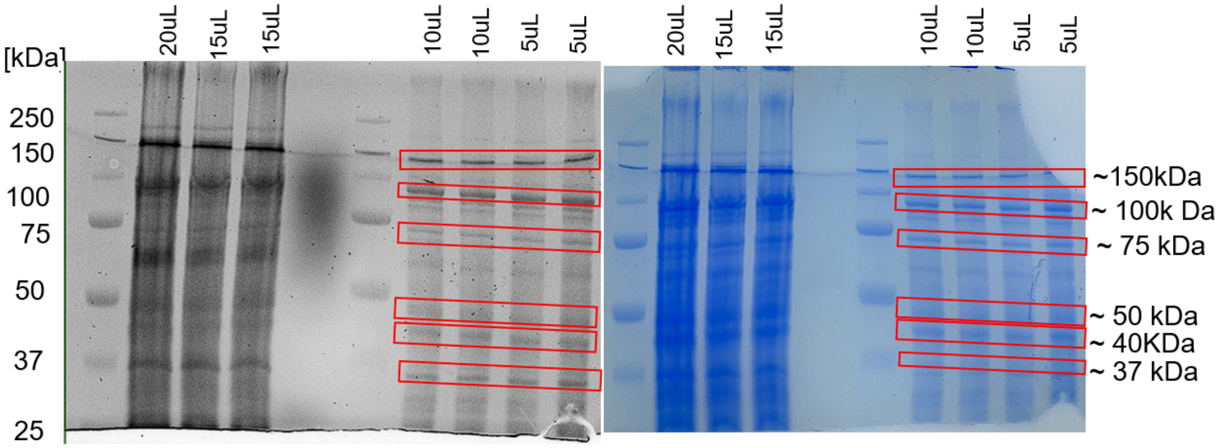

Figure S70. DFO-azir-05 gel used for MS/MS analysis of the fluorescent bands. Highlighted in red box were the band extracted for analysis.

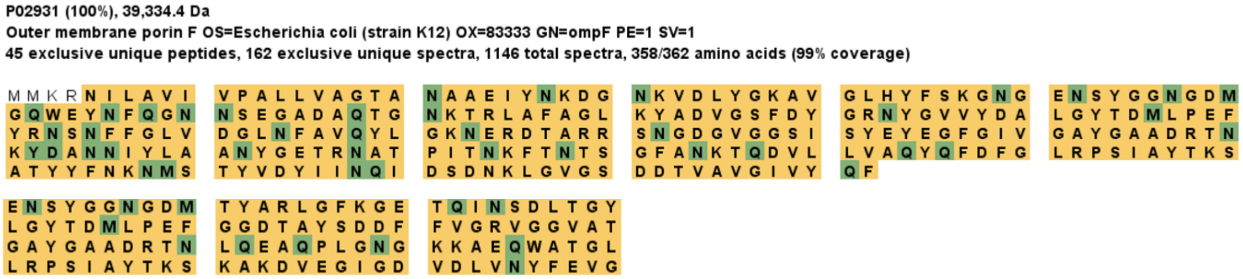

Figure S71. ~ 37kDa fluorescent gel band sequence coverage identification indicated the most abundant protein is outer membrane porin F (OmpF).

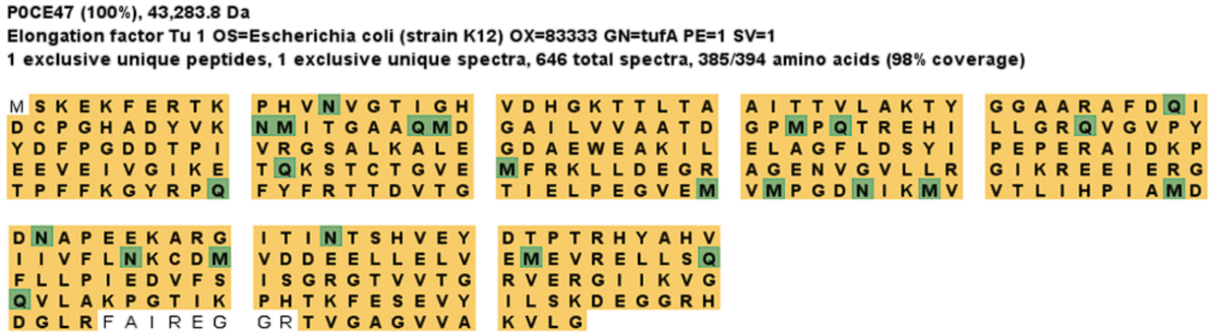

Figure S72. ~ 40kDa fluorescent gel band sequence coverage identification indicated the most abundant protein is Elongation factor Tu 1 (TufA).

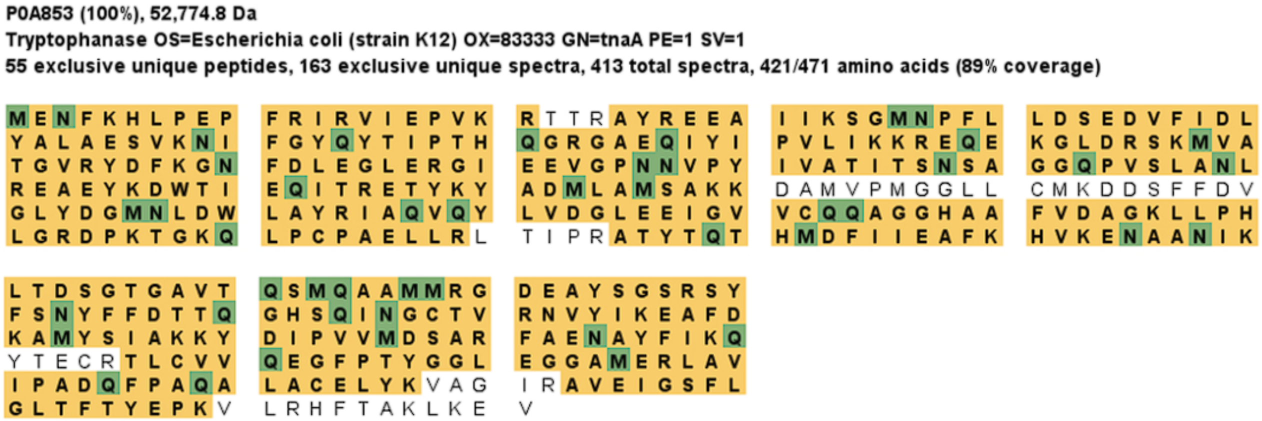

Figure S73. ~ 50kDa fluorescent gel band sequence coverage identification indicated the most abundant protein is tryptophanase (TnaA).

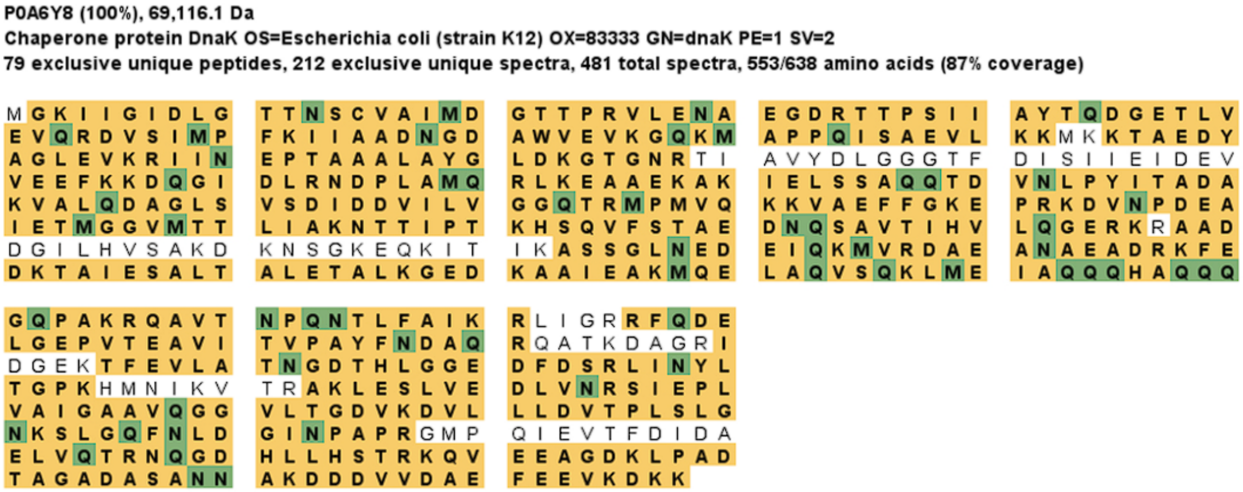

Figure S74. ~ 75kDa fluorescent gel band sequence coverage identification indicated the most abundant protein Chaperone protein DnaK.

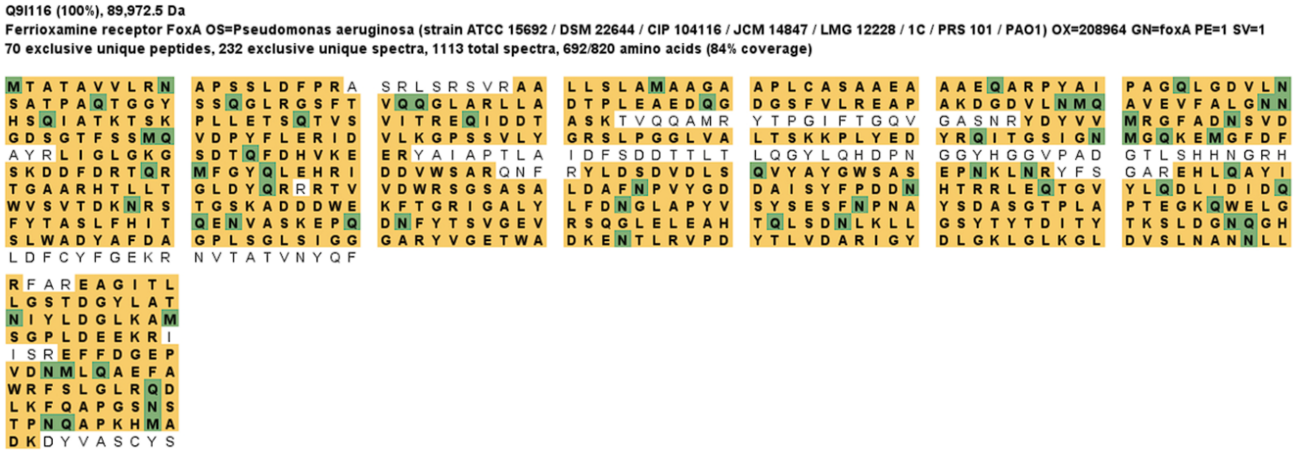

Figure S75. ~ 100kDa fluorescent gel band sequence coverage identification indicated the most abundant protein is outer membrane ferrioxamine receptor *FoxA*.

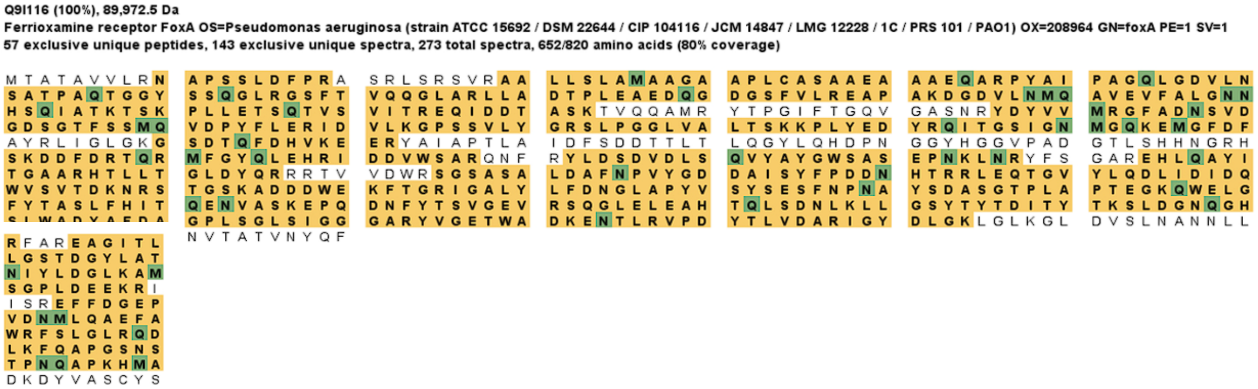

Figure S76. ~ 150kDa fluorescent gel band sequence coverage identification indicated the most abundant protein is outer membrane ferrioxamine receptor FoxA.

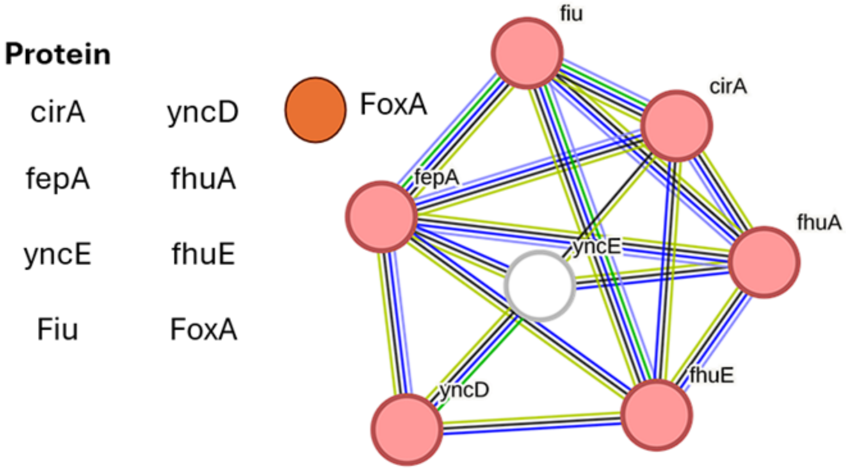

Figure S77. The most abundant protein identified by excision of gel bands and MS/MS for both **Fe-** and **Ga-DFO-azir-06** in **Figure S60**.

6.4 Analysis of enrichment experiments

Table S2. Spectral counts and statistics analysis of top 40 identified proteins in enrichment experiments conducted in *P. aeruginosa* PAO1 with **Ga-DFO-azir-06**, **Fe-DFO-azir-06** and **azir-biotin** control.

				Total Spectrum Count											
				azir			Fe06			Ga06			Welch T-Test (p-value)		
#	Accession Number	Alternate ID	Molecular Weight	azir-1	azir-2	azir-3	Fe06-1	Fe06-2	Fe06-3	Ga06-1	Ga06-2	Ga06-3	Fe06/azir	Ga06/azir	Ga06/Fe06
1	P37799	accB	16 kDa	486	179	355	401	143	232	413	867	206	0.2626891	0.262413	0.1760496
2	Q9HTD1	PA5435	66 kDa	49	11	30	1434	39	74	133	251	274	0.2005816	0.02115	0.2927217
3	Q9I299	liuD	71 kDa	4	0	0	960	3	2	36	9	6	0.2106229	0.120362	0.2202775
4	P72151	fliC	49 kDa	136	51	46	62	40	46	65	69	68	0.2178978	0.378682	0.0534028
5	Q9HT18	atpA	55 kDa	2	5	11	235	18	12	99	126	12	0.1891625	0.083541	0.4579728
6	P13794	oprF	38 kDa	65	42	60	10	34	41	31	120	57	0.0425596	0.330602	0.1285199
7	P30718	groL	57 kDa	84	30	32	34	36	19	49	138	3	0.1985987	0.379676	0.2428624
8	Q9HV43	dnaK	68 kDa	76	30	22	72	19	44	59	62	14	0.461639	0.461877	0.5
9	Q9HT20	atpD	50 kDa	0	2	3	238	4	5	64	91	2	0.2044474	0.097127	0.3720963
10	Q9I3D1	lpdG	50 kDa	69	39	57	25	10	43	39	58	30	0.0443813	0.175519	0.1331931
11	Q9I116	foxA	90 kDa	7	0	0	18	35	23	58	148	10	0.0144391	0.113415	0.1838656
12	Q9HWL3	fpvB	87 kDa	0	0	3	0	2	24	12	66	181	0.2120175	0.114464	0.1289452
13	Q9HWC9	rpoC	154 kDa	0	3	5	169	11	14	2	9	7	0.1783341	0.133547	0.1888164
14	P04739	pilA	16 kDa	39	23	19	23	15	19	23	35	30	0.1606709	0.380365	0.0390798
15	P09591	tufA	43 kDa	9	11	17	64	17	18	22	38	18	0.1566321	0.071251	0.3533006
16	Q59638	aceF	57 kDa	18	19	22	20	17	19	22	51	7	0.2710876	0.321321	0.2995224
17	P08308	arcB	38 kDa	62	13	11	22	7	13	20	19	6	0.2375165	0.251257	0.4405257
18	P11221	oprI	9 kDa	5	17	14	4	21	15	3	10	60	0.4199267	0.282675	0.3037739
19	Q9HWD2 [2]	fusA	78 kDa	0	3	5	52	12	9	24	26	7	0.1288299	0.053122	0.3748242
20	Q9I7C4	dnaN	41 kDa	25	3	0	54	4	8	15	38	0	0.2655116	0.287868	0.4180805
21	Q9I625	PA0493	9 kDa	62	0	0	20	0	11	13	32	0	0.3359762	0.409935	0.3478863
22	Q9I5I4	PA0745	30 kDa	42	5	5	11	5	12	29	22	0	0.2925006	0.491786	0.237784
23	Q51390	glpK2	56 kDa	18	9	13	15	10	15	13	24	0	0.5	0.451276	0.4500852
24	Q9I662	PA0456	8 kDa	13	13	33	12	10	12	7	24	8	0.1687404	0.242593	0.3957452
25	Q9I5D1	ampDh3	29 kDa	25	3	4	12	5	7	13	41	0	0.3753972	0.317753	0.2483323
26	Q9HZV6	atuF	72 kDa	0	0	0	112	0	0	0	0	0	0.2113249	#DIV/0!	0.2113249
27	Q9HWW1	oprG	25 kDa	20	18	12	13	14	10	16	14	2	0.1035299	0.156328	0.3721401
28	Q51561	rpoB	151 kDa	0	0	0	92	5	3	0	5	0	0.186851	0.211325	0.1966519
29	Q9I574	mliC	14 kDa	27	11	11	9	5	7	10	21	3	0.1092674	0.270121	0.2487267
30	Q9HTL9	PA5339	14 kDa	12	9	10	0	4	12	11	22	0	0.1450277	0.463181	0.2450745
31	Q9HW32	icmP	47 kDa	9	10	5	2	5	11	12	28	2	0.2783524	0.256617	0.2028418
32	Q9I4S1	PA1053	16 kDa	0	7	16	0	8	9	6	19	9	0.3675794	0.289708	0.1568058
33	G3XD11	oprH	22 kDa	10	11	3	7	5	9	8	25	4	0.3717207	0.290719	0.247988
34	Q9HVS6	PA4495	25 kDa	25	3	0	6	2	8	21	11	0	0.332744	0.450109	0.2380272
35	Q9I456	PA1288	46 kDa	10	8	3	10	4	13	4	18	0	0.293044	0.479313	0.4009567
36	Q9I473	PA1271	68 kDa	0	0	0	3	7	11	7	27	11	0.0468916	0.066747	0.1606709
37	Q9HXZ5	eno	45 kDa	16	4	0	5	0	10	8	24	0	0.3920805	0.333437	0.2587988
38	Q51567	sucD	30 kDa	5	6	6	21	4	8	7	18	3	0.203922	0.249853	0.4095184
39	P53593	sucC	42 kDa	0	2	0	26	5	5	7	16	0	0.1230732	0.134255	0.3183919
40	Q9I762	PA0070	32 kDa	8	13	5	0	4	6	3	16	0	0.0738129	0.349025	0.3063799

Table S3. Spectral counts and statistics analysis of top 40 identified proteins in enrichment experiments conducted in *E. coli* K-12 with **Ga-DFO-azir-06**, **Fe-DFO-azir-06** and **azir-biotin** control.

				Total Spectrum Count											
				azir			Fe-06			Ga-06			Welch T-Test (p-value)		
#	Accession Number	Alternate ID	Molecular Weight	azir1	azir2	azir3	Fe-06-1	Fe-06-2	Fe-06-3	Ga-06-1	Ga-06-2	Ga-06-3	Fe06/azir	Ga06/azir	Ga06/Fe06
1	P0ABD8	accB	17 kDa	112	495	536	606	361	698	339	716	1386	0.1820872	0.1469629	0.2466207
2	P04949	fliC	51 kDa	32	239	87	86	242	160	53	340	105	0.3021669	0.3449587	0.4876502
3	P0AEE5	mglB	36 kDa	33	261	112	53	195	73	28	252	58	0.3721698	0.4133109	0.4747223
4	P0A6Y8	dnaK	69 kDa	18	167	23	35	191	42	21	223	63	0.3954219	0.3485619	0.4394116
5	P0A6P9	eno	46 kDa	5	114	28	16	126	43	12	142	65	0.4001464	0.3291089	0.416311
6	P45523	fkpA	29 kDa	17	109	42	31	120	43	30	146	66	0.4177991	0.3028663	0.3681612
7	P21420 [3]	nmpC	40 kDa	31	248	116	80	116	128	54	270	135	0.3734189	0.4113549	0.2761372
8	P0A6F5	groL	57 kDa	10	13	10	25	112	13	16	29	24	0.1688103	0.0390988	0.2392475
9	P0A7K2	rplL	12 kDa	4	80	14	7	86	35	7	98	18	0.3891876	0.417238	0.4830877
10	P02925	rbsB	31 kDa	10	119	55	20	86	55	14	130	37	0.4238719	0.4921145	0.4393979
11	P0AFH8	osmY	21 kDa	13	88	25	13	74	18	8	129	25	0.414713	0.4014396	0.3428345
12	P02943	lamB	50 kDa	18	146	36	46	73	38	32	169	73	0.3792427	0.3437467	0.2202489
13	P60624	rplX	11 kDa	2	41	12	4	73	15	6	40	12	0.3234499	0.4761554	0.3339105
14	P75780	fiu	82 kDa	18	54	8	21	56	6	26	83	31	0.4815952	0.2179898	0.2328228
15	P17315	cirA	74 kDa	24	69	17	42	50	21	44	92	70	0.4800632	0.1054416	0.0722446
16	P69783	crr	18 kDa	0	37	4	4	50	5	4	61	9	0.385445	0.3213611	0.4218086
17	P0A6P1	tsf	30 kDa	2	7	4	9	44	4	3	10	13	0.181923	0.1415409	0.2503043
18	P0ABT2	dps	19 kDa	20	60	69	36	43	61	39	89	191	0.4349637	0.1665498	0.1564586
19	P0A7A9	ppa	20 kDa	4	51	12	11	43	30	12	51	11	0.3807775	0.4555178	0.4236651
20	P0A9Y6	cspC	7 kDa	0	37	25	9	42	36	5	55	13	0.3028809	0.4285237	0.407733
21	P0A805	frr	21 kDa	6	32	5	11	38	6	9	48	9	0.3893908	0.3272621	0.4171987
22	P0AF93	ridA	14 kDa	3	53	5	6	37	18	5	64	22	0.5	0.3490289	0.3235608
23	P06959	aceF	66 kDa	6	23	3	9	37	6	7	34	4	0.3018614	0.3629707	0.4366447
24	P0ACF4	hupB	9 kDa	0	17	4	0	36	4	4	69	4	0.3248417	0.2411451	0.3243804
25	P0AFK9	potD	39 kDa	7	61	12	7	34	7	10	38	13	0.3105816	0.3826698	0.3744982
26	P76116	yncE	39 kDa	19	16	18	24	33	6	24	15	28	0.358033	0.174173	0.4448819
27	P0A6F9	groS	10 kDa	5	44	7	21	33	20	8	51	11	0.3450542	0.4080667	0.4669254
28	P05825	fepA	82 kDa	14	15	10	12	32	5	13	38	28	0.3612188	0.1019979	0.2051629
29	P11557	damX	46 kDa	10	39	18	6	32	10	10	46	20	0.310548	0.4193842	0.2640736
30	P06996 [4]	ompC	40 kDa	25	77	14	50	30	10	30	77	56	0.3625256	0.2744322	0.1229626
31	P0AEX9	malE	43 kDa	34	83	45	41	28	17	40	46	57	0.1124897	0.359273	0.0484847
32	P0A9X9 [2]	cspA	7 kDa	0	19	3	4	28	12	0	37	6	0.2355683	0.3125193	0.490829
33	P00448	sodA	23 kDa	0	21	4	0	28	5	3	30	15	0.4086995	0.2461906	0.3448427
34	P0A6T9	gcvH	14 kDa	0	0	0	2	28	0	0	37	0	0.1914912	0.2113249	0.4433296
35	P16869	fhuE	81 kDa	2	0	0	49	26	4	118	26	102	0.0932757	0.0515754	0.0894566
36	P0ABA0	atpF	17 kDa	8	50	12	10	26	24	9	63	17	0.4165302	0.3918358	0.3149159
37	P06971	fhuA	82 kDa	6	8	0	25	24	13	35	70	74	0.016147	0.0213867	0.0383693
38	P0A917	ompX	19 kDa	5	55	11	10	24	0	6	52	15	0.2650259	0.4881811	0.2349432
39	P0ACD4	iscU	14 kDa	2	13	7	4	24	12	2	50	11	0.2149642	0.2265548	0.3329945
40	P0CE47 (+1)	tufA	43 kDa	2	11	10	4	23	3	3	23	7	0.3830017	0.3283985	0.4581057

References

(1) Domínguez-Vera, J. M. Iron(III) complexation of Desferrioxamine B encapsulated in apoferritin. *J Inorg Biochem* **2004**, *98* (3), 469-472. DOI: 10.1016/j.jinorgbio.2003.12.015 From NLM.

(2) Danilovtseva, E. N.; Pal'shin, V. A.; Krishnan, U. M.; Annenkov, V. V.; Zelinskiy, S. N. Tagging synthetic polymers with coumarin group for study nucleic acid interaction with gene delivery agents. *MethodsX* **2019**, *6*, 212-218. DOI: 10.1016/j.mex.2019.01.008 From NLM.

(3) Reynolds, G. A.; Drexhage, K. H. New coumarin dyes with rigidized structure for flashlamp-pumped dye lasers. *Optics Communications* **1975**, *13* (3), 222-225. DOI: https://doi.org/10.1016/0030-4018(75)90085-1.

(4) Kleiner, P.; Heydenreuter, W.; Stahl, M.; Korotkov, V. S.; Sieber, S. A. A Whole Proteome Inventory of Background Photocrosslinker Binding. *Angew. Chem., Int. Ed. Engl.* **2017**, *56* (5), 1396-1401. DOI: https://doi.org/10.1002/anie.201605993.

(5) Kabsch, W. Xds. *Acta Crystallogr D Biol Crystallogr* **2010**, *66* (Pt 2), 125-132. DOI: 10.1107/S0907444909047337

S0907444909047337 [pii].

(6) Evans, P. R. An introduction to data reduction: space-group determination, scaling and intensity statistics. *Acta Crystallogr D Biol Crystallogr* **2011**, *67* (Pt 4), 282-292. DOI: 10.1107/S090744491003982X.

(7) Josts, I.; Veith, K.; Tidow, H. Ternary structure of the outer membrane transporter FoxA with resolved signalling domain provides insights into TonB-mediated siderophore uptake. *Elife* **2019**, *8*. DOI: 10.7554/eLife.48528.

(8) McCoy, A. J.; Grosse-Kunstleve, R. W.; Adams, P. D.; Winn, M. D.; Storoni, L. C.; Read, R. J. Phaser crystallographic software. *J Appl Crystallogr* **2007**, *40* (Pt 4), 658-674. DOI: 10.1107/S0021889807021206.

(9) Afonine, P. V.; Grosse-Kunstleve, R. W.; Echols, N.; Headd, J. J.; Moriarty, N. W.; Mustyakimov, M.; Terwilliger, T. C.; Urzhumtsev, A.; Zwart, P. H.; Adams, P. D. Towards automated crystallographic structure refinement with phenix.refine. *Acta Crystallogr D Biol Crystallogr* **2012**, *68* (Pt 4), 352-367. DOI: 10.1107/S0907444912001308

S0907444912001308 [pii].

(10) Murshudov, G. N.; Skubak, P.; Lebedev, A. A.; Pannu, N. S.; Steiner, R. A.; Nicholls, R. A.; Winn, M. D.; Long, F.; Vagin, A. A. REFMAC5 for the refinement of macromolecular crystal structures. *Acta Crystallogr D Biol Crystallogr* **2011**, *67* (Pt 4), 355-367. DOI: 10.1107/S0907444911001314.

(11) Wang, J.; Wolf, R. M.; Caldwell, J. W.; Kollman, P. A.; Case, D. A. Development and testing of a general amber force field. *J Comput Chem* **2004**, *25* (9), 1157-1174. DOI: 10.1002/jcc.20035.

(12) Lee, C.; Yang, W.; Parr, R. G. Development of the Colle-Salvetti correlation-energy formula into a functional of the electron density. *Phys Rev B Condens Matter* **1988**, *37* (2), 785-789. DOI: 10.1103/physrevb.37.785.

(13) Becke, A. D. Density-functional thermochemistry. III. The role of exact exchange. *The Journal of Chemical Physics* **1993**, *98*, 5648-5652. DOI: 10.1063/1.464913

(14) Grimme, S.; Ehrlich, S.; Goerigk, L. Effect of the damping function in dispersion corrected density functional theory. *J Comput Chem* **2011**, *32* (7), 1456-1465. DOI: 10.1002/jcc.21759.

(15) Miertuš , S.; Scrocco, E.; Tomasi, J. Electrostatic interaction of a solute with a continuum. A direct utilizaion of AB initio molecular potentials for the prevision of solvent effects. *Chemical Physics* **1981**, *55* (1), 117-129. DOI: 10.1016/0301-0104(81)85090-2.

(16) Jo, S.; Kim, T.; Iyer, V. G.; Im, W. CHARMM-GUI: a web-based graphical user interface for CHARMM. *Journal of Computational Chemistry* **2008**, *29* (11), 1859-1865. DOI: 10.1002/jcc.20945.
